# National palliative care laws in Europe: do they enable access and effectively promote the development of palliative care within health systems? A comparative multimethod analysis

**DOI:** 10.1093/eurpub/ckag147

**Published:** 2026-08-27

**Authors:** Timo P Carpén, Laura Monzón-Llamas, Breffni L Hannon, Leszek Pawłowski, Tiina H Saarto, Reino T Pöyhiä, Carlos Centeno

**Affiliations:** Palliative Care Center, Comprehensive Cancer Center, Helsinki University Hospital and Faculty of Medicine, University of Helsinki, Helsinki, Finland; Department of Supportive Care, Princess Margaret Cancer Centre, University Health Network, Toronto, ON, Canada; ATLANTES Global Observatory of Palliative Care, University of Navarra, Institute for Culture and Society (ICS), Pamplona (Navarra), Spain; Department of Supportive Care, Princess Margaret Cancer Centre, University Health Network, Toronto, ON, Canada; Department of Palliative Medicine, Medical University of Gdańsk, Gdańsk, Poland; Palliative Care Center, Comprehensive Cancer Center, Helsinki University Hospital and Faculty of Medicine, University of Helsinki, Helsinki, Finland; Department of Clinical Medicine, University of Eastern Finland, Kuopio, Finland; ATLANTES Global Observatory of Palliative Care, University of Navarra, Institute for Culture and Society (ICS), Pamplona (Navarra), Spain

## Abstract

Access to palliative care (PC) is a fundamental right. While an increasing number of European countries have adopted national PC laws, it remains unclear how effective these laws are in advancing access and enabling development within health systems. This was a comparative multimethod study combining systematic analysis of national PC legislation with semi-structured interviews with national experts. Legal texts were analysed using a standardized extraction framework aligned with the WHO PC development model. Interview data provided interpretive context, contextualized legislative intent, and perceived system-level reach of laws. Fourteen countries had a national legal mandate explicitly addressing PC, either through standalone laws or binding frameworks within the health system. Laws varied widely in their aspiration and operational strength. Although almost half (6/14, 43%) explicitly recognized PC as a right, only France and Italy combined rights-based recognition with explicit mechanisms for implementation, governance, and financial sustainability. National experts linked stronger operational design with greater service development and integration, whereas predominantly declarative laws had limited practical relevance. Across countries, gaps were greatest in provisions related to research, access to essential medicines, and workforce development. National laws can function as important public health instruments to support access to PC and foster system-level development; enforceable implementation mechanisms and integration within health systems are crucial. Legislation should be understood as an enabling instrument whose impact depends on governance, financing, political commitment and health-system implementation.

## Introduction

Palliative care (PC) is increasingly recognized as an essential component of universal health coverage, and an integral element of the right to health [1–6]. By addressing physical, psychosocial, and spiritual suffering associated with serious illness, timely PC improves patient outcomes, including quality of life, for patients and their families [7–9]. A substantial body of evidence demonstrates that timely PC provision also reduces avoidable use of acute health services at the end of life, with important implications for health system performance [10–12]. Projections suggest that the burden of serious health-related suffering requiring PC will almost double globally by 2060, posing a major challenge for health systems and reinforcing the need for structural policy responses [13].

Despite this evidence and growing recognition of need, access to PC remains highly uneven across countries and within health systems. In Europe, marked variation persists in service availability, workforce capacity, and the integration of PC into mainstream health services. According to the European Association for Palliative Care (EAPC) Atlas of Palliative Care in the European Region 2025, provision ranges from advanced integration in some countries to fragmented or limited availability in others, reflecting differences in policy prioritization, governance, and resource allocation [1].

Over the past two decades, several European countries have sought to address these gaps through the adoption of national legal frameworks explicitly regulating PC. Such legislation has the potential to function as a powerful health system instrument, recognizing PC as a right, defining entitlements and obligations, structuring governance and financing mechanisms, and embedding PC within national health systems [14, 15]. Earlier comparative work demonstrated substantial heterogeneity in how European countries regulate the delivery of PC through laws, regulations, and national strategies, as well as wide variation in the scope, legal enforceability, and operational content of these instruments [3, 4]. However, important knowledge gaps remain. First, previous studies have largely focused on mapping the existence of legal and policy instruments, rather than analysing their content and normative ambition. Second, little is known about whether national PC laws effectively combine symbolic recognition of rights with concrete implementation mechanisms capable of fostering system-level development. Third, the perspectives of national experts in PC development and legislative processes have never been integrated into comparative legal analyses, limiting understanding of how laws are perceived and function in practice.

This study aims to examine national PC laws in Europe through a comparative multimethod approach. Specifically, it explores whether and how national legal mandates support access to and promote the development of PC within health systems. By combining systematic analysis of legal texts with expert interviews, this study seeks to assess the role of PC legislation as a health system instrument, moving beyond descriptive mapping to examine normative strength, implementation mechanisms, and perceived added value across countries.

## Methods

### Study design and data sources

A comparative multimethod qualitative study was conducted, combining systematic document analysis of national legal instruments regulating PC with semi-structured interviews with national experts. Methodological integration focused on complementarity and interpretative triangulation, ensuring epistemological consistency and analytical transparency [16]. The objective was to examine how binding national legal instruments in Europe address access to PC and their potential role as instruments for PC development within health systems, rather than to evaluate implementation outcomes or service performance. The aim was to focus on national legal instruments whose content primarily pertained directly to PC. Therefore, we did not analyse the entire legal system of each country nor investigate the extent to which general legal acts concerning healthcare may affect PC, including access to care, availability of essential medicines or regulation of futile therapy and assisted dying.

### Working definition and case selection

For the purposes of this study, the term ‘national palliative care law’ was used as a working definition to refer to the highest-level binding national legal instrument explicitly regulating PC in each country, regardless of its formal legal denomination (e.g. standalone law, amendment to health legislation or another binding national legal framework). Only legislation of general and binding effect was included in this study, comprising primary legislation enacted by national legislatures (i.e. acts of parliament) and secondary (delegated) legislation issued by governmental authorities under statutory powers. The rationale for including standalone national PC laws, other highest-level binding national legal mandates, and delegated legislation was to capture the full range of binding legal instruments through which PC is regulated, reflecting variation in legislative structures across countries. Although standalone national PC laws and delegated legislation are solely focused on PC, the other highest-level binding national legal mandates capture the same regulatory aspects. The included instruments are characterized by their general binding force, national legal authority, and regulatory function in establishing rights, obligations, entitlements, or organizational frameworks relevant to PC. Administrative measures operating solely within public authorities, including ministerial directives and internal regulations, were excluded. The texts of legally binding legislation in force as of 15 May 2025 were included in the analysis.

All nine European countries with standalone national PC laws identified in the EAPC Atlas of Palliative Care in the European Region 2025 [1] were used as the initial sampling frame (France, Belgium, Israel, Luxembourg, Italy, Portugal, Albania, Germany, and Greece). In parallel, we conducted an independent full-text review of national legislation across European countries, complemented by consultation with national PC experts. This process identified five additional countries with binding national legal instruments that met our working definition but were not classified as standalone PC laws in the Atlas: Poland, Armenia, Romania, Slovakia, and Austria. In total, 14 countries were included in the final analysis. Regional laws, general health laws without explicit PC content, and non-binding national strategies, policy plans or guidance documents were excluded.

Since our analysis focused on PC–specific binding legal instruments, it did not constitute a comprehensive assessment of public policy, which encompasses broader governance actions, programmes, and system design beyond legislation.

### Data sources

Two primary data sources were used: (i) the full texts of national legal instruments regulating PC and (ii) semi-structured interviews with key informants with direct knowledge of PC development and the legislative process in each country.

### Legal texts

Official versions of the included legal instruments were retrieved from governmental or parliamentary sources. Where relevant, accompanying regulations or policy documents were consulted to clarify scope and intent. Legal instruments were treated as primary documentary sources and analysed as normative texts shaping health system organization, governance, and access to PC. Document analysis followed the READ approach, which involves four structured stages: preparing materials, extracting data, analysing data, and distilling key findings [17].

Because official English translations are rarely available for national legal instruments, extraction was conducted from the original-language versions of each document. When an English version existed (official or widely used), it was used to support verification and internal consistency checks, but not as the primary source for extraction. For legal instruments not available in English, translations were produced for research purposes as described below.

Data extraction was guided by the WHO PC development framework [5] and operationalized through a standardized extraction template to ensure consistency across countries (Supplementary Table S1). The template captured contextual and structural characteristics of each legal instrument (e.g. year of enactment, title, length, structure, scope, purpose, objectives, and principles); explicit recognition of the right to PC; and the legal definition of PC. Remaining characteristics were mapped onto the six domains of the WHO PC development model [5]: (i) societal and community empowerment; (ii) policies and governance; (iii) research; (iv) access to essential PC medicines; (v) education and training; and (vi) service development and integration within health systems. In addition, the template captured any legally relevant ethically sensitive provisions that frequently accompany PC regulation in Europe (e.g. palliative sedation, advance care planning, limitation of life-sustaining treatments, and requests for assisted death). The complete extraction matrix (31 characteristics per country) is provided in Supplementary Tables S2–S6.

### Expert interviews

To contextualize the legal analysis, semi-structured interviews were conducted with at least one informant from each country with recognized expertise in PC development and direct knowledge of the legislative process. Informants included senior clinicians, academic leaders, national PC advocates, and policy advisors. Familiarity with the legislative and health system context in each country was prioritized rather than representativeness of professional or stakeholder groups.

Interviews explored three predefined areas: (i) background and motivation for the legislation; (ii) perceived contribution of the legislation to PC development; and (iii) perceived impact of the legislation on access to PC. One-on-one interviews were conducted by three researchers (T.P.C., C.C., and L.M.-L.), lasted approximately 30 min, were audio-recorded with written informed consent, and transcribed verbatim. Each interview was synthesized into a concise country-level summary structured around the three guiding questions. Summaries were shared with the corresponding key informants for accuracy checking (member checking), and feedback was incorporated when provided. These summaries are provided in Supplementary Tables S7–S13.

### Analysis

Analysis followed a structured, iterative, and integrative process combining systematic document analysis of legal instruments with qualitative interpretation of expert interview data [18, 19]. A framework analysis approach developed for applied policy research was used to enable transparent comparison across countries using an *a priori* analytical framework while simultaneously allowing for iterative refinement when relevant content was not captured by initial categories.

Legal instruments were examined comprehensively, and relevant provisions were systematically extracted and categorized by the same three researchers who conducted the qualitative interviews (TC, CC, and LM). Using a standardized extraction template, the legal provisions were mapped onto 31 characteristics aligned with the domains of the WHO PC development framework (Supplementary Table S1). The complete extraction matrix (31 characteristics per country) is provided in Supplementary Tables S2–S6.

Qualitative interview data were used as an interpretive and contextual lens to clarify legislative intent, perceived added value, and country-specific facilitators or constraints, rather than as the basis for a standalone qualitative study or inductive thematic analysis. The original legal documents remained the primary source for the analyses, and all translated passages used in the manuscript were manually checked against the original legal texts by the research team. The role of the national experts was not to validate the translations themselves, but to review the interpretation of the legal framework, clarify the national legal context, and resolve any uncertainties regarding the meaning and practical implications of the legislation.

### Use of artificial intelligence

Artificial intelligence (AI) tools (ChatGPT, OpenAI) were used under direct researchers’ supervision (TC, CC, and LM) to support technical tasks, including producing working translations of non-English legal texts for research purposes, locating relevant legal passages, supporting transcription workflows, and structuring interview summaries. All AI-assisted outputs were manually reviewed and cross-checked against the original sources by the research team. AI was not used for data interpretation, analytical decision-making, or generation of scientific conclusions.

## Results

### Characteristics of the included laws and countries

Fourteen European countries with a national legal mandate explicitly addressing PC were included in the analysis, with no missing data. In order of the year of enactment, these comprised France, Belgium, Israel, Luxembourg, Italy, Portugal, Poland, Albania, Germany, Armenia, Romania, Greece, Slovakia, and Austria. Of these, nine (64.3%) represented standalone national PC laws while the remaining five (35.7%) represented the highest-level binding national legal mandate governing PC within the country’s health system. In total, 11 of the included countries (78.6%) were classified as high-income countries, while three (21.4%) were upper-middle-income countries. France enacted the earliest national PC law in 1999, followed by Belgium in 2002. The most recent legal instruments were adopted in Greece, Slovakia, and Austria (all in 2022). Table 1 provides country-level summaries, including the title of the legal instrument in English, the year of enactment, and the type of legal framework.

**Table 1:** National legal instruments on palliative care included in the analysis.

Country	Year of enactment	Legal instrument (English title)	Type of legal framework
France	1999 2016	Law No. 99-477 of 9 June 1999, guaranteeing the right to access palliative care Law No. 2016-87 of 2 February 2016 creating new rights in favour of patients and persons at the end of life Both legal frameworks above are part of the *Code de la santé publique.*	Standalone national palliative care law Standalone national palliative care law
Belgium	2002 (updated 2016)	Law on Palliative Care	Standalone national palliative care law
Israel	2005	The Dying Patient Law	Standalone national palliative care law
Luxembourg	2009	Law of 16 March 2009, concerning palliative care, advance directives, and end-of-life support	Standalone national palliative care law
Italy	2010	Law of 15 March 2010, No. 38: Provisions to Ensure Access to Palliative Care and Pain Therapy	Standalone national palliative care law
Portugal	2012; 2018	Law No. 52/2012 on the Fundamentals of Palliative Care Law No. 31/2018 on Rights at the End of Life	Standalone national palliative care law (updated framework)
Poland	2013	Regulation of the Minister of Health of 29 October 2013 on Guaranteed Health Care Services in the Field of Palliative and Hospice Care	Highest-level binding national legal mandate governing palliative care
Albania	2014	Law No. 138/2014 on Palliative Care in the Republic of Albania	Standalone national palliative care law
Germany	2015	Law to Improve Hospice and Palliative Care in Germany (Hospice and Palliative Care Act—HPG)	Standalone national palliative care law
Armenia	2017	On Approval of the Standard for the Provision of Palliative Medical Care and Services	Highest-level binding national legal mandate governing palliative care
Romania	2018	Order No. 253 of 23 February 2018 on the organization and authorization of palliative care services	Highest-level binding national legal mandate governing palliative care
Greece	2022	Law No. 5007: Integrated System for the Provision of Palliative Care	Standalone national palliative care law
Slovakia	2022	Act No. 267/2022 Coll. of 29 June 2022 amending and supplementing Act No. 576/2004 Coll. on Health Care, Services Related to the Provision of Health Care, and on Amendments to Certain Acts, as amended, and amending and supplementing certain other acts	Highest-level binding national legal mandate governing palliative care
Austria	2022	Federal Act on the Establishment of a Hospice and Palliative Care Fund (HosPalFG)	Highest-level binding national legal mandate addressing palliative care

### Motivation and legislative intent

According to national key informants, the primary motivations for PC legislation were to improve access to PC and to promote its structured development within national health systems. In Belgium and Germany, informants additionally highlighted societal and parliamentary debates on assisted suicide as an important contextual driver shaping the adoption and framing of PC legislation.

### Legal recognition of the right to PC

Most countries (11/14, 79%) framed access through enforceable entitlement to services within the health system; however, only six countries (43%) explicitly recognized PC as a fundamental right (Table 2). Legal frameworks in Israel, Romania, and Austria lacked a clear enforceable entitlement of PC.

**Table 2:** Palliative care as a right in legal documents based on three levels.

Country	Explicitly mentioned	It is implied	Creates enforceable entitlement or clear access to palliative care
France	Yes	Yes	Yes
Belgium	Yes	Yes	Yes
Israel	No	Yes	No
Luxembourg	Yes	Yes	Yes
Italy	Yes	Yes	Yes
Portugal	Yes	Yes	Yes
Poland	No	Yes	Yes
Albania	Yes	Yes	Yes
Germany	No	Yes	Yes
Armenia	No	Yes	Yes
Romania	No	Yes	No
Greece	No	Yes	Yes
Slovakia	No	Yes	Yes
Austria	No	Yes	No

Informants consistently described rights-based recognition as a powerful normative and advocacy tool, increasing the visibility of PC and strengthening requests for access by patients, families, and professionals. However, they also emphasized that legal recognition alone was insufficient to enable effective access in the absence of robust implementation mechanisms, sustained financing, and political commitment.

### Governance, financing, and implementation mechanisms

The strength and specificity of implementation mechanisms varied substantially across countries. Governance structures and policy leadership were addressed in most legal frameworks, with France and Italy presenting the most comprehensive approaches. Italy’s law designates the Ministry of Health as the responsible authority and establishes mandatory national funding mechanisms for PC. Key informants described the law as providing a strong political foundation for national development, while also noting persistent challenges related to regional implementation and oversight. In contrast, key informants from Germany, Armenia, and Slovakia reported that, despite references to financing, their legal frameworks lacked clearly articulated national governance or strategic coordination mechanisms, contributing to uneven implementation across regions.

### Domains of PC development addressed by national laws

Most legal frameworks included provisions related to societal and community empowerment, particularly family involvement, volunteering, and efforts to improve public awareness. The extent to which national PC laws address the WHO PC development dimensions is summarized in Table 3. Albania’s law promotes informed decision-making and the participation of families and volunteers, although informants reported limited public awareness and weak operationalization. Greece’s law explicitly promotes public awareness and the role of civil society organizations (non-governmental organizations, advocacy and community groups) and trained volunteers within interdisciplinary teams.

**Table 3:** Coverage of WHO palliative care development dimensions in national palliative care laws.

Dimension	France	Belgium	Israel	Luxembourg	Italy	Portugal	Poland	Albania	Germany	Armenia	Greece	Romania	Slovakia	Austria
Empowerment of people	Yes	No	Yes	Yes	Yes	Yes	No	Yes	Yes	Yes	Yes	Yes	No	Yes
Policy	Yes	Yes	Yes	Yes	Yes	Yes	Yes	Yes	Yes	No	Yes	Yes	Yes	Yes
Research	Yes	No	Yes	No	Yes	No	No	No	No	No	No	No	No	No
Medicines	No	No	No	No	Yes	Yes	Yes	Yes	No	Yes	No	Yes	No	No
Education	Yes	No	No	Yes	Yes	Yes	Yes	Yes	No	Yes	Yes	Yes	No	Yes
Services	Yes	Yes	No	Yes	Yes	Yes	Yes	Yes	Yes	Yes	Yes	Yes	Yes	Yes

Research was the least developed domain, with only France, Israel, and Italy (3/14, 23%) explicitly referring to PC research in their legal frameworks, and only in general terms. Key informants confirmed that these provisions had limited practical impact and were not accompanied by dedicated funding or implementation mechanisms.

Access to essential PC medicines was addressed in six countries (43%), with explicit reference to opioid availability in Italy, Albania, Armenia, and Romania. Romania provided the most detailed regulatory approach, mandating authorized providers to ensure access to medicines for pain and symptom control. Nevertheless, key informants emphasized persistent regulatory, educational, and cultural barriers limiting effective access in practice.

Education and professional training were addressed in most legal frameworks (10/14, 71%), mainly through provisions surrounding continuing education or specialty training programmes. However, undergraduate education was only addressed in the national law of France. Informants from Albania and Armenia highlighted severe workforce shortages as a major barrier to service provision, despite the existence of legal mandates.

Integration of PC services within health systems was addressed in almost all legal frameworks (93%). The key informant from Germany reported that legislation facilitated structured service provision in hospitals and nursing homes, whereas the key informant from Albania described ongoing difficulties integrating PC into primary care, particularly in rural areas. Paediatric PC was specifically addressed only in Italy and Armenia.

### Cross-cutting patterns between legislation and expert perspectives

**Figure 1:**
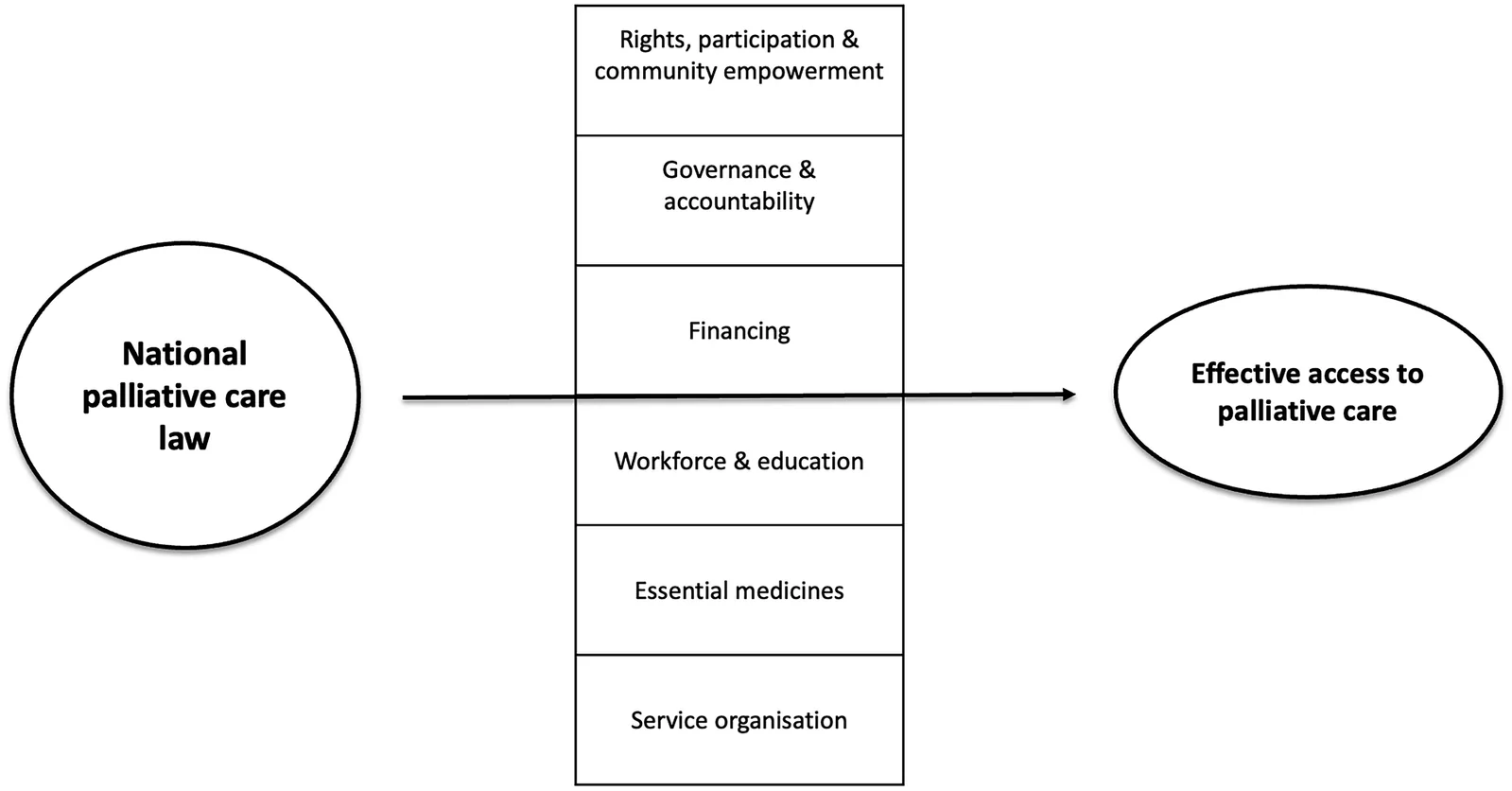
Conceptual model illustrating how national palliative care laws may contribute to effective access to palliative care mediated through rights, governance, financing, workforce, medicines, and service organization aligned with the WHO palliative care development framework.

Across countries, informants consistently emphasized that the impact of PC legislation depended not only on the legal text but also on political commitment, financing, and stakeholder engagement. Legal frameworks with strong normative recognition but limited operational detail were perceived as having modest transformative capacity, whereas more comprehensive and enforceable legal instruments were seen as more likely to facilitate PC development, even when implementation challenges persisted. Figure 1 illustrates a conceptual model of how national PC laws may support improved access to PC and enable PC development within health systems through key health system and policy domains, aligned with the WHO PC development framework.

## Discussion

This comparative multimethod analysis demonstrates variability across national PC laws in Europe in their potential to enable effective access to PC and to support its development within health systems. While most laws articulate an intention to improve access and support PC development, their normative ambition and operational strength differ substantially, shaping their perceived impact in practice.

Across countries, the motivations underpinning the adoption of PC legislation were broadly consistent. Key informants described these laws as responses to unmet needs in access to PC, gaps in service provision, and growing recognition of PC as a public health priority. In some contexts, particularly in Belgium and Germany, broader societal and parliamentary debates on assisted suicide acted as important catalysts, positioning PC legislation as a necessary precondition or counterbalancing response to controversial end-of-life dialogues. This finding highlights that PC laws often emerge not only from health system planning, but also from ethical, social, and political dynamics. Future work could formally examine temporal sequencing and co-evolution between PC legislation and assisted dying/euthanasia frameworks to test whether end-of-life controversies act as catalysts for PC legal mandates.

Explicit legal recognition of the right to PC appears to play a significant symbolic and advocacy role [14]. In countries where PC is framed as a fundamental right, key informants consistently perceived this recognition as strengthening the legitimacy of PC within the health system and supporting claims for access by patients, families, and healthcare professionals. However, this study also demonstrates that rights-based recognition alone is insufficient to enable effective access. Without clearly defined implementation mechanisms, dedicated financing, and accountable governance structures, the right to PC risks remaining largely declarative. This tension between normative recognition and operational capacity emerges as a central finding of this analysis.

When examined through the lens of the WHO PC development framework [5], national laws tended to be strongest in domains related to governance, policy alignment, and service integration, while systematically weaker in areas such as research and access to essential medicines. The limited attention to research reflects a persistent gap in legislative frameworks, despite the acknowledged role of research in guiding service quality, innovation, and system development. Similarly, the inconsistent and often superficial treatment of access to essential medicines, particularly opioid analgesics, highlights an enduring structural barrier [20, 21] that legislation has only partially addressed [21, 22]. These findings align with earlier European policy analyses documenting gaps between regulatory intent and effective system support for PC [3, 4].

Of note, paediatric PC was explicitly addressed only in the Armenian, Italian, and Polish legal frameworks. This finding is particularly concerning in light of recent evidence showing substantial global and regional gaps in the availability and integration of paediatric PC services within health systems [23, 24], and the crucial role of legislation in ensuring access to paediatric PC internationally [25].

The integration of expert interview data enabled a more nuanced interpretation of how legislation functions in practice and perceived system-level relevance. In countries with more comprehensive and operational legal frameworks, informants tended to describe legislation as a genuine enabler of PC development, even when implementation challenges persisted. Conversely, in settings where laws were predominantly declarative or weakly operationalized, informants reported limited practical impact despite formal legal recognition. When considered alongside the 2025 EAPC Atlas of Palliative Care in the European Region, these findings suggest that more robust legislative frameworks tend to align with more advanced levels of PC development, although legislation alone is neither a necessary nor a sufficient condition for progress [1].

Taken together, these results indicate that PC laws can function as effective instruments to enable access and foster development, but only under specific conditions. Laws appear most impactful when they combine explicit recognition of the right to PC with enforceable implementation mechanisms, stable financing, workforce development, access to essential medicines, and integration within national health system governance. In contrast, legislation that lacks operational detail or political backing is unlikely to produce substantive change.

These findings have direct implications for countries considering the adoption or revision of PC legislation. Rather than prioritizing symbolic recognition alone, policymakers should focus on designing laws that function as operational public health instruments. Explicit provisions on governance, financing, workforce training, access to essential medicines, and monitoring mechanisms are critical to translating legal commitments into real-world improvements. This need is particularly urgent given the substantial and growing burden of serious health-related suffering worldwide [13, 26].

This study has several strengths. Its multimethod design combines systematic legal analysis with expert perspectives, allowing a more comprehensive assessment of PC legislation as a health system instrument than legal analysis alone. However, limitations should also be acknowledged. First, the study focuses on national legal instruments and does not capture subnational regulations or informal governance mechanisms that may substantially influence PC development. Second, we did not examine supranational legal frameworks (e.g. human rights standards and EU-level regulatory environments) that may shape entitlements, enforceability, and implementation of PC, nor did we map the broader legal ecosystem, general health legislation, constitutional rights protection, financing and benefits package rules that can substantially determine how a PC mandate operates in practice. Third, implementation outcomes were not empirically measured, and findings rely on expert perceptions to interpret legislative intent and system-level relevance. Fourth, although the study includes all identified European countries with binding national PC legislation, the limited number of cases limits generalizability beyond the European context.

In conclusion, PC legislation can be a powerful tool to support access and promote development, but only when it moves beyond symbolic recognition. Laws appear to be most effective when they integrate rights-based recognition with operational mechanisms, financing, and accountability embedded within health system governance. Without this shift, legislation risks legitimizing PC in principle while failing to deliver it in practice. These insights are relevant for policymakers, legislators, and health system leaders seeking to design or reform PC laws that translate rights-based commitments into effective and equitable access to care.

## Supplementary Material

ckag147_Supplementary_Data

## Data Availability

The data are available from the principal author Carlos Centeno upon reasonable request.
