# Supplementary material for: National palliative care laws in Europe: do they enable access and effectively promote the development of palliative care within health systems? A comparative multimethod analysis
Source: Eur J Public Health. 2026 Aug 27;36(5):ckag147. doi: 10.1093/eurpub/ckag147 (PMC13521597; doi:10.1093/eurpub/ckag147)
Supplement: Supplementary file 1 [file ckag147_supplementary_data.docx]

**Supplementary appendix**

Table of Contents

[Supplementary Table S1 Structured Framework of Palliative Care Legislation Analysis: 31 Indicators with Working Definitions.	2](#_Toc236102995)

[Supplementary Table S2 Content Analyses of the National Palliative Care Legal Frameworks of France, Belgium and Luxembourg.	3](#_Toc236102996)

[Supplementary Table S3 Content Analyses of the National Palliative Care Legal Frameworks of Italy, Portugal and Germany.	18](#_Toc236102997)

[Supplementary Table S4 Content Analyses of the National Palliative Care Legal Frameworks of Armenia, Albania and Austria.	27](#_Toc236102998)

[Supplementary Table S5 Content Analyses of the National Palliative Care Legal Frameworks of Greece, Romania and Israel.	33](#_Toc236102999)

[Supplementary Table S6 Content Analyses of the National Palliative Care Legal Frameworks of Slovakia and Poland.	43](#_Toc236103000)

[Supplementary Table S7 Summary of Interviews with Key Informants of France and Belgium.	47](#_Toc236103001)

[Supplementary Table S8 Summary of Interviews with Key Informants of Luxembourg and Italy.	54](#_Toc236103002)

[Supplementary Table S9 Summary of Interviews with Key Informants of Portugal and Germany.	57](#_Toc236103003)

[Supplementary Table S10 Summary of Interviews with Key Informants of Armenia and Albania.	67](#_Toc236103004)

[Supplementary Table S11 Summary of Interviews with Key Informants of Austria and Greece.	73](#_Toc236103005)

[Supplementary Table S12 Summary of Interviews with Key Informants of Romania and Israel.	80](#_Toc236103006)

[Supplementary Table S13 Summary of Interviews with Key Informants of Slovakia and Poland.	84](#_Toc236103007)

# **Supplementary Table S1** Structured Framework of Palliative Care Legislation Analysis: 31 Indicators with Working Definitions.

| Domains | Indicators | Working Definition |
| --- | --- | --- |
| 1. Country Context | 1. WHO Region | According to WHO Regional Offices |
|  | 2. Income classification | Income level |
|  | 3. Level of palliative care development | Tripodoro VA, Fidalgo JFL, Pons JJ, et al. First-Ever Global Ranking of Palliative Care: 2025 World Map Under the New WHO Framework. J Pain Symptom Manage 2025;70:447-458 |
| 2. Formal Legal Characteristics | 4. Year of enactment | Enacted by national authority or parliament. |
|  | 5. Law title along with the reference | Original |
|  | 6. Law title in English | English |
|  | 7. Word count | Total number of words in the final version of the law. |
|  | 8. Structure | How the law is organized, major sections, chapters, and articles. |
|  | 9. Contents | Main topics regulated by the law |
| 3. Substantive Provisions | 10. Purpose | Fundamental reason or motivation behind the law |
|  | 11. Objectives | Measurable goals |
|  | 12. Principles | Ethical, legal, and conceptual foundations |
| 4. Right to PC | 13. Palliative care right | Right to receive palliative care (explicitly or implied) |
|  | 14. Palliative care definition | Including textual quotes where applicable. |
| 5. Alignment with WHO dimensions | 15. Society | Promotion of participation of individuals, families, and communities in palliative care development and decision-making about their health. |
|  | 16. Governance | Leadership and policy framework for implementing and regulating palliative care, ensuring proper access and funding. |
|  | 17. Research | Research in palliative care to improve scientific evidence and care. |
|  | 18. Medicines | Availability and access to palliative care essential medications |
|  | 19. Undergraduate education | Palliative care training into undergraduate educational programs. |
|  | 20. Continuing education | Continuing education in palliative care |
|  | 21. Specialization | Specialization in palliative care for physicians and other professionals. |
|  | 22. Integration of services | The national health system’s capacity to meet the needs of individuals experiencing health-related suffering, integrating primary and specialized care services. |
|  | 23. Specialized services | Organization and provision of specialized palliative care services for more complex patients. |
| 6. Related Legislation | 24. Palliative sedation | Palliative sedation |
|  | 25. Advance care planning | Advance care planning and decision-making for patients |
|  | 26. Adaptation of therapeutic effort | Adaptation of medical and palliative interventions according to patient needs, preferences, and desires. |
|  | 27. Requests for assisted death | Euthanasia, assisted suicide, or hastening death. |
| 7. Mechanisms for Implementation | 28. Standards | Norms and standards established by the law to ensure quality and consistency in palliative care. |
|  | 29. Financing | Funding mechanisms to cover the costs associated with implementing the law. |
|  | 30. Additional aspects | Any other relevant topics |
|  | 31. Subsequent regulations | Official documents issued after the original law to complement or develop its provisions. |

# **Supplementary Table S2** Content Analyses of the National Palliative Care Legal Frameworks of France, Belgium and Luxembourg.

| Indicators | France | Belgium | Luxembourg |
| --- | --- | --- | --- |
| 1. WHO Region | EURO | EURO | EURO |
| 2. Income classification | HIC | HIC | HIC |
| 3. Level of palliative care development | Advanced | Advanced | Advanced |
| 4. Year of enactment | 1999 and 2016 (two separate laws) | 2002 (updated in 2016) | 2009 |
| 5. Law title along with the reference | 1999 law: Loi n° 99-477 du 9 juin 1999 visant à garantir le droit à l'accès aux soins palliatifs (1)   <https://www.legifrance.gouv.fr/jorf/id/JORFTEXT000000212121>  2016 law: LOI no 2016-87 du 2 février 2016 créant de nouveaux droits en faveur des malades et des personnes en fin de vie (1)  <https://www.legifrance.gouv.fr/download/pdf?id=XE8PBLlDm87oKidSqvZlOQlgj8aUOv1MZCf1HPdWY3s=>  Both of the legal frameworks above are part of the Code de la santé publique.  <https://codes.droit.org/PDF/Code%20de%20la%20santé%20publique.pdf> | 2002: Loi relative aux soins palliatifs (1)  <https://www.ejustice.just.fgov.be/mopdf/2002/10/26_1.pdf#page=1>  2016 update: <https://www.ejustice.just.fgov.be/mopdf/2016/08/29_1.pdf#page=8> | "Loi du 16 mars 2009 relative aux soins palliatifs, à la directive anticipée et à l’accompagnement en fin de vie et modifiant diverses dispositions légales."  <https://legilux.public.lu/eli/etat/leg/loi/2009/03/16/n1/jo> |
| 6. Law title in English | 1999 law: Law No. 99-477 of June 9, 1999, guaranteeing the right to access palliative care (1)  2016 law: Law No. 2016-87 of 2 February 2016 creating new rights in favor of patients and persons at the end of life (1) | "Law on Palliative Care" | "Law of March 16, 2009, concerning palliative care, advance directives, and end-of-life support, and amending various legal provisions." |
| 7. Word count | 1999 law: 1368   2016 law: 2524 | 542 | 3521 |
| 8. Structure | 1999 law: The 1999 French palliative care law is structured into key articles: Article 1 establishes the right to palliative care, Articles 2-5 address regional plans, healthcare institutions, and professional contracts, and Article 6 mandates a report to Parliament. Articles 7-8 cover pain management responsibilities, Article 10 involves volunteers, and Articles 11-13 grant end-of-life leave for employees, creating a comprehensive framework for palliative care implementation.  2016 law: The law consists of 14 articles (Articles 1–14) and regulates end-of-life care by amending the Public Health Code: Articles 1–5 introduce core rights and provisions (including palliative care, refusal of treatment, and sedation), Articles 6–11 amend or repeal existing provisions (e.g., advance directives and trusted person), and Articles 12–14 address institutional aspects and territorial applicability. | Includes a preamble, Chapter I on constitutional basis, and Chapter II (Articles 2-10) detailing patient rights, government duties, care standards, and professional support. | The Palliative Care Law of Luxembourg consists of four chapters: Chapter I outlines the right to palliative care (Articles 1–3); Chapter II covers end-of-life wills and advance directives (Articles 4–8); Chapter III provides for leave to accompany end-of-life persons (Article 9); and Chapter IV contains amending and final provisions (Articles 10–14). |
| 9. Contents | 1999 law: The 1999 French palliative care law grants individuals the right to palliative care for serious health conditions (Article 1), defines it as an interdisciplinary approach focused on pain relief and dignity, and mandates that regional healthcare plans prioritize palliative care (Article 2). It requires public and private institutions to provide these services (Article 4), supports home-based care with specific provider contracts (Article 5), involves trained volunteers (Article 10), and grants family members leave for end-of-life support (Article 11).  2016 law: The law regulates end-of-life care by establishing provisions on relief of suffering, sedation, refusal or withdrawal of treatment, advance directives, and the trusted person, as well as palliative care training and reporting obligations. | Ensures the right to accessible, comprehensive palliative care for advanced illness, covering "physical, psychological, social, moral, existential, and spiritual" needs with family support (Article 2). It mandates criteria for funding (Article 3), regular reporting on care quality (Articles 4, 8), palliative teams in facilities (Article 5), patient rights awareness (Article 6), support from palliative care teams to health care professionals (Article 7), and complements existing laws (Article 9) with an effective date set by decree (Article 10). | Defines the right to palliative care [Article 1], regulates advance directives for end-of-life decisions [Articles 4–6], outlines physicians' responsibilities [Articles 2–3, 6], provides leave for accompanying end-of-life persons [Article 9], and amends related laws to integrate palliative care into the healthcare and social systems [Articles 10–14]. |
| 10. Purpose | 1999 law: The 1999 French law aims to guarantee the right to access palliative care for all individuals whose condition requires it, emphasizing the importance of alleviating pain, preserving dignity, and providing psychological and social support. This fundamental motivation is reflected in Article 1, establishing palliative care as a necessary component of ethical and compassionate healthcare for those at the end of life.  2016 law: The law aims to create new rights for patients at the end of life, including ensuring a dignified end of life with relief of suffering (Article 1), regulating medical practices and decision-making (Articles 2–5), establishing procedures for advance directives and trusted persons (Articles 8–10), and providing for implementation and evaluation of palliative care policy (Articles 12–14). | Aims to ensure the right to quality, accessible palliative care for patients in advanced illness, offering support for "physical, psychological, social, moral, existential, and spiritual" needs (Article 2). Rooted in ethical considerations, it upholds patient autonomy, and quality of life while promoting equitable access to end-of-life care within the healthcare system. | The purpose is to guarantee access to palliative care for individuals in advanced or terminal stages of serious illnesses, respecting their dignity and addressing their physical, psychological, and spiritual needs [Article 1]. |
| 11. Objectives | 1999 law: The 1999 French palliative care law aims to achieve specific goals, including establishing a regional framework for palliative care with designated units, mobile teams, and home hospitalization (Article 2). It also mandates that public and private healthcare institutions incorporate palliative care within their services, and emphasizes healthcare provider training to improve end-of-life care quality (Articles 4 and 7).  2016 law: The law regulates to ensure relief of suffering and dignity at the end of life (Articles 1 and 4), allow sedation (Article 3), and establish rights on refusal of treatment, advance directives, and trusted person (Articles 5, 8–10). | Ensures accessible, high-quality palliative care for patients "regardless of life expectancy," addressing "physical, psychological, social, moral, existential, and spiritual" needs (Article 2). It funds programs to ensure quality (Article 3), integrates care teams in facilities (Article 5), mandates quality reporting (Articles 4, 8), and supports healthcare professionals by specialized palliative care teams (Article 7). | The objectives are to ensure access to palliative care [Article 1], uphold dignity through coordinated care [Article 1], regulate advance directives [Articles 4–6], manage pain, including life-shortening treatments [Article 3], and provide leave for caregivers [Article 9]. |
| 12. Principles | 1999 law: The 1999 French palliative care law is grounded in principles of patient dignity, autonomy, and relief from suffering. It establishes the right for individuals whose health condition requires it to access palliative care, emphasizing pain relief, psychological support, and the preservation of dignity (Article 1). Additionally, the law upholds the patient's right to refuse treatment, reinforcing respect for patient autonomy in care decisions (Article 1, Art. L. 1er C).  2016 law: The law establishes principles of dignity at the end of life and relief of suffering (Article 1), prohibition of unreasonable obstinacy in treatment (Article 2), respect for the patient’s will and autonomy (Articles 5, 8–10), and provision of palliative care to ensure quality of life until death (Articles 2 and 4). | The law emphasizes patient autonomy, and equitable access through a holistic approach addressing "physical, psychological, social, moral, existential, and spiritual" needs (Article 2), establishing a right to palliative care grounded in ethical commitments to quality and support. | The law guides the ethical principles of human dignity [Article 1]. Every person with a terminall illness should have access to palliative care. |
| 13. Palliative care right | 1999 law: The Loi n° 99-477 of June 9, 1999, explicitly establishes the right to receive palliative care. This is clearly stated in Article 1, which affirms that “any person whose condition requires it has the right to access palliative care and support.”   2016 law: The law explicitly recognises a right related to palliative care. In the Article 1 the following is stated: “Every person has the right to have a dignified end of life accompanied by the best possible relief of suffering.” | The law explicitly establishes the right to receive palliative care for all patients in advanced stages of serious illness, regardless of life expectancy, as stated in Article 2 (2016 update). | Under the title of the right for PC, Article 1 states that every person in an advanced or terminal stage of a serious and incurable condition has access to multidimensional palliative care. |
| 14. Palliative care definition | 1999 law: "Palliative care consists of active and continuous care provided by an interdisciplinary team, either in an institution or at home. Their goal is to relieve pain, alleviate psychological suffering, preserve the dignity of the patient, and support their family." (Article L. 1er B, Loi n° 99-477).  2016 law: The law refers to “palliative care” (e.g., Article L.1110-5-1 and Article L.1110-10), but does not provide a formal definition within the text. | Defines palliative care as comprehensive support for patients in advanced illness, addressing "physical, psychological, social, moral, existential, and spiritual" needs to ensure quality of life and autonomy. | The law defines palliative care in Article 1 as "active, continuous, and coordinated care provided by a multidisciplinary team in respect of the dignity of the person being cared for." It aims to address the physical, psychological, and spiritual needs of the patient while supporting their loved ones and includes the management of pain and psychological suffering. |
| 15. Society | 1999 law: The law integrates society into palliative care by fostering active involvement from both families and trained volunteers, creating a compassionate support network for patients. It grants employees the right to take leave to care for terminally ill relatives (Article 11), supporting family engagement in end-of-life decisions. Additionally, the law authorizes volunteer associations to provide trained individuals to assist in palliative care (Article 10), emphasizing respect for patients’ dignity, privacy, and beliefs. This dual approach promotes a collaborative framework where families and communities contribute to the patient’s physical, psychological, and social well-being, enhancing the holistic nature of palliative care.  2016 law: The law regulates involvement of family, relatives, and the trusted person in decision-making (Articles 5, 8–10) and requires that patients are informed about the possibility of home care (Article 4), but does not explicitly address community or societal roles in palliative care. | The following sentence is in the 2016 update in Article 2: "Ideally, the provision of palliative care should progressively adapt to the needs and wishes of the patient, independent of their life expectancy". While this approach supports the patient's active participation in care decisions, it does not explicitly involve family members in this decision-making process. | The law promotes the participation of individuals and families by addressing individual autonomy through advance directives [Articles 4–6] and acknowledges the involvement of families primarily in care decisions and support, as seen in the provisions for trusted persons [Articles 1, 4, 9]. Nothing is addressed regarding community. |
| 16. Governance | 1999 law: The law establishes a robust leadership and policy framework that supports the effective implementation and regulation of palliative care, ensuring access and appropriate funding at the regional level (Articles 2 and 4). This framework can be compared to similar laws in other jurisdictions that also emphasize regional planning, interdisciplinary collaboration, and accountability in healthcare delivery. It is mentioned that "Government shall submit to Parliament a report on the consideration of palliative care within the framework of the program for the medicalization of the information system."   2016 law: The law assigns responsibilities to health professionals and physicians for implementing end-of-life decisions and ensuring palliative care (Articles 1–5), and requires the Government to submit an annual report to Parliament on the implementation of the law and palliative care policy (Article 14), but does not explicitly define a broader governance structure or roles of specific authorities. | An evaluation unit within the Scientific Institute for Public Health assesses care quality and needs, providing biennial reports to inform policy and ensure accountability (Article 8). In addition the governance requires annual reporting by Social Affairs and Public Health ministers to monitor implementation (Article 4). | The law assigns governance to the state by requiring it to organize training for care staff and mandate the use of a care logbook to document palliative care services, with specifics determined by Grand Ducal regulation [Article 1]. |
| 17. Research | 1999 law: Article 7. University hospitals ensure, in this respect, the initial and continuing training of healthcare professionals and disseminate, in cooperation with other public or private health establishments participating in the public hospital service, the knowledge acquired, including to the care teams, to allow the realization of these objectives in both community and hospital settings. They promote the development of research.  2016 law: Research is not addressed in the law. | Research is not explicity mentioned in the law; however, Article 8 establishes an evaluation unit to assess palliative care quality and needs, indirectly supporting evidence-based improvements through ongoing monitoring. | The law does not mention provisions to encourage or support research in palliative care. |
| 18. Medicines | 1999 law: The law does not explicitly list or define specific medications or access protocols.The law does not provide a dedicated definition or detailed guidance on the precise availability or regulation of palliative medications beyond this scope.  2016 law: The law refers to the use of analgesic and sedative treatments to relieve suffering, including in the context of deep and continuous sedation until death (Articles 3 and 4), but does not specifically regulate medicines, access to drugs, or opioids in detail. | The law does not address the availability or access to essential medications for managing pain and other symptoms in palliative care. | The law addresses the management of pain and psychological suffering as integral components of palliative care [Article 1]. However, it does not explicitly detail the availability or access to specific essential medications for symptom management, relying on general provisions for care delivery. |
| 19. Undergraduate education | 1999 law: The following is outlined in Article 7: "University hospitals ensure, in this respect, the initial and continuing training of healthcare professionals."  2016 law: The 2016 law mandates that initial and continuing training for physicians and nurses include teaching on palliative care. | The law does not mention incorporating palliative care training into undergraduate educational programs. | The law mandates the state to ensure adequate training in palliative care but does not explicitly address its integration into undergraduate educational programs. Training specifics are left to be determined by Grand Ducal regulations [Article 1]. Undergraduate training is not addressed in the law text. |
| 20. Continuing education | 1999 law: The following is outlined in Article 7: "University hospitals ensure, in this respect, the initial and continuing training of healthcare professionals."  2016 law: The 2016 law mandates that initial and continuing training for physicians, pharmacists, nurses, nursing assistants, home care assistants and clinical psychologists include teaching on palliative care. | The law does not include specific provisions for continuing education in palliative care for healthcare professionals. | Continuing education is not mentioned. |
| 21. Specialization | 1999 law: The law does not mandate a formal specialization or certification pathway specifically for palliative care, this focus on dedicated education and skill-building implies a foundational step toward developing expertise in this field for physicians and other healthcare professionals.  2016 law: Not addressed in the law. | The law does not contain provisions promoting specialization in palliative care for physicians or other healthcare professionals. | The law promotes specialization by requiring the state to organize specific medical training in palliative care for physicians and other healthcare professionals. The details of this specialized training are to be determined through Grand Ducal regulations [Article 1]. It is mentioned that "The State ensures appropriate training for medical and care staff. A Grand Ducal regulation establishes the organization of specific medical training in palliative care for doctors and other health professionals." |
| 22. Integration of services | 1999 law: The 1999 French law integrates palliative care by requiring regional healthcare plans to address palliative care needs, including units, mobile teams, and home hospitalization (Article 2). It also mandates that public and private health institutions organize palliative care within their services or through alternative structures like home-based care (Article 4).  2016 law: The law requires that palliative care is provided when treatments are withheld or withdrawn and throughout end-of-life care, including the possibility of care at home or in healthcare institutions (Articles 2–4), but does not explicitly regulate integration of services across different levels of care. | It is mentioned that palliative care is delivered in hospitals, in facilities covered under health and dependency insurance laws, or at home (Article 1). | The law integrates services by ensuring that palliative care is provided in hospitals, approved care institutions under health and dependency insurance, or at home, with close collaboration between different care providers, including hospitals for home or institutional care [Article 1]. |
| 23. Specialized services | 1999 law: The 1999 French law mandates that regional healthcare plans specify the resources needed for specialized palliative care, including dedicated units, mobile teams, and home hospitalization for complex cases (Article 2). Additionally, public and private health institutions must organize palliative care services either within their facilities or through alternative care structures (Article 4).  2016 law: The law does not explicitly define or regulate specialized palliative care services or units, but refers generally to the provision of palliative care. | Provides for specialized services by mandating the inclusion of palliative care teams within healthcare facilities, which are intended to support complex care needs and provide specialized assistance for patients facing advanced illness stages (Article 5). | This is not mentioned in the law. |
| 24. Palliative sedation | 1999 law: The 1999 French law on palliative care does not specifically address or regulate the use of palliative sedation.   2016 law: The law regulates deep and continuous sedation until death, including its indications, conditions, and procedures (Article 3) | The law does not address or regulate palliative sedation, and it does not include legal criteria or safeguards for its use. | The law does not mention or regulate palliative sedation. |
| 25. Advance care planning | 1999 law: The 1999 French law on palliative care does not include provisions for advance care planning.  2016 law: The law regulates advance directives, allowing any adult to express their wishes regarding end-of-life care, including continuation or withdrawal of treatment (Article 8). | The law does not address advance care planning or decision-making for patients in cases of incapacity. However, it is said that "Healthcare providers are responsible for educating patients and their families on palliative care options available to them." | The law facilitates advance care planning by allowing individuals to document their end-of-life wishes through advance directives and appoint a trusted person to make decisions if they become incapacitated [Articles 4–6]. |
| 26. Adaptation of therapeutic effort | 1999 law: The 1999 French law permits patients to refuse medical interventions, acknowledging their autonomy in decision-making (Article 1, Art. L.1er C). However, the law does not specifically address the broader adaptation of therapeutic or palliative efforts based on individual preferences and desires beyond this right to refuse treatments.  2016 law: The law regulates the limitation or withdrawal of treatment, in accordance with the patient’s will and through a collegial procedure (Articles 2 and 5). | Emphasizes that palliative care should begin when the patient is identified as needing it and continue progressively, "ideally adapting to the care needs and wishes" of the patient throughout their illness (Article 2, 2016 update). However, specific interventions are not addressed. | The law emphasizes that palliative care should be tailored to the patient's physical, psychological, and spiritual needs, respecting their autonomy and wishes as expressed through advance directives [Articles 4–6]. |
| 27. Requests for assisted suicide | 1999 law: The 1999 French law on palliative care does not address euthanasia, assisted suicide, or any requests for hastening death.  2016 law: Not addressed in the law. | The law does not address euthanasia, assisted suicide, or requests for hastening death, as these are covered under Belgium’s separate 2002 euthanasia law. | Not addressed in the palliative care law. However, Luxemburg has a separate law for Euthanasia and Assisted Suicide. |
| 28. Standards | 1999 law: The law requires healthcare facilities to organize their services in accordance with regional healthcare plans, which must set clear objectives for palliative care, thereby promoting structured and consistent service delivery across regions (Articles 2 and 4).  2016 law: The law requires that health professionals implement all means to ensure a dignified end of life with relief of suffering (Article 1) and that clinical decisions (e.g., limitation or withdrawal of treatment, sedation) follow documentation in the medical file, but does not establish detailed service or quality standards for palliative care delivery. | The law ensures quality through "multidisciplinary holistic care" standards (Article 2, 2016 update), palliative care teams in facilities (Article 5), and mandated quality reporting (Articles 4, 8). | The law ensures standards by defining palliative care as coordinated, multidisciplinary care addressing physical, psychological, and spiritual needs [Article 1], requiring state-organized training for staff [Article 1], and mandating a care logbook to document services [Article 1]. |
| 29. Financing | 1999 law: The following text is from Article 2. The text from Articles L. 712-3 and L. 712-3-1 of the Public Health Code provides both direct and indirect references to funding mechanisms for palliative care. Article L. 712-3 states that the regional healthcare organization scheme sets specific objectives for palliative care, to be implemented through contracts, implying allocated funding. Article L. 712-3-1 indicates that the annex to this scheme will outline the necessary resources for achieving these objectives, suggesting financial provisions for establishing palliative care units, mobile teams, and home hospitalization beds. Palliative care is part of the public healthcare system.  2016 law: Not addressed in the law text. | The law addresses financing by requiring recognized palliative care programs to be funded through social security, ensuring "accessibility, continuity, and quality" of services for patients (Article 3). Means that palliative care is publicly funded. | The palliative care law has nothing on funding mentioned. However, in the euthanasia law it is mentioned that "The operating expenses of the Commission are covered by the State budget." in Article 10. |
| 30. Additional aspects | 1999 law: The 1999 French law includes an additional relevant aspect in Article 10, which allows trained volunteers from approved associations to support end-of-life patients and their families. These volunteers can assist with non-medical support, provided they respect patient dignity, confidentiality, and personal beliefs, and they must operate under a formal agreement with healthcare establishments.  2016 law: The law also regulates advance directives being stored in a national register and their conditions of validity and confidentiality (Article 8), the role and designation of a trusted person (Article 9), and requires annual evaluation of the law’s implementation and palliative care policy by the Government reported to Parliament (Article 14). | As an additional aspect, even though the 2002 law (updated in 2016) does not mention education, it emphasizes to enhance the quality of palliative care and providing resources to address complex cases by specialized palliative care teams. (Article 7). | The law includes provisions for caregiver leave [Article 9] and amendments to other laws, such as the Social Security Code, to better integrate palliative care into the healthcare and social systems [Article 10]. |
| 31. Subsequent regulations | Following the 1999 French palliative care law, France enacted Kouchner law in 2002, the Leonetti Law of 2005 and the Claeys-Leonetti Law of 2016 to further develop end-of-life care. The 2005 Leonetti Law introduced the “right to die without suffering,” prohibited disproportionate treatments that artificially prolong life, and allowed patients to refuse treatment, with an emphasis on discontinuing futile care. This law also encouraged advance directives, though they were not yet legally binding. The Claeys-Leonetti Law of 2016 built on these principles by making advance directives legally binding and allowing for deep and continuous sedation until death for patients with a short life expectancy and severe, unmanageable suffering.   Subsequent to the 2016 law, France has adopted implementing decrees and regulatory measures alongside institutional developments and national palliative care plans aimed at improving access and organization of services; however, no new standalone law in palliative care has been enacted since the 2016 legal framework. | After the 2002 Belgian palliative care law, several regulations and amendments were introduced, including the 2016 update broadening the definition of palliative care, the establishment of Sciensano in 2018, and laws in 2017 and 2018 addressing patient identification and professional qualifications. Ministerial and royal decrees from 2003 to 2022 further supported the development of palliative care, including the creation of the Palliative Care Evaluation Unit and refining patient identification criteria. | The Palliative Care Law of Luxembourg is complemented by the Grand-Ducal Regulation of April 28, 2009, which specifies the procedures for accessing palliative care. In addition to the Grand-Ducal Regulation of April 28, 2009, which specifies procedures for accessing palliative care, the Palliative Care Law of Luxembourg has been complemented by the National Plan for End-of-Life and Palliative Care (2023-2026). |

# **Supplementary Table S3** Content Analyses of the National Palliative Care Legal Frameworks of Italy, Portugal and Germany.

| Indicators | Italy | Portugal | Germany |
| --- | --- | --- | --- |
| 1. WHO Region | EURO | EURO | EURO |
| 2. Income classification | HIC | HIC | HIC |
| 3. Level of palliative care development | Advanced | Advanced | Advanced |
| 4. Year of enactment | 2010 | 2012 and 2018 | 2015 |
| 5. Law title along with the reference | Legge 15 marzo 2010, n. 38: Disposizioni per garantire l'accesso alle cure palliative e alla terapia del dolore  <https://www.gazzettaufficiale.it/eli/gu/2010/03/19/65/sg/pdf> | **2012:** Lei n.º 52/2012, de 5 de setembro. Lei de Bases dos Cuidados Paliativos.   <https://diariodarepublica.pt/dr/detalhe/lei/52-2012-174841>  **2018:** Lei n.o 31/2018 de 18 de julho. Direitos das pessoas em contexto de doença avançada e em fim de vida.  <https://files.dre.pt/1s/2018/07/13700/0323803239.pdf> | Gesetz zur Verbesserung der Hospiz- und Palliativversorgung in Deutschland (Hospiz- und Palliativgesetz – HPG)  <https://www.bgbl.de/xaver/bgbl/start.xav?startbk=Bundesanzeiger_BGBl&jumpTo=bgbl115s2114.pdf#/text/bgbl115s2114.pdf?_ts=1774901319410> |
| 6. Law title in English | "Law of 15 March 2010, No. 38: Provisions to Ensure Access to Palliative Care and Pain Therapy" | **2012:** Law No. 52/2012 of September 5, the Fundamentals of Palliative Care.   **2018:** Law No. 31/2018 of July 18. Rights of People in the Context of Advanced Illness and End of Life | "Law to Improve Hospice and Palliative Care in Germany (Hospice and Palliative Care Act – HPG)" |
| 7. Word count | 4439 | 3779 (year 2012)  1517 (year 2018) | 1944 |
| 8. Structure | The law consists of a preamble and twelve articles, covering its purpose (Article 1), definitions (Article 2), government responsibilities (Article 3), national care networks (Article 5), professional training (Article 8), monitoring systems (Article 9), drug access (Article 10), reporting obligations (Article 11), and financial provisions (Article 12). | **2012:** The law structure covers the scope and definitions of palliative care (Article 2), principles of care (Article 4), rights of patients (Article 5) and families (Article 6), duties of patients and providers (Article 7), and the state's responsibilities (Article 8). It establishes the National Network of Palliative Care (Article 9) and addresses financing and implementation (Article 30).    **2018:** The law consists of 12 articles, organized into sections on general provisions (Articles 1-2), patients' and families' rights (Articles 3-9), therapeutic decisions (Articles 10-11), and final provisions (Article 12). | The law is organized into five articles amending the Social Code (statutory health and long-term care insurance) (Arts. 1-3), the Hospital Financing Act (hospital infrastructure funding) (Art. 4), and the Hospital Remuneration Act (hospital service payment system) (Art. 4a), concluding with its entry into force (Art. 5). |
| 9. Contents | The law ensures access to care (Article 1), defines service networks (Article 5), mandates pain assessment records (Article 7), requires professional training (Article 8), simplifies drug access (Article 10), monitors implementation (Article 9), and enforces reporting obligations (Article 11) | **2012:** The law egulates palliative care to alleviate suffering and improve quality of life (Article 3), ensuring patients' rights to care, information, and choice (Article 5), while supporting families' participation (Article 6). It regulates the state's responsibility through the National Health Service (SNS) and the National Network of Palliative Care (RNCP) (Article 8), with care provided by multidisciplinary teams.   **2018:** The law covers patients' rights to information, informed consent, and palliative care, including at home with caregiver support (Articles 3-7), palliative sedation (Article 8), and non-clinical rights like advance directives and healthcare proxies (Article 9). | The law integrates palliative care into statutory health insurance, expands outpatient services, enhances hospice funding and standards, and mandates counseling on palliative care and end-of-life planning (Art. 1, §§27, 37, 37b, 39a, 39b). |
| 10. Purpose | The law aims to ensure equitable access to palliative care and pain therapy, respecting "human dignity and autonomy" while addressing essential health needs and improving patients' quality of life (Article 1) | **2012** Law (Law No. 52/2012): Focuses on establishing the fundamentals of palliative care, including defining palliative care, setting up the National Network of Palliative Care (RNCP), and emphasizing the State's responsibility in delivering equitable and high-quality palliative care across the country(Portugal 2012).  **2018** Law (Law No. 31/2018): Extends the legal framework by emphasizing the rights of individuals in the context of advanced illness and end-of-life care. It is more patient-centered, detailing specific rights related to informed consent, refusal of treatment, and palliative sedation, while also addressing family rights and ethical considerations(Portugal 2018). | The law aims to improve end-of-life care, ensuring dignity and relief from suffering for terminally ill patients by integrating palliative care into health and long-term care systems (Art. 1, §§27, 37b, 39a) |
| 11. Objectives | The law aims to ensure access to care (Article 1), establish care networks (Article 5), simplify drug access (Article 10), and enforce monitoring and reporting obligations (Articles 9 and 11). | **2012** law aims to improve patient care by alleviating suffering (Article 3), ensure equitable access to palliative care for all (Article 9), and establish the National Network of Palliative Care. It also focuses on continuous professional training (Article 8) and effective coordination and monitoring by the Ministry of Health (Article 8).   **2018:** The law ensures access to palliative care (Article 6), informed consent (Articles 3-5), caregiver support (Article 7), palliative sedation (Article 8), and non-clinical rights like advance directives (Article 9). | The law set goals to ensure universal access to hospice and palliative care, strengthen outpatient and inpatient services through funding and quality standards, and promote individualized counseling and end-of-life planning support (Art. 1, §§27, 37, 39a, 39b). |
| 12. Principles | The law is guided by principles of respect for "human dignity and autonomy". | The **2012** law emphasizes respect for patient autonomy (Article 3) and holistic care (Article 6), guaranteeing patients' rights to information and participation (Article 5). It also affirms the state's responsibility to provide equitable palliative care through the National Health Service (SNS) with effective coordination (Article 8).   The **2018** law emphasizes the right to palliative care as the key principle (Articles 3,6,11). | The principle is to integrate palliative care into statutory health and long-term care systems and emphasizing patient-centered care and end-of-life decision-making support (Art. 1, §§27, 37, 39b). |
| 13. Palliative care right | The Article 1 clearly states that the law "protects the right of citizens to access palliative care and pain therapy". | The **2012** law emphasizes that the patient has the right to "Receive palliative care appropriate to the complexity of their situation and needs, including the prevention and relief of pain and other symptoms" (Article 5).   The **2018** law emphasizes the right to palliative care (Articles 3,6,11). | Explicitly recognized as an enforceable health care entitlement. The law states: ‘Medical treatment also includes palliative care for insured persons.’ By incorporating palliative care into the legal definition of medical treatment, the right to palliative care is legally binding within the statutory health insurance system.” |
| 14. Palliative care definition | The law defines palliative care as "therapeutic, diagnostic, and assistance interventions" for patients with progressive, incurable diseases, focusing on comprehensive care for both patients and their families (Article 2). | The **2012** law defines defines palliative care as "active, coordinated, and holistic care" provided to patients suffering from incurable or severe diseases in an advanced and progressive phase" (Article 2).   The **2018** law defines palliative care as care provided to individuals in advanced illness and end of life, including spiritual and religious support, as well as structured family support. It is delivered by a multidisciplinary team in various settings, focusing on symptom relief and comfort while avoiding therapeutic obstinacy (Articles 4-6). | No mentioned in the law text. |
| 15. Society | Article 4 mandates national information campaigns to raise public awareness about access to palliative care and pain therapy, aiming to overcome prejudice regarding the use of pain medications and to promote a culture of dignity and support for patients and families | The **2012** law promotes participation by ensuring patients can actively participate in care decisions (Article 5), involving families in decision-making and support (Article 6), and providing community access to palliative care through the National Network (Article 9).   The **2018** law promotes participation by enabling patients to make treatment decisions (Articles 3-5), involving families in decision-making (Article 10), supporting caregivers (Article 7), and providing access to ethics committees in case of discrepancy in decision-making (Article 11). | The law fosters participation by requiring individualized counseling, involving relatives in end-of-life planning (§39b), and encouraging collaboration between care facilities and hospice services to honor patient preferences (§39a). |
| 16. Governance | The law designates the Ministry of Health as the lead authority, responsible for policy setting, guideline development, and coordination with regions. It ensures access through service integration, monitoring, and designated national and regional funding (Articles 3, 5, 9, and 12). | The **2012** law designates the Ministry of Health to lead palliative care implementation, overseeing the National Network of Palliative Care (RNCP) (Article 8) and ensuring access and funding through specific budget allocations (Article 29).   The **2018** law establishes governance through the National Health Service (SNS) for centralized care (Article 6), and coordination between the Ministry of Health and Ministry of Labor to support caregivers (Article 7). | The law esignates statutory health insurance funds to implement and fund palliative care, provide counseling (§39b), monitor outpatient care (§37b), and establish quality standards and funding agreements for hospices (§39a). In addition the Federal Association of Health Insurance Funds reports to the Federal Ministry of Health for the first time by December 31, 2017, and every three years thereafter, on the development of specialized outpatient palliative care and the implementation of the guidelines issued by the Federal Joint Committee. |
| 17. Research | In the Article 9, it is mentioned that The Ministry particularly monitors "research activites" but that is all. | The **2012** law only mentions research as "Service differentiation may also occur based on the development research activities."   Not mentioned in the law text **2018**. | Not mentioned in the law text. |
| 18. Medicines | The law aims to simplify access to pain management drugs, including opioids, by streamlining prescription and dispensing procedures while ensuring proper monitoring (Article 10). | The **2012** law ensures access to essential medications in palliative care as defined by the World Health Organization (Article 31).   The **2018** law states that patients are entitled to chemical containment using appropriate medications, upon medical prescription." (Article 3). | Not explicitly mentioned in the law text. Only "supply of medications" is mentioned. |
| 19. Undergraduate training | Undergraduate training is not mentioned. | The **2012** law does not mention incorporating palliative care training into undergraduate educational programs.   The **2018** law does not mention incorporating palliative care training into undergraduate educational programs. | Not mentioned in the law text. |
| 20. Continuing education | "Continued Training and Update for Volunteers and Healthcare Professionals" is mentioned in the law text. | The **2012** law includes provisions for continuing education in palliative care for healthcare professionals.   Not mentioned in the law text of **2018**. | Not mentioned in the law text. |
| 21. Specialization | Specialized health care professionals are mentioned but not specialization. | In the **2012** it is mentioned in Article 27 that "The human resources policy for the Palliative Care Units and Teams is based on quality standards, which require specialized training, according to the recommended levels of differentiation."   In the **2018** law specialization is not mentioned. | Not mentioned in the law text. |
| 22. Integration of services | The law integrates primary and specialized care through national and regional networks. Pediatric palliative care is also highlighted. | The **2012 l**aw strengthens the National Health System (SNS) by integrating primary and specialized palliative care through the National Network of Palliative Care (RNCP) (Article 9).   Integration of services is not explicitly mentioned in the **2018** law text. | The law does not address the integration between primary and specialized care services. It focuses on the inclusion of palliative care in statutory health insurance (§27). |
| 23. Specialized services | Specialized palliative care teams are mentioned. | In article 16 the **2012** law addresses "The Palliative Care Unit is a specialized service that treats patients who require differentiated and multidisciplinary palliative care, particularly in complex acute clinical situations."   In the **2018** law it is mentioned that individuals who are facing uncontrolled suffering are subject to regular clinical monitoring by teams of professionals duly accredited in providing palliative care. (Article 8). | Not mentioned in the law text. |
| 24. Palliative sedation | Not mentioned in the law text. | Palliative sedation is not mentioned in the **2012** law.   In the **2018** law text it is mentioned that palliative sedation may be used for patients with uncontrolled suffering and a short life expectancy (Article 8). The sedation must be administered with appropriately titrated sedative medications. | Not mentioned in the law text. |
| 25. Advance care planning | The law does not mention advance care planning or decision-making for neither for patients with capacity nor with incapacity | The **2012** law guarantees patients the right to participate in decisions about their care, including the ability to express their wishes regarding treatment (Article 5). In addition, it is mentioned that "Palliative care intervention is based on an individual palliative care plan, which is developed and organized by the interdisciplinary team for each patient." ACP is not specifically mentioned.   In the **2018** law the text word "ACP" is not mentioned. However, the law allows patients to create advance directives and appoint a healthcare proxy for decision-making if incapacitated (Article 9). If no directive exists, the healthcare team consults the family to make decisions in the patient's best interest (Article 10). | It is mentioned in the law text that Health insurance funds inform their insured persons in general terms about options for personal end-of-life planning, especially regarding advance directives, durable powers of attorney, and guardianship directives. |
| 26. Adaptation of therapeutic effort | It highlights the integration of medical, psychological, and social support services through multidisciplinary teams but does not explicitly mention adapting care based on patient preferences or desires (Article 1 and Article 2) | The **2012** law allows for personalized palliative care by adapting interventions to patient needs and preferences and ensuring their involvement in care decisions (Article 5, Article 3).   The **2018** law allows patients to actively participate in their treatment plans, specifying desired measures and refusing those that don't align with their preferences (Article 3) | The law supports the adaptation of interventions to patient needs and preferences by mandating individualized counseling (§39b). In addition, it is mentioned in Article 1 that residents of inpatient care facilities may transfer to an inpatient hospice, considering the legitimate wishes of the residents." |
| 27. Requests for assisted death | Not mentioned in the law text. | Not mentioned in the **2012** law text.   Not mentioned in the **2018** law text. | Not mentioned in the law text. |
| 28. Standards | The law ensures standards and quality through national care networks (Article 5), mandatory pain assessment records (Article 7), standardized professional training (Article 8), and monitoring by the Ministry of Health (Article 9) | The **2012** law ensures quality care by requiring multidisciplinary teams (Article 6), setting quality standards monitored by the Ministry of Health (Article 8), integrating services through the National Network of Palliative Care (Article 9), and mandating ongoing professional training (Article 8).   The **2018** law The law ensures quality palliative care through accredited multidisciplinary teams (Article 6), patient-centered care (Articles 3-5, 6), professional training (Article 6), and ethical standards like informed consent and avoiding therapeutic obstinacy (Articles 4-5). | The law ensures quality through nationwide standards for hospice services (§39a), structured counseling requirements (§39b), and reporting obligations to monitor specialized outpatient palliative care (§37b). |
| 29. Financing | The law allocates €1,650,000 for 2010, €1,300,000 for 2011, €300,000 for 2012, and €150,000 annually from 2013 onward. Additionally, at least €100 million annually from the National Health Fund is designated for implementation based on the Minister of Health’s proposal (Article 12). Palliative care is incorporated in the National Health Plan/Service. | The **2012** law ensures funding for palliative care through a specific budget allocation from the Ministry of Health (Article 29) and regulated financing for services (Article 30).   Not mentioned in the law text of **2018.** | The law mandates funding through statutory health insurance, covering inpatient hospice costs (§39a, Paragraph 1) and supporting outpatient hospice services for personnel and material expenses (§39a, Paragraph 2). It also funds specialized outpatient palliative care with regular cost monitoring (§37b, Paragraph 4) and counseling services, including end-of-life planning (§39b). |
| 30. Additional aspects | The law includes provisions on mandatory pain assessment records (Article 7), public awareness campaigns (Article 4), and simplified pain medication management (Article 10) | The **2012** law covers family and caregiver support (Article 6), continuity of care (Article 9), psychological support (Article 8), and coordination between healthcare services (Article 10).   The **2018** law covers caregiver support (Article 7), social benefits for patients and families (Article 9), and access to ethics committees for resolving disputes (Article 11). | The law introduces requirements for health insurance funds to provide detailed reporting on specialized outpatient palliative care (§37b) and ensure collaboration between care facilities and hospice services (§39a). It also mandates the development of nationwide quality standards for hospice services to ensure consistency in care (§39a). |
| 31. Subsequent regulations | Since Law No. 38 of March 15, 2010, Italy has issued measures like accreditation standards (2020-2021), Decree No. 77/2022 on territorial care models, and Law No. 197/2022 mandating regional palliative care plans. | After the 2012 law, an updated/modified version was published in 2018. | In 2018, Germany legally introduced reimbursement for advance care planning (ACP according to § 132 g SGB V) in nursing homes. This initiative aims to improve end-of-life care by facilitating discussions about residents' preferences and ensuring that their wishes are respected. |

# **Supplementary Table S4** Content Analyses of the National Palliative Care Legal Frameworks of Armenia, Albania and Austria.

| Indicators | Armenia | Albania | Austria |
| --- | --- | --- | --- |
| 1. WHO Region | EURO | EURO | EURO |
| 2. Income classification | UMIC | UMIC | HIC |
| 3. Level of palliative care development | Progressing | Emerging | Advanced |
| 4. Year of enactment | 2017 | 2014 | 2022 |
| 5. Law title along with the reference | Պալիատիվ բժշկական օգնություն և ծառայությունների տրամադրման ստանդարտի հաստատման մասին  <http://www.arlis.am/DocumentView.aspx?DocID=132500> | LIGJ Nr. 138/2014 PËR KUJDESIN PALIATIV NË REPUBLIKËN E SHQIPËRISË  <https://qbz.gov.al/eli/ligj/2014/10/16/138> | Bundesgesetz über die Einrichtung eines Hospiz- und Palliativfonds und über die Gewährung von Zweckzuschüssen an die Länder zur finanziellen Unterstützung der Hospiz- und Palliativversorgung ab dem Jahr 2022 (Hospiz- und Palliativfondsgesetz – HosPalFG)  <https://www.ris.bka.gv.at/eli/bgbl/I/2022/29> |
| 6. Law title in English | On Approval of the Standard for the Provision of Palliative Medical Care and Services | Law No. 138/2014 on Palliative Care in the Republic of Albania | Federal Act on the Establishment of a Hospice and Palliative Care Fund (Hospice and Palliative Care Fund Act – HosPalFG), BGBl. I No. 29/2022 |
| 7. Word count | 3309 | 3163 | 4473 |
| 8. Structure | The Armenian palliative care law is organized into 8 sections covering general provisions, principles of care, methods of implementation (outpatient, mobile, inpatient, hospice), beneficiaries, care organization in medical institutions, mobile services, and pediatric palliative care. | The law is organized into six chapters: general provisions, palliative care services, service provision, access to medicines, funding, and final provisions, including sanctions and subordinate acts. | The law is structured into 18 articles, covering the establishment of the Hospice and Palliative Fund, financial provisions, definitions, subsidy conditions, planning and reporting requirements, enforcement, and regulatory authority. |
| 9. Contents | The Armenian law regulates palliative care for life-threatening or life-limiting illnesses (Section 5), emphasizing pain control and affirming life while prohibiting hastening death (Section 12). Care is provided by a multidisciplinary team (Section 6) and includes outpatient, mobile, inpatient, and hospice services (Section 14), for patients with incurable conditions (Section 15). | The law regulates palliative care services, patient rights, pain management, and access to essential medicines like opioids (Articles 3, 8, 22-23). It also covers funding mechanisms and sanctions for non-compliance (Articles 24-27). | The law covers the establishment of the Hospice and Palliative Fund, financial support, subsidy conditions, quality management, planning, data collection, reporting, and enforcement. |
| 10. Purpose | In the law text (Section 1) it is mentioned that "This standard defines the basic principles of providing palliative medical care and services to the population and the relationships related to the organization of palliative medical care." | The law aims to protect citizens' right to palliative care (Article 1), ensuring dignity, quality of life, and equitable access to services and pain management (Article 5, Articles 22-23), addressing the need for specialized care in the context of public health and social responsibility. | The law establishes a federal Hospice and Palliative Care Fund to financially support the Länder in implementing a nationwide, needs-based, and uniformly organized hospice and palliative care system, with the aim of improving the availability, accessibility, and affordability of services for palliative patients and their relatives. |
| 11. Objectives | The Armenian law on palliative care aims to alleviate pain and symptoms, ensure access to multidisciplinary care, and expand palliative care services through outpatient, mobile, inpatient, and hospice models (Sections 12, 14). It also focuses on providing care for patients with incurable conditions, including malignant neoplasms and organ failure (Section 15), and improving the training and qualifications of healthcare workers in palliative care (Section 23,29). | The law aims to ensure access to palliative care, effective pain management (Articles 1, 22-23), establish multidisciplinary teams (Article 3), regulate services (Article 8), protect patient rights (Article 5), secure funding (Articles 24-25), and enforce compliance (Article 27). | The law's specific objectives are to fund and expand specialized hospice and palliative care services, ensure quality management, set uniform tariffs, support training, and improve accessibility and coverage of care across Austria. |
| 12. Principles | The ethical foundations of the Armenian palliative care law are centered on respecting the dignity of patients, affirming life while accepting death as a natural process, and prioritizing pain relief and symptom management (Section 12). | The ethical, legal, and conceptual foundations of the law are based on the principles of human dignity, autonomy, and the right to care. | The law is based on principles of accessibility, quality, equity, and integration of services, aiming to provide comprehensive, patient-centered, and needs-based palliative care throughout Austria. |
| 13. Palliative care right | Not explicitly addressed in the law text. Palliative care provision is mentioned as follows: "Palliative medical care and services (hereinafter referred to as Palliative Medical Care) are provided to any person with a life-threatening or ongoing and/or life-limiting illness." | The law explicitly mentions the right to receive palliative care in Article 1, with further reinforcement in Articles 5 and 8, ensuring access to care tailored to patients' needs. | Not explicitly recognized. The law does not establish an individual right to palliative care but creates a federal funding and governance mechanism to support the development and expansion of hospice and palliative care services at the regional level. |
| 14. Palliative care definition | Palliative medical care and services (hereinafter referred to as Palliative Medical Care) are provided to any person with a life-threatening or ongoing and/or life-limiting illness, and aim to alleviate the patient's pain and physical,socio-psychological, spiritual and other problems associated with the illness through early detection, assessment andimplementation of necessary interventions (Section 1). Palliative care is provided by a multidisciplinary team that includes a doctor, a nurse, and other specialists, such as a psychologist and a social worker, as needed. Patients, their families, and the community may also be involved in the work of the multidisciplinary team (Section 1). | The law defines "Palliative care is the totality of diagnostic, therapeutic, and psychosocial interventions aimed at both the patient and their family when the disease and the patient, due to the evolution and prognosis of the disease, no longer respond positively to the interventions provided for its treatment." | The law defines palliative care as specialized care provided to individuals with life-limiting or terminal conditions, focusing on alleviating symptoms, improving quality of life, and offering support to patients and their families, including both adults and pediatric patients (Article 2). |
| 15. Society | The law encourages family involvement in care planning (Section 25) and promotes community collaboration, including with social services and religious groups (Sections 23, 40). | The law promotes participation by guaranteeing patients' rights to be informed and involved in decisions about their care (Article 5). It also includes families in the care/communication (Article 17) and encourages community involvement through volunteer services (Article 15). | Decision-making is not mentioned but the law recognizes the role of volunteers, including both non-volunteer and volunteer hospice companions. |
| 16. Governance | It is only mentioned that "The scope of palliative care provided at the hospital level is the organization of palliative care cooperation with territorial bodies or organizations providing social services in accordance with the Resolution of the Government of the Republic of Armenia." | The law establishes the Ministry of Health as the leader in developing policies, ensuring access to services (Article 6) and overseeing funding through the health insurance scheme and state investments (Articles 24-25), ensuring proper implementation and regulation of palliative care. | By granting purpose subsidies from the Hospice and Palliative Fund, the federal government supports the states in implementing a nationwide, needs-based, and uniformly organized hospice and palliative care system (Article 1). Goverment also ovesights the development of palliative care. |
| 17. Research | Not explicity mentioned but it is mentioend that "Provide palliative care from the early stages of the disease in conjunction with life-prolonging medical care(chemotherapy, radiotherapy, etc.), conducting the research necessary to understand and manage severe clinical complications." | In article 3, under hospice, it is mentioned that the hospice services may also be accompanied by research in the field of palliative care. Nothing else is mentioned about research. | The law does not explicitly address provisions for research related to palliative care. However, it implicitly supports the development of evidence-based practices through its emphasis on quality management, data collection, and the establishment of minimum standards for care. This research-oriented approach could help improve the delivery of palliative care services, though it is not specifically outlined in the law (Article 6, Article 10 |
| 18. Medicines | The law ensures access to essential medications, including opioid painkillers, with some medications reimbursed for eligible patients (Section 5, Section 24). | The law ensures access to essential medications for pain and symptom management, including opioid analgesics (Articles 22-23), and mandates pharmacies to maintain a supply and regulate their prescription and distribution. | Not mentioned in the law text. |
| 19. Undergraduate education | Not mentioned in the law text. | Not mentioned in the law text. | Not mentioned in the law text. |
| 20. Continuing education | The law emphasizes that healthcare workers involved in palliative care must receive ongoing training, particularly within the last five years, to ensure they are equipped with current knowledge and skills (Section 19). | The law requires accredited training programs by the National Continuing Education Center (Article 21) but does not specifically mention continuing education. | The law places a strong emphasis on the continuing education of both volunteer and non-volunteer personnel involved in palliative care. It outlines that purpose subsidies can be used for the financing of ongoing training, further education, and professional development in palliative care (Articles 4 and 6). |
| 21. Specialization | Th law promotes specialization in palliative care by requiring healthcare professionals involved in palliative care, including physicians, to receive specific training in the field. It mandates that doctors working in palliative care settings have undergone relevant training within the past five years (Section 19). | The law promotes specialization in palliative care through Article 21, which mandates the creation of a short-term specialization program in palliative care for healthcare professionals. | The law emphasizes in Article 13 that 'Quality criteria and indicators aim to achieve uniform minimum care standards for specialized hospice and palliative care throughout Austria. These include the required characteristics regarding staffing and qualifications as well as the technical and spatial facilities of the service offerings.' |
| 22. Integration of services | The law integrates primary and specialized care by providing palliative services through various levels, including primary healthcare, mobile services, and hospices, with a multidisciplinary team approach (Sections 14, 16, 28). Pediatric PC services is also addressed in the law text. | The integration is not speficially mentioned but in Article it is mentioned that "The establishment and operation of palliative care services in public healthcare institutions are determined by the order of the Minister of Health." In addition, the Ministry of Health is responsible for developing health policies and monitoring health care services to ensure that palliative care. In addition, bed hospice service is included as an integrated service. | The law emphasizes the integration of specialized hospice and palliative care services within the broader healthcare system. This includes the coordination of mobile palliative teams, inpatient hospices, and other care facilities (Article 2, 4, 6). |
| 23. Specialized services | It is mentioned that "In case of need for palliative specialized hospital medical care and services, the patient is recommended toreceive the appropriate medical care in inpatient conditions." In addition, it is mentioned that "the provision of services within the framework of other types of medical care and services provided to the patient in connection with the given disease is completely discontinued, a registration of the transition to palliative care is made in the patient's medical records, and the patient is monitored by a palliative care specialist." | The law provides specialized palliative care for complex patients through bed hospice services (Article 12) and hospital-based care (Article 13), delivered by a multidisciplinary team in specialized settings. | The law provides for specialized palliative care services, including mobile palliative teams, hospice teams, pediatric palliative care teams, and inpatient hospice facilities. Article 2, 4. |
| 24. Palliative sedation | Not mentioned in the law text. | Not mentioned in the law text. | Not mentioned in the law text. |
| 25. Advance care planning | It is mentioned that "The multidisciplinary team of the palliative care and service office develops and regularly reviews anindividualized palliative care and care plan (hereinafter referred to as the plan) with the patient and his family." | The law ensures that patients can make informed choices about their treatment (Article 5). Decision-making for patients in cases of incapacity is not mentioned in the law text. | Advance care planning or decision-making are not mentioned. The only thing mentioned is 'Conducting preventive and informational discussions, particularly the VSD preventive dialogue®.' which is not specifically about ACP. |
| 26. Adaptation of therapeutic effort | The law allows for the adaptation of interventions by requiring individualized care plans (25) | The law allows adaptation of palliative interventions based on patient preferences (Article 5) and ensures care respects dignity and autonomy (Article 4). | The law emphasizes ensuring that palliative care services meet the needs of patients and are tailored to their specific conditions. |
| 27. Requests for assisted death | Not mentioned in the law text. | Not mentioned in the law text. | Not mentioned in the law text. However, Austria has a separate law that regulates assisted suicide (Sterbeverfügungsgesetz (StVfG)), enacted in 2022. |
| 28. Standards | The law ensures quality by requiring a multidisciplinary team (Section 6), standardized care methods (Section 14), ongoing healthcare worker training (Section 29), and regular updates to care plans (Section 25). | The law ensures quality and consistency in palliative care through principles of dignity and pain management (Article 4), regulation of care services (Article 8), and medical and nursing standards (Article 17). It also enforces sanctions for non-compliance (Article 27). | These standards include requirements for staffing levels, qualifications, and the technical and physical infrastructure of care services. The aim is to achieve uniform minimum care standards throughout the country, ensuring that all palliative care services meet these standards and are accessible to those in need (Article 2, Article 6) |
| 29. Financing | The law allows palliative care to be provided through state-guaranteed free services or on a paid basis, with costs set by providers. | The law outlines funding in Article 24, covering palliative care through health insurance and the State Budget, and Article 25 provides funding for public services, with support for private initiatives through agreements with the Ministry of Health. | The law outlines the financial provisions for hospice and palliative care, including the creation of the Hospice and Palliative Fund, which provides purpose subsidies to the states. These subsidies are intended to support the establishment and expansion of palliative care services, including mobile palliative teams, hospice teams, and inpatient facilities. The law ensures that financial support is available to maintain and enhance the accessibility and quality of palliative care services across Austria (Article 3, Article 4) |
| 30. Additional aspects | The law also covers the organization of palliative care in primary settings and hospices (Sections 16, 28), mandates the involvement of specialists like social workers and psychologists (Section 6), includes provisions for pediatric care (Section 40), and emphasizes record-keeping and reporting (Section 23). | The law also covers volunteer involvement (Article 15), social support for terminally ill patients (Article 26), and ensures confidentiality of patient information. | It includes references to provisions of other federal laws, indicating that those provisions apply in their currently valid version (Article 17). |
| 31. Subsequent regulations | The law is complemented by the Order of the Minister of Health No. 45-N (2017), which sets standards for palliative care, and the Resolution No. 642-N (2019), which addresses medication reimbursement for eligible patients. | Not exists. | Austria has a separate law that regulates assisted suicide (Sterbeverfügungsgesetz (StVfG)), enacted in 2022. |

# **Supplementary Table S5** Content Analyses of the National Palliative Care Legal Frameworks of Greece, Romania and Israel.

| Indicators | Greece | Romania | Israel |
| --- | --- | --- | --- |
| 1. WHO Region | EURO | EURO | EURO |
| 2. Income classification | HIC | UMIC | HIC |
| 3. Level of palliative care development | Progressing | Established | Established |
| 4. Year of enactment | 2022 | 2018 | 2005 |
| 5. Law title along with the reference | ΝΟΜΟΣ ΥΠ’ ΑΡΙΘΜ. 5007 Ολοκληρωμένο Σύστημα Παροχής Ανακουφιστικής Φροντίδας – Ρυθμίσεις για την αντιμετώπιση της πανδημίας του κορωνοϊού COVID-19 και την προστασία της δημόσιας υγείας και άλλες επείγουσες ρυθμίσεις  <https://20.95.103.179/api/DownloadFeksApi/?fek_pdf=20220100241> | ORDIN nr. 253 din 23 februarie 2018 privind aprobarea Regulamentului de organizare, funcționare și autorizare a serviciilor de îngrijiri paliative  <https://legislatie.just.ro/Public/FormaPrintabila/00000G2GJZI4UZYQZ980E8LXY72L9I09> | The Dying Patient Law, 2005.  <https://www.medethics.org.il/wp-content/uploads/2021/12/28_DyingPatientLaw_366-404.pdf> |
| 6. Law title in English | LAW No. 5007 Integrated System for the Provision of Palliative Care – Provisions for addressing the COVID-19 pandemic and protecting public health, and other urgent regulations. | Order No. 253 of 23 February 2018 on the approval of the Regulation for the organisation, functioning and authorisation of palliative care services. | The Dying Patient Law, 2005. |
| 7. Word count | 10496 | 3459 | 9441 |
| 8. Structure | Chapter B of Law 5007/2022, which constitutes the core legal framework for palliative care in Greece, includes Articles 1 to 15. It defines the purpose, scope, and key terms, as well as the guiding principles and objectives of the palliative care system. It regulates the creation of the National Action Plan, the care structures, the National Registry of Patients, and the National Palliative Care Committee, including its composition and responsibilities. It also addresses the reimbursement of services, professional training, acceptance of financial donations, and includes transitional and regulatory provisions | The text is organized into 24 consecutively numbered articles that set out definitions, levels of care, requirements, authorisation procedures, control mechanisms, and final provisions.  It also includes 15 annexes, which form an integral part of the regulation and mainly cover concepts, organisational arrangements, authorised structures, staffing requirements, service standards, operational procedures, and clinical documentation templates | Seven chapters: (1) purpose & fundamental principle; (2) definitions; (3) presumptions (will to live, competence); (4) treatment of the terminally ill—Arts. A–G (general; extraordinary treatment; withholding/non-renewal; emergencies; prohibited acts; palliative care; minors/protected persons); (5) advance directives & proxies + national database; (6) institutional/national committees; (7) miscellaneous (liability, conscience transfer, confidentiality, regulations, entry into force). |
| 9. Contents | The law establishes a “comprehensive and integrated system for the provision of palliative care services to patients suffering from life-threatening or life-limiting illnesses.” It defines palliative care, its principles, and the structures responsible for its delivery. It creates a National Palliative Care Committee, a National Registry of Patients, and sets out the National Action Plan for the Development of Palliative Care. It also regulates reimbursement of services, training of professionals, telemedicine objective, and the possibility of receiving donations. | The regulation defines palliative care concepts, levels of intervention, and service standards, including paediatric care. It sets authorisation and reporting requirements, integrates services into the national health system via a national plan, and provides annexes on principles, beneficiaries, staffing, equipment, operational models, and clinical documentation, ensuring consistent quality and oversight nationwide. | Regulates medical treatment of the terminally ill patient; defines categories (“terminally ill” ≤6 months; “end-stage” ≤2 weeks); sets presumptions (wish to live, competence) and procedures to withhold/not initiate treatments; creates advance directives and proxy (valid for 5 years with a national database); establishes institutional committees and a national committee to resolve disputes; guarantees palliative care to relieve suffering (including support to the family) and prohibits euthanasia and assisted suicide. |
| 10. Purpose | The aim is to ensure equal and uninterrupted access to high-quality palliative care throughout the country. | The regulation aims to ensure that all patients requiring palliative care receive timely, appropriate, and high-quality services, regardless of diagnosis or age, within an organised national framework. It seeks to standardise definitions, service levels, and operational requirements, integrating palliative care into the health system to safeguard dignity, alleviate suffering, and support families. | Purpose (Art. 1): “to regulate the medical treatment of the terminally ill patient,” balancing the sanctity of life, autonomy and quality of life; based on “the values of the State of Israel and principles of morals, ethics and religion”. |
| 11. Objectives | The law aims to:  Ensure continuous and equitable access to high-quality palliative care across the country.  Integrate palliative care into all levels of health service delivery.  Create a standardized and coordinated national system through the National Action Plan.  Establish care structures and interdisciplinary teams based on defined standards.  Support the training of professionals and promote public awareness.  Monitor care provision through a centralized patient registry.  Promote effective governance via a National Committee for Palliative Care. Develop special treatment and hospitalization structures for patients that strengthen the possibility of participation of their family and familiar environment during the stages of their treatment and hospitalization. Develop modern electronic telemedicine methods for the advisory support of patientsand their families. | Defines scope, principles, and levels of palliative care; sets uniform standards; regulates authorisation and quality control; integrates services into the national health system via a coordinated plan; and includes specific provisions for paediatric care. | Set clear rules for end‑of‑life decisions (presumptions, consent, advance directives and representatives, documentation), criteria on non‑prolongation and extraordinary treatments, a committee structure and reporting obligations; ensure relief of the patient’s suffering and support to the family. |
| 12. Principles | The law is based on key principles that include:  Respect for the dignity, autonomy, and wishes of patients.  Holistic, person-centered care addressing physical, psychological, social, and spiritual suffering.  Equitable access to treatment and hospitalization of patients in need of palliative care, so as to ensure the possibility of providing health care at their home or in structures suitable for them (implicitly: regardless of age, diagnosis, or place of residence)  Interdisciplinary collaboration and continuity of care.  Integration of palliative care into the national health system as a recognized health service.  Commitment to quality, safety, and accountability in care provision. | The regulation is guided by principles set out in Annex 1, including respect for human dignity and patient rights, equity in access, a patient- and family-centred approach, continuity and quality of care, interdisciplinary teamwork, and the integration of physical, psychological, social, and spiritual dimensions to alleviate suffering and support autonomy. | Principle (Art. 2): when prescribing treatment to the terminally ill patient, his medical condition, his will and his degree of suffering are the exclusive considerations. |
| 13. Palliative care right | The law does not explicitly establish an individual, enforceable ‘right to palliative care’. However, it establishes an institutional framework for an integrated palliative care system aimed at improving quality of life and explicitly commits to improving conditions of access to care for patients needing palliative care (including enabling home-based care or care in suitable structures). | The regulation does not explicitly frame palliative care as a legal right but mandates that “all patients meeting the eligibility criteria must have access to palliative care services” at the appropriate level, ensuring equity, continuity, and quality. This effectively implies a guaranteed right through enforceable service provision standards. | Implied, not explicit. The law does not declare a subjective right to palliative care, but Article 23 obliges the responsible physician to “do everything possible to alleviate the pain and suffering” of a terminally ill patient (including pharmacological, psychological, nursing, environmental measures and family support), which effectively implies such a right. |
| 14. Palliative care definition | Palliative care is defined as “a holistic approach aimed at improving the quality of life of patients of all ages suffering from life-threatening or life-limiting illnesses, as well as their families, through the prevention and relief of suffering.” It includes “the early identification, correct assessment, and treatment of pain and other physical, psychosocial, and spiritual problems,” and it is provided by “interdisciplinary and appropriately trained teams, in collaboration with the patient’s environment and caregivers.” | The regulation defines palliative care as “the active total care of patients whose disease is not responsive to curative treatment, aiming to control pain and other symptoms, and to address psychological, social, and spiritual problems in order to achieve the best possible quality of life for patients and their families. It also includes early identification, assessment and treatment of pain and other problems, and extends support to the family during the patient’s illness and in bereavement. | The Law does not define “palliative care” as a term; Article 23 requires the responsible physician to “make sure that everything possible is done in order to alleviate the pain and suffering of the patient,” including pharmacological, psychological, nursing and environmental measures, and support to the family’s wellbeing. |
| 15. Society | The law promotes public awareness and social participation by encouraging civil society organizations to contribute to service provision and support, although public awareness is not explicitly included in the text. It also recognizes the role of trained volunteers as part of interdisciplinary palliative care teams. The legal framework encourages cooperation with patients’ families and familiar environment with the aim of empowering individuals, families, and communities in the care process, although patient associations and community actors are not explicitly addressed in the law text. | The regulation recognises the family as the unit of care and promotes its active involvement in decision-making, particularly in paediatric palliative care. The law encourages to organize information and awareness campaigns for the population regarding palliative care. In addition, volunteer services are highlighted as part of the social services in the law text. However, the law does not establish formal mechanisms for broader community participation in policy development. | Recognises and empowers the person and their close network: defines a “close person,” provides for notification to the proxy/family, respects advance directives and representation, and requires conditions that reduce the burden on close persons during the stay (Art. 23[b]). Volunteer services are not addressed. |
| 16. Governance | The law establishes a clear governance structure by creating the National Committee for the Development of Palliative Care, responsible for planning, coordination, monitoring, and evaluation of services nationwide. The Committee is appointed by the Ministry of Health and includes representatives from relevant ministries, professional associations, academia, and civil society. It is tasked with drafting the National Action Plan and issuing opinions on regulations and policies related to palliative care. The law also mandates the creation of a National Registry of Palliative Care Patients to support oversight and planning. | The regulation assigns the Ministry of Health overall responsibility for implementing, authorising, and monitoring palliative care services (Art. 22–24). It establishes a national framework through the National Plan for the coordinated development of Palliative care (Art. 8), sets provider accreditation procedures, mandates annual reporting, and defines quality control mechanisms to ensure equitable access and compliance with national standards. | Creates Institutional Committees and a National Committee with multidisciplinary composition (medicine, nursing, social work/psychology, ethics, legal, religious authority) and powers to resolve disputes and appeals (Arts. 45–53). The Minister implements the Law (Art. 61) and may amend annexes/forms (Art. 62). |
| 17. Research | The law enables palliative care structures (DAFA) operating within hospitals/private clinics to collaborate with university centers of excellence via programmatic contracts aimed at developing innovative palliative care protocols and conducting other research activities. In addition, the National Palliative Care Patient Registry is explicitly intended to support the formulation and development of research and innovative palliative care protocols and to interface with corresponding European registries through annual transmission of anonymized data for cross-country comparison and exchange. | Not addressed in the law text. | The law mandates the Ministry to establish reporting duties on implementation ‘for purposes of supervision and research,’ and permits publication of such information without identifying details (Sec. 60(a)(6)). This explicitly links reporting to “research” and regulates publication of non-identifiable information. |
| 18. Medicines | The law does not include specific provisions regarding access to essential palliative care medicines, including opioids. It does not mention prescribing practices, availability, regulatory mechanisms, or supply chain considerations for medicines used in palliative care. | Regarding medications, the law defines specialized palliative care as a care provided by providers authorized in prescribing, applying and monitoring appropriate pharmacological treatment. Annex 13 specifies the minimum mandatory equipment for the dispatcher site of home palliative care providers, including basic medical consumables and infusion-related materials (e.g., needles, syringes, infusion solutions and infusion sets). However, no specific regulatory provisions on essential medicines are included. | No specific content identified on access to essential medicines; Article 23 authorises relieving suffering even with a reasonable risk of death (proportional use of analgesics, etc.). |
| 19. Undergraduate education | Undergraduate education is not explicitly addressed in the law. The legal instrument does not contain provisions mandating palliative care content in undergraduate curricula (Article 15 is a repeal of a provision in Law 3106/2003: Nursing Care and Palliative Care Hospitals). | No content identified | No content identified. |
| 20. Continuing education | The law does not establish a general continuing professional development (CPD) obligation, or ongoing in-service education mandate. It instead regulates a defined training programme and certification mechanism linked to workforce qualification for palliative care provision (Article 11) | The regulation requires that personnel working in palliative care meet national qualification standards and be trained according to national protocols, including paediatric care, as part of the provider authorisation criteria (Annex 4). | No content identified. |
| 21. Specialization | The law recognizes the need for a medical specialization in palliative care for both adults and children, but indicates that it has not yet been formally established. As a transitional measure, Article 14 sets out detailed qualification criteria for physicians, nurses, and other professionals working in Palliative Care Structures. These include at least two years of clinical experience in the field and either a recognized postgraduate degree or a certified training program of at least 400 hours (200 theoretical, 200 clinical), as described in Article 11. Until the specialization is officially created, these standards apply for staffing purposes. | Authorised palliative care services must employ professionals with specific training and competencies in palliative care, in accordance with national requirements for each professional category (Annex 4). | No content identified. |
| 22. Integration of services | “Palliative Care Structures shall be integrated into the National Health System and operate in coordination with primary, secondary and tertiary healthcare services, ensuring continuity of care.” | The regulation integrates palliative care into the national health system through a three-tier model (education/self-care support, basic palliative care, specialised palliative care), ensuring continuity between primary and specialised services. It mandates coordination across hospitals, hospices, home care, and community services, including paediatric care, within the National Plan for coordinated development. | Not explicitly addressed. The Law regulates end-of-life decision-making and obliges symptom relief within existing care settings, but it does not mandate system-level integration across primary, community, and specialized palliative services (service design is left to existing arrangements and later regulations). |
| 23. Specialized services | Artículo 11 – “Palliative Care Structures include: a) Home palliative care services; b) Hospices; c) Hospital palliative care units; d) Pediatric palliative care units.” Artículo 14 – Until the medical specialization in palliative care is formally established, staffing of Palliative Care Structures shall follow the qualification criteria described herein…” | Specialised palliative care is provided in dedicated hospital units, hospices, and home care teams for complex cases requiring multidisciplinary expertise. The regulation specifies staffing standards, infrastructure, and operational procedures for these services, including 24/7 availability, paediatric specialisation, and compliance with national protocols for symptom management and holistic support. | No specific provision to design/develop specialised palliative care services integrated into the national health system; the Law regulates decision‑making and relief of suffering within existing arrangements. |
| 24. Palliative sedation | Not mentioned in the law text. | No content identified | Not expressly named or defined. Article 23 obliges clinicians to “do everything possible” to relieve pain and suffering, including by medication even with a reasonable risk of death. |
| 25. Advance care planning | Not mentioned in the law text. | The regulation does not explicitly address advance care planning or advance directives. It indirectly encourages patient and family participation in care decisions, particularly in paediatric palliative care, but without formal procedures or legal safeguards. | Full advance care planning framework: 5-year renewable advance directives and proxy appointment recorded in a national database. In incapacity, decisions follow a cascade (directives → proxy → committee/physician), with required consultation, notification, documentation, and periodic review. The role of a power of attorney is also highlighted. |
| 26. Adaptation of therapeutic effort | The law refers to patient-centred care and quality of life (Article 4, Part B) but does not regulate withholding or withdrawing treatment. | The regulation promotes patient- and family-centred, individualised care in line with national protocols, allowing adaptation of interventions to needs and preferences, but without explicit legal procedures for limiting or withdrawing treatment. | Care is tailored to the patient’s medical condition, expressed wishes, and degree of suffering; the law permits withholding/non-initiation per those wishes and status, mandates proportional symptom relief, and prohibits withdrawal of continuous life-sustaining treatment. |
| 27. Requests for assisted death | Not addressed in the law text. | No content identified | Explicitly prohibited. Euthanasia and assisted suicide are not permitted. Requests to hasten death cannot be granted and do not diminish the duty to provide proportional symptom relief and comfort care. The Law allows withholding/non-initiation per patient wishes but forbids withdrawal of continuous life-sustaining treatment; disputes may be referred to institutional/national committees. |
| 28. Standards | The law establishes minimum standards for the operation of palliative care structures, including the composition and qualifications of interdisciplinary teams, infrastructure requirements, and quality criteria. It provides for the issuance of regulatory acts to define technical specifications, licensing procedures, and evaluation mechanisms. The aim is to ensure consistent, safe, and high-quality service delivery across all settings. | The regulation sets binding national standards for palliative care provision, covering service levels, staffing ratios, facility and equipment requirements, hygiene and safety conditions, clinical documentation, and the use of national protocols, including paediatric guidelines. It also mandates annual activity reporting and compliance monitoring by the Ministry of Health to ensure consistency and quality nationwide. | Procedural and ethical standards include: Art. 2’s exclusive consideration of medical condition, will, and suffering; precise eligibility definitions (“terminally ill,” “end-stage”); mandatory documentation; regulated advance directives/proxies; oversight by institutional and national committees; and authority for the Minister of Health to issue implementing regulations (Art. 60). |
| 29. Financing | The law does not establish specific funding mechanisms or budgetary allocations for the implementation of palliative care. However, Article 10 provides that services delivered by certified Palliative Care Structures may be reimbursed by public or private insurance bodies, according to criteria defined in future regulatory acts. Additionally, Article 12 allows for monetary donations from individuals or legal entities to support the objectives of palliative care structures, which can be accepted under the applicable provisions of public financial management. | The regulation does not establish a dedicated funding mechanism. It states that palliative care services are integrated into the national health system and financed through existing health insurance and public health resources, with costs covered according to the applicable reimbursement rules for authorised providers. | No specific provisions on funding mechanisms or financial coverage for implementing the law are identified. |
| 30. Additional aspects | The law establishes: A National Palliative Care Patient Registry (Article 7, Part B).  A National Committee for the Development of Palliative Care (Articles 8–9, Part B).  A National Action Plan for Palliative Care (Article 5, Part B) | The regulation includes detailed procedural templates for the annual activity reports of both specialised and basic palliative care providers, a mandatory self-declaration form for home care authorisation, and standardised clinical record formats for adults and children. | Conscientious objection with mandatory transfer of care; exemption from liability when acting under the Law; confidentiality rules (with limited disclosures) plus a duty to check/notify via the national advance-directives database; “status quo” obligation while a committee review is pending and the Attorney General may appear; a patient’s right to arrange additional actions at own expense; and special regimes for minors and protected persons. |
| 31. Subsequent regulations | Future regulation is explicitly foreseen through ministerial decisions and presidential decrees (Article 13, Part B) | ORDIN nr. 253/2018 was updated by ministerial orders in 2022, 2023, and 2024, introducing new home care equipment standards, paediatric provisions, clinical documentation templates, revised reporting procedures, and enhanced Ministry of Health oversight, aiming to expand, standardise, and strengthen palliative care service delivery and monitoring nationwide | Director-General Circular No. 30/09 (July 12, 2009) mandated system-wide availability of palliative services in hospitals, community and long-term care, defined minimum multidisciplinary team standards, credentialing and hours, and set a four-year implementation timeline with one-year progress reporting. Guidance, official forms/annexes and clinical resources are disseminated locally, while the National Center for Palliative Care Research and Education supports implementation and training infrastructure. |

# **Supplementary Table S6** Content Analyses of the National Palliative Care Legal Frameworks of Slovakia and Poland.

| Indicators | Slovakia | Poland |
| --- | --- | --- |
| 1. WHO Region | EURO | EURO |
| 2. Income classification | HIC | HIC |
| 3. Level of palliative care development | Established | Established |
| 4. Year of enactment | 2022 | 2013 |
| 5. Law title along with the reference | Zákon č. 267/2022 Z. z. z 29. júna 2022, ktorým sa mení a dopĺňa zákon č. 576/2004 Z. z. o zdravotnej starostlivosti, službách súvisiacich s poskytovaním zdravotnej starostlivosti a o zmene a doplnení niektorých zákonov v znení neskorších predpisov a ktorým sa menia a dopĺňajú niektoré zákony.  <https://static.slov-lex.sk/pdf/SK/ZZ/2022/267/ZZ_2022_267_20220801.pdf> | Rozporządzenie Ministra Zdrowia z dnia 29 października 2013 r. w sprawie świadczeń gwarantowanych z zakresu opieki paliatywnej i hospicyjnej.  <https://dziennikustaw.gov.pl/DU/2022/262> |
| 6. Law title in English | Act No. 267/2022 Coll. of 29 June 2022 amending and supplementing Act No. 576/2004 Coll. on Health Care, Services Related to the Provision of Health Care, and on Amendments to Certain Acts, as amended, and amending and supplementing certain other acts. | “Regulation of the Minister of Health of 29 October 2013 on Guaranteed Services in the Field of Palliative and Hospice Care.” |
| 7. Word count | 6201 | 5440 |
| 8. Structure | The law is structured into 6 articles, covering amendments to healthcare legislation including definition of palliative care; establishment of support teams; introduction of levels of healthcare need; provisions for healthcare delivery and patient rights; obligations of healthcare and insurance providers; data reporting requirements; and transitional and effective date provisions. | The regulation consists of 12 sections (§1–§12) that define the scope, definitions, organisation and conditions for providing palliative and hospice care services, including a specific provision on perinatal palliative care (§7a), and two annexes: Annex No. 1, listing diseases qualifying for palliative care, and Annex No. 2, specifying detailed personnel, equipment, and organisational requirements for service provision. |
| 9. Contents | The Act establishes a framework for long-term, follow-up, and palliative care, introduces support teams, defines levels of healthcare need, and clarify provider roles, and insurance duties, with implementation from August 2022. | The regulation defines the organisation and conditions for providing guaranteed palliative and hospice care services, including eligibility criteria (§9), settings of care such as inpatient, home, outpatient and perinatal palliative care (§4), the scope of services provided (§5–§7a), financing aspects (§8), and the list of diseases qualifying patients for palliative care (Annex No. 1). |
| 10. Purpose | Integrate long-term and palliative care into Slovak healthcare law; clarify roles and responsibilities. | The regulation aims to define the list and conditions for providing guaranteed palliative and hospice care services financed from public funds, and to determine the level of public financing for medical transport in cases not covered by Article 41(1) and (2) of the Act of 27 August 2004 on health care services financed from public funds(§1) |
| 11. Objectives | The document does not explicitly define “objectives” as a separate section but it clearly establish definitions; set healthcare duration; introduce levels of care needs; and update insurer duties; generally strengthening PC integration | The regulation aims to define key elements for the provision of palliative and hospice care, including the scope of services provided (§5–§7a), the settings in which care is delivered (§4), the eligibility criteria and referral requirements for patients (§9), and the organisational, personnel and equipment requirements for service providers (Annex No. 2). |
| 12. Principles | Multidisciplinary approach; patient rights; timely, quality, and accessible care; social and spiritual support. | Palliative and hospice care is defined as comprehensive and holistic care and symptomatic treatment for patients with incurable, progressive, life-limiting diseases, aimed at improving quality of life, preventing and relieving pain and other somatic symptoms, and alleviating psychological, spiritual, and social suffering, and provided in accordance with current medical knowledge (§3). |
| 13. Palliative care right | Patients with incurable progressive illness are entitled to palliative care as a legal right. | The regulation establishes that patients suffering from incurable, progressive, life-limiting oncological and non-oncological diseases are entitled to receive guaranteed palliative and hospice care services, provided that they meet the medical indications and referral requirements specified in the regulation (§3(2); §9). |
| 14. Palliative care definition | Healthcare provided to a person with an incurable and progressive disease that typically leads to death, aimed at alleviating suffering and maintaining the person's quality of life | Palliative and hospice care is defined as comprehensive, holistic care and symptomatic treatment for patients with incurable, progressive, life-limiting diseases not amenable to causal treatment, aimed at improving quality of life and preventing and relieving pain and other somatic symptoms, as well as alleviating psychological, spiritual and social suffering (§3(1)). |
| 15. Society | The document introduces support teams in inpatient facilities to provide social assistance and spiritual services. While families are not granted formal decision-making rights, physicians must inform patients about palliative care options, indirectly supporting patient and family involvement in care decisions | The regulation does not address society in a explicit way. Community involvement, volunteers, public awareness, societal responsibility, or civic participation) are not mentioned in the law text. |
| 16. Governance | The Ministry of Health oversees provider lists, defines data reporting requirements, and receives anonymized data from insurers. Health insurance companies must monitor availability, quality, and efficiency of long-term nursing care and decide on extensions of care. | Governance is addressed through the regulation issued by the Minister of Health, which establishes the legal framework for guaranteed palliative and hospice care services under the Act of 27 August 2004 on health care services financed from public funds and defines organisational conditions and requirements for service provision (§1; §4–§10; Annex No. 2). Operational governance responsibilities are not explicitly described in the regulation. |
| 17. Research | The document does not establish obligations or support for research. It only mandates data collection and reporting by insurers and providers for monitoring availability, quality, and efficiency of care. | Research is not addressed in the regulation. |
| 18. Medicines | There are no explicit provisions on medicines. Access is implied within palliative care delivery but without specific regulations on essential medicines or opioids. | The regulation addresses medicines in the context of care provision, stating that pharmacological treatment and pain management using medicinal products available in the territory of the Republic of Poland must be provided as part of palliative and hospice care services, and that medicinal products are provided free of charge in inpatient settings (§5(3)–(4); §8(1)). Opioids are not explicitly mentioned in the regulation. In Poland, access to medicines, including opioids, is regulated through general legal acts governing the healthcare system, including provisions concerning access to medicinal products. |
| 19. Undergraduate educatiion | No provisions on undergraduate education | Undergraduate education in palliative care is not addressed in the regulation. |
| 20. Continuing education | Not addressed. The Act does not regulate continuing education or professional development in palliative care. | Continuing education is not addressed in the regulation. The text specifies qualification and training requirements for health professionals providing palliative and hospice care services but does not include provisions on continuing education. |
| 21. Specialization | The Act recognizes palliative medicine as a medical specialization and requires it for certain services (e.g., hospices, mobile hospices, palliative departments). Basic palliative healthcare may be provided by a physician specialized in another medical field. | The regulation requires that palliative and hospice care services be provided by physicians specialized in palliative medicine or physicians undergoing specialization in this field, and by nurses specialized in palliative care nursing or undergoing such specialization, as part of the personnel requirements for service providers (Annex No. 2). |
| 22. Integration of services | The Act integrates follow-up, long-term, and palliative care into the healthcare system. It requires a multidisciplinary approach and patient instruction, linking medical, social, and spiritual dimensions of care. | The regulation addresses integration through the organisation of palliative and hospice care across multiple care settings, including inpatient hospices or palliative medicine wards, home hospices, outpatient palliative medicine clinics, and perinatal palliative care services, allowing care to be provided in different settings according to patient needs (§4). |
| 23. Specialized services | Specialized palliative care includes outpatient (clinics, mobile hospices) and inpatient services (palliative departments, hospices). Duration is limited (1–6 months) but may be extended with insurer approval. | The regulation establishes the provision of specialized palliative and hospice care services delivered in inpatient hospices or palliative medicine wards, home hospices for adults and children, outpatient palliative medicine clinics, and perinatal palliative care settings (§4), and requires that service providers meet specified personnel qualifications and training requirements (Annex No. 2). |
| 24. Palliative sedation | Not mentioned in the law text. | Palliative sedation is not addressed in the regulation. |
| 25. Advance care planning | Advance care planning is not explicitly regulated. Physicians must instruct patients on palliative options when indicated, indirectly supporting informed decisions but without a formal ACP framework. | Advance care planning is not addressed in the regulation. The text does not include provisions regarding advance directives, advance care planning, or future treatment preferences of patients. |
| 26. Adaptation of therapeutic effort | Implicitly recognized: when curative options are exhausted, care shifts to palliative focus. No explicit terminology or clinical guidance is provided. | Adaptation of therapeutic effort is explicitly mentioned in the context of perinatal palliative care, which includes ensuring comfort and protection against persistent therapy for newborns with severe and irreversible impairment or incurable life-threatening disease (§2(3)(b)). The regulation does not contain broader provisions on limitation or withdrawal of treatment. |
| 27. Requests for assisted suicide | Not mentioned in the law text. | Not mentioned in the law text. Requests for assisted death are not regulated in Poland, as the Penal Code prohibits assisted dying and classifies it as a criminal offence. |
| 28. Standards | Standards are defined through levels of healthcare need in Annex No. 2 (basic, moderate, high), which determine eligibility and scope of long-term and follow-up care. It requires a multidisciplinary approach and patient instruction in palliative care situations (6ba). No explicit quality indicators, outcome measures, or minimum staffing levels are specified | The regulation establishes comprehensive standards for the provision of palliative and hospice care services, including the settings in which care must be delivered (§4), the scope of services to be provided (§5–§7a), eligibility and referral requirements for patients (§9), and detailed organisational, personnel, equipment, and operational requirements for service providers set out in Annex No. 2. |
| 29. Financing | Long-term and palliative care are covered under public health insurance. Reimbursement follows Ministry pricing regulations, with transitional rules for 2022–2024 to ensure payment while new systems are implemented. | The regulation establishes that palliative and hospice care services are provided as guaranteed health care services financed from public funds, and specifies that diagnostic tests, medicinal products, and medical devices necessary for care are provided free of charge to patients, while medical transport in certain cases is financed at 40% from public funds (§1; §8). |
| 30. Additional aspects | It introduces support teams (social/spiritual), crisis intervention for patients and families, home mechanical ventilation, and harmonization with related health laws. | The regulation includes specific provisions for perinatal palliative care and bereavement support for parents, and provides detailed eligibility criteria for palliative care based on ICD-10 disease lists (§5(11); §7a; Annex No. 1). |
| 31. Subsequent regulations | The Act establishes transitional reimbursement rules (2022–2024) and mandates Ministry guidelines for data reporting by insurers and providers. No binding secondary regulations specific to palliative care were identified. Related frameworks (e.g. Patient’s Rights Charter, reports on hospice development and funding) provide context but do not constitute subsequent regulations to the Act. | The Regulation of the Minister of Science and Higher Education of 26 July 2019 establishes mandatory undergraduate curricula for medical professions, including palliative care education. |

# **Supplementary Table S7** Summary of Interviews with Key Informants of France and Belgium.

| France | | Belgium |
| --- | --- | --- |
| Time | *June 13, 2025* | *July 23, 2025* |
| Interviewer | *TC* | *LM* |
| Interviewee | *GM, JC, TG* | *JM* |
| Executive Summary | | |
| The legal framework for palliative care in France has evolved through a series of important laws beginning in 1995, culminating in the 2016 Clayes-Leonetti law. The first law enacted in 1995 focused on pain management whereas the main objective of the law enacted in 1999 was to establish a framework for the right and universal access to palliative care regardless of the living or care setting. The law aimed to establish palliative care as a fundamental right for all patients, particularly emphasizing comprehensive, multidisciplinary care and the importance of dignified end-of-life care. Although the 1999 law set important legal precedents and laid the groundwork for later developments, its implementation faced significant challenges. While it guaranteed the right to palliative care, particularly for patients in terminal stages of illness, the application of this right has been uneven across regions. The main implementation challenges of the law were that the implementation across regions was uneven and there is a huge lack of trained professionals and difficulty of coordination between home institutions and hospitals. However, although, these challenges are still present, they have improved over time.  Subsequent laws, including the 2002 Kouchner Law, the 2005 Leonetti Law, and the 2016 Clayes-Leonetti Law, built upon the 1999 framework, addressing patient autonomy, advanced directives, and deep continuous sedation. The 2002 Kouchner Law aimed to strengthen patient autonomy and health democracy. The 2005 Leonetti Law was in response to evolving societal needs and introduced significant protections for patients, including the right to reject medical interventions and the promotion of end-of-life decisions through advanced directives. The very last law in France, the 2016 Clayes-Leonetti Law has been proposed to respond to a strong public and political demand for clarification following several public reports and cases and a national debate. The objective was to create a right to deep and continuous sedation until death. In addition, the aim was to clarify advance directives that were enacted in the 2005 Leonetti Law.  Despite these legal advancements, France continues to face several challenges in the practical application of its palliative care laws. Unequal access across regions, insufficient profesional training, and fragmented care delivery remain significant barriers. Additionally, there is a lack of dedicated palliative care facilities such as hospices, and the awareness of palliative care rights among patients and healthcare professionals is still limited. Recent government strategies and proposals, including a national budget of 100 million euros for palliative care development, are aimed at addressing these issues, focusing on improving training, increasing resources, and expanding access to services.  In summary, while the legal framework in France has established palliative care as a right and has made significant progress in protecting patient dignity and autonomy at the end of life, challenges remain in ensuring equitable access to services and full implementation of the law across all regions. | | Belgium’s national palliative care law, enacted in 2002, marked a critical turning point for end-of-life care. Emerging as part of a political compromise alongside the euthanasia law, it recognized palliative care as a fundamental right for every patient at the end of life. The legislation mandated the integration of palliative care across all care settings including hospitals, home care, and nursing homes by requiring the presence of palliative referent nurses in nursing homes, and introduced structural obligations such as palliative support teams in hospitals and a network of multidisciplinary home care teams.  A distinctive feature of the Belgian approach was its integration with two other laws: one on euthanasia and one on patients’ rights to information, which states other patient rights too. This framework not only legitimized open communication about end-of-life options but also aimed to ensure that requests for euthanasia only could be honoured after the obligation to talk to the patient about palliative care options by experienced palliative caregivers.  The law established obligations for structural developments but partial reimbursement: hospitals must have palliative support teams, 360 dedicated palliative care beds were distributed across the country, and country wide home-based palliative care services were installed. Palliative care is for all patients free of charge. However, funding remains a major challenge, as only about 80% of the real costs are covered by both the government and health care services, forcing services to seek additional resources.  Over two decades, the law has progressively normalized palliative care, reduced taboos, facilitated significant progress in symptom control (also social, psychological and spiritual issues), communication, and community collaboration. Yet, critical gaps persist, particularly in education, research, and specialist training alongside resource constraints and inequities between regions. Belgium’s law is rights-based but lacks robust enforcement mechanisms and comprehensive quality assurance.  The law was introduced in 2002 as part of a broader legislative package on euthanasia and patient rights, aiming to ensure balanced access to end-of-life options. It guarantees the right to palliative care for every patient at the end of life and mandates the presence of palliative care structures across all care settings, free of charge for the patients. Hospitals are legally required to have palliative support teams, with minimum staffing ratios in function of bed numbers.  A national cap of 360 palliative care beds, geographically distributed for equity, was established but remains unchanged after 20 years, while the aging population and the burden of chronic comorbidities ask for more. Palliative home care teams are available nationwide and accessible upon request by patients, general practitioners or nurses.  Funding remains partial (around 80%), leaving services reliant on hospital contributions and fundraising.  Implementation challenges include workforce shortages, lack of palliative care in medical curricula (now partly addressed), and slow progress in integrating palliative care into specialist education.  The law includes audit requirements to ensure ongoing education and quality but lacks specificity on educational content and research promotion.  Progress has been significant in awareness, collaboration, and care quality, but major gaps remain in education, funding, and integration into specialist training. |
| 1. What was the initial motivation for the law, who promoted it and what were its main objectives and implementation challenges? | | |
| The first law was enacted in 1995 with a motivation to recognize pain as a public health issue. The objective was to integrate pain management into caregiver training and to care itself. The first challenge was that the resources were very limited and there was a very weak implementation of palliative care at that time. The main objective of the law enacted in 1999 was to establish a framework for the right and universal access to palliative care regardless of the living or care setting. The law aimed to establish palliative care as a fundamental right for all patients, particularly emphasizing comprehensive, multidisciplinary care and the importance of dignified end-of-life care. The law was promoted in response to a growing societal awareness of the unhealthy nature of dying, and the importance of offering compassionate, comprehensive care at the end of life. Subsequent laws, including the 2002 Kouchner Law, the 2005 Leonetti Law, and the 2016 Clayes-Leonetti Law, built upon the 1999 framework, addressing patient autonomy, advanced directives, and deep continuous sedation.  Main Objectives of the Law, enacted in 1999:   - Universal Right to Access Palliative Care:   The law affirmed that every patient, regardless of their illness, has the right to access palliative care if their condition warrants it.   - Provision of multidisciplinary care:   The law aimed to ensure that multidisciplinary care including pain management was integrated into medical practice, caregiver training, and healthcare evaluation.   - End-of-Life Care Recognition:   The law intended to address the needs of patients at the end of life, emphasizing human dignity and the importance of alleviating suffering, particularly in terminal illnesses.  Implementation Challenges of the law enacted in 1999:   - Regional Disparities:   Despite the law’s universal intention, access to palliative care services is not uniform across France, with significant disparities between urban and rural areas. However, these challenges have improved over time, though there are still major gaps in the access and delivery of palliative care across different regions in France.   - Insufficient Professional Training   A major challenge in the 1999 law's implementation was the lack of properly trained healthcare professionals, particularly in palliative care, leading to inconsistent care provision.   - Coordination Issues:   There were difficulties in coordinating palliative care services between hospitals, home care, and other healthcare institutions. This led to fragmented care for patients, especially those in rural areas or less well-resourced regions.   - Public Awareness:   There was insufficient public awareness regarding the existence of the law and patients' rights to palliative care, which prevented many people from accessing the services they were entitled to.   - Resource Constraints:   The implementation of the law was hampered by limited resources, both in terms of trained personnel and dedicated palliative care facilities, such as hospices. | | The Belgian palliative care law arose at a critical time in the country’s healthcare debate. In the late 1990s and early 2000s, multiple voluntary initiatives in hospitals and communities provided fragmented palliative care, but access and quality were uneven. Simultaneously, a strong political and philosophical debate pushed for euthanasia legislation, creating the window to establish also a legal framework for palliative care. The 2002 compromise combined three laws on euthanasia, patient rights, and palliative care, marking a cultural and political shift toward transparency and patient autonomy. By anchoring the right to palliative care in law, Belgium sought to improve quality of life for patients facing terminal illnesses and facilitate honest communication about end-of-life decisions.  The law established clear structural obligations to operationalize this right. Every hospital must maintain a palliative support team comprising a physician, nurse, and psychologist, with staffing ratios adjusted to bed capacity. Additionally, the law mandated 360 residential palliative care beds distributed geographically to ensure equitable access and created a framework for multidisciplinary home care teams to deliver free services. To maintain quality, the law introduced audits every two to three years to verify staff expertise, training and compliance. Despite these achievements, implementation faced significant hurdles, such as those previously mentioned in nursing homes. Funding covers about 80% of the costs, forcing hospitals, care homes and home care teams to seek donations or absorb costs. Integration with home care was a slow growing trajectory of collaboration between the involved stakeholders and functions now very well, while education gaps persists as palliative care was nearly absent from basic medical curricula for two decades, and Belgium still lacks official professorships in the field. Furthermore, resource limitations and competing political priorities continue to challenge progress.  In terms of objectives, the law intended to:  Universal access to palliative care as a legal right.  Mandate the presence of palliative support teams in all hospitals, in care homes of more than 60 beds.  Ensure national coverage of residential palliative care through a geographically spread of a fixed number of palliative care beds.  Ensure a country wide coverage by palliative home care services  Facilitate open communication about end-of-life care by combining laws on euthanasia and patient rights.  Implement quality monitoring through regular audits.  Provide financial support via partial reimbursement (80%) for services.  In summary, the implementation challenges were:  1. Partial funding leading to financial instability for teams.  2. Home care integration: development of confident collaboration between the regular home care teams with the palliative home care services.  3. National bed cap (360) unchanged for over 20 years.  4. Educational gaps: Insufficient formal inclusion of palliative care in basic curricula still existing in 2025.  5. Lack of professorships and limited academic investment in palliative care.  6. Regional disparities between Flanders and Wallonia.  7. Persistent competition for resources in a healthcare system dominated by costly interventions. |
| 2. How does the law promote palliative care in your country? | | |
| The 1999 law promotes palliative care by establishing the right for every patient to access multidisciplinary palliative care, regardless of their condition. It set out a legal framework for integrating palliative care into the healthcare system, which was previously underdeveloped. The law also played a key role in raising awareness of the importance of palliative care, ensuring that patients’ dignity was respected at the end of life. However, the law’s impact has been hindered by challenges in practical application, especially regarding the training of healthcare professionals and regional disparities in care access.  To address existing gaps, the French government has introduced a comprehensive strategy to strengthen palliative care. With a dedicated budget of 100 million euros per year, this strategy focuses on improving information, training, research, and the organization of palliative care services. A key part of this strategy is the introduction of new structures for palliative care, as  France currently lacks hospices and relies on hospitals, home care services, and services for the aging population. This proposal is aimed at expanding dedicated palliative care facilities to ensure comprehensive, specialized care. In terms of awareness of palliative care, COVID infection resulting in countless (and isolated) deaths was a major driving factor in France. However, a recent national survey conducted in France showed that there is still a substantial lack of objective knowledge and awareness on end-of-life care:  <https://www.sciencedirect.com/science/article/abs/pii/S1636652223000211>).  1. Empowering Societies to Care:   - The law empowers both healthcare professionals and the public by recognizing palliative care as a fundamental right. However, a recent national survey conducted in France showed that there is still a substantial lack of objective knowledge and awareness on end-of-life care. (<https://www.sciencedirect.com/science/article/abs/pii/S1636652223000211>). - The role of volunteers is formalized in the law, allowing them to act as part of the support system. This point reinforces the community and compassionate dimension of palliative care, recognizing the valuable contribution of volunteers in providing care and support to patients and their families in situations of suffering at the end of life.   2. Supportive Health Policies:   - The 1999 law laid the foundation for subsequent policies promoting palliative care, including the establishment of guidelines for universal access to services. - However, implementation across regions remains uneven, and there are still significant challenges in aligning national policies with local practices, particularly in underserved areas.   3. Research:   - The law promotes the integration of palliative care into the healthcare system and it is addressed in the law that university hospitals should support the research in palliative care. - More recent efforts, like surveys and research on the public’s awareness of palliative care, are beginning to contribute to improving care.   4. Education and Training:   - The 1999 law addresses training for healthcare professionals, but this mandate has faced challenges in terms of actual implementation.   5. Access to Essential Medicines:   - The 1999 law does not mention or promote medications in palliative care.   6. Integrated Services:   - The law aims to integrate palliative care into healthcare settings, however, the lack of dedicated palliative care facilities, such as hospices, has made it difficult to offer comprehensive care to patients who need specialized end-of-life care. | | The law promotes palliative care primarily through institutional mandates and financial support, though only partial. By requiring hospitals to have palliative support teams and ensuring home care availability nationwide, it significantly improved structural access. Patients benefit from free home-based services once labelled as palliative, and additional benefits such as lower costs for medical visits and therapies. The law also facilitated conversations about end-of-life care, reducing previous taboos and fostering a cultural shift toward shared decision-making. Multidisciplinary teams have played a vital role in disseminating best practices within hospitals and the community, indirectly optimizing symptom control standards across specialties.  However, gaps remain in key systemic areas. While the law obligates continuing education and periodic audits, it does not define educational content in basic curricula or enforce integration into specialist training, leaving large variations in competency. It lacks explicit provisions for research and symptom control practices. Nursing homes remain underfunded and struggle to meet requirements, such as employing a palliative care-trained nurse. Over two decades, progress in integration has been driven more by professional commitment and informal collaboration than by comprehensive legislative mechanisms.  1. Empowering Societies to Care:  The law does not include explicit provisions to empower families or communities in caregiving roles. However, it indirectly strengthens empowerment by guaranteeing universal access to palliative care and establishing the patient’s right to clear and complete information about their illness and treatment options. This legal recognition of the right to information, adopted alongside the palliative care and euthanasia laws, enabled open communication about end-of-life decisions. In addition, the law made palliative home care free of charge and created multidisciplinary teams available nationwide, which patients, families, and healthcare providers can activate at any point. These elements collectively facilitated greater patient and family involvement in decision-making and improved the ability to plan care according to individual needs and preferences.  2. Supportive Health Policies:  This is the law’s strongest dimension. It establishes palliative care as a legal right and mandates structural provisions across the health system, including palliative support teams in every hospital, a fixed number of palliative care beds distributed nationwide, and universal home care services. Regular audits ensure compliance with staffing and training requirements, creating accountability within the system.  3. Education and Training  Education is only partially addressed. While audits monitor whether team members complete continuing education, the law does not define the content or scope of such training, and it does not mandate integration of palliative care into basic and specialist medical curricula. Progress in this area has largely depended on voluntary efforts by professionals and advocacy groups, with formal academic leadership still lacking.  4. Research in Palliative Care  The law does not address research or allocate resources for it. Advancements in knowledge and practice have come mainly through professional initiatives and collaboration among universities, rather than through legislative support.  5. Integration of professional expert palliative care as partner in the multiprofessional health care system  The law made a significant contribution by mandating integration of palliative care into hospitals, home care, and hospice settings. Hospital-based palliative teams and home care services ensure structural integration across levels of care, while hospice beds offer additional support. However, nursing homes remain underfunded and poorly supported, with only minimal obligations and no dedicated reimbursement for palliative care, limiting full system-wide integration. |
| 3. Does the law—as it stands—secure the progress made toward ensuring patients’ right to palliative care, and if it does, could you explain how? | | |
| The law, enacted in 1999 was instrumental in establishing palliative care as a legal right. It has secured progress by creating a legal framework that obligates healthcare providers to offer palliative care, thus recognizing it as an essential part of medical practice. However, while the law has made strides in formalizing palliative care rights, its practical application remains challenged in France by regional disparities, professional training gaps, and limited resources.  Limitations of the Law enacted in 1999:  • Limited Professional Training: While the law promotes training, it does not emphasize undergraduate training or specialization in palliative care.  • Coordination Problems: The law's goal of integrating palliative care across various healthcare settings has not been fully realized due to difficulties in coordination between hospitals, home care, and other healthcare services.  • Medications, sedation and advances directives: The law enacted in 1999 does not mention medications or access to medications in palliative care, no sedation either. However, Subsequent laws, including the 2002 Kouchner Law, the 2005 Leonetti Law, and the 2016 Clayes-Leonetti Law, built upon the 1999 framework, addressing patient autonomy, advanced directives, and deep continuous sedation. | | The law formally guarantees the right to palliative care and has significantly improved access over the last two decades. Patients now benefit from institutionalized services across hospitals and communities, and the requirement for audits promotes a baseline of quality assurance. These provisions have fostered a cultural transformation, making end-of-life care discussions more common and care more patient-centred. However, the right established by the law is not fully enforceable in practice due to structural and resource gaps.  Persistent limitations hinder comprehensive implementation. Financial support remains partial, leading to dependence on fundraising and institutional budgets. The static cap of 360 beds does not reflect growing demand, and while home care coverage is universal, nursing homes operate underfunded mandates. Education has seen incremental progress but lacks depth, with minimal integration into specialist curricula and no formal academic positions. Furthermore, the law does not address essential components such as research promotion or medication access. Consequently, while the law provides a strong foundation, achieving equitable and high-quality palliative care nationwide requires continued advocacy, policy revisions, and strategic investment.  Major limitations of the law include:  1. Implementation of the law is merely limited to ‘terminal’ care with expected survival of less than 3 months.  2. 80% reimbursement forces services to seek supplementary funding.  3. Static national capacity limits hinder scalability.  4. Lack of comprehensive educational mandates and professorships.  5. Regional inequities in implementation.  6. Absence of legal provisions for research and essential medicines.  7. Underfunded obligations for nursing homes. |
| Additional aspects and remarks | | |
| • Proposals for New Structures:  The government is working on creating new structures for palliative care, since France lacks dedicated hospices for end-of-life care. This could involve setting up more specialized facilities to provide comprehensive end-of-life care.  • Increased Funding and Strategy:  With a 100 million euro per year strategy, the French government is focusing on improving training, resources, research, and public information to strengthen the palliative care system.  • Surveys and Research:  Ongoing surveys and research are essential to tracking public awareness and the evolution of the laws, particularly regarding advance directives and trust persons, helping to guide future policy and improve the implementation of palliative care.  • Challenges Post-COVID:  The COVID-19 pandemic has brought more attention to end-of-life issues, increasing public and professional interest in anticipating end-of-life wishes and ensuring that patients and caregivers receive high-quality and proper care at the end of life.  • Legal Proposals:  Two new law proposals, one relating to the development of palliative care, the other relating to the right to assisted dying recently underwent voting at the National Assembly and will be further evaluated and discussed in the Senate in September and October 2025. | | Strategic Linkage with Euthanasia Legislation:  The law’s simultaneous adoption with the euthanasia law was a deliberate strategy to ensure that palliative care became an integral component of end-of-life decision-making. This alignment guaranteed that euthanasia would only be considered after the obligation to talk to the patient about palliative care options by experienced palliative caregivers  Financial Incentives for Patients:  Once a patient is formally labelled as “palliative,” home care becomes free of charge, and medical consultations, nursing visits, and specific therapies are significantly reduced in cost. This provision helps remove financial barriers, ensuring patients and families have better access to coordinated care during the final stages of life  Influence of Political and Economic Lobbying:  Healthcare priorities in Belgium are heavily influenced by political and economic interests, with pharmaceutical industries wielding strong lobbying power due to their contribution to employment and the economy. This often results in continued funding for expensive treatments at the end of life, even when their benefit is limited, while palliative care struggles to secure resources. Johan Menten noted that reallocating even a fraction of the money spent on aggressive therapies could greatly improve palliative care access and quality. However, reducing drug use threatens pharmaceutical revenues and employment, making it politically sensitive and leaving palliative care at a disadvantage in budget negotiations.  Cultural Transformation on End-of-Life Issues:  Over the past two decades, Belgium has experienced a profound cultural shift. Palliative care, once associated with hopelessness and loss, is now widely recognized as an essential element of patient care. This normalization has fostered open communication among patients, families, and healthcare providers, paving the way for shared decision-making.  Limited Scope on Advanced Care Planning and Community Sensitization:  While the law mandates structural access to palliative care, it does not include advanced care planning or large-scale community sensitization initiatives. These remain critical to aligning care with patient preferences and enabling timely, informed choices, yet they rely mainly on voluntary initiatives.  Challenges in Specialist Education Integration:  Despite progress in basic medical education, integrating palliative care into specialist training remains a persistent challenge. Curricular constraints and competing priorities hinder the inclusion of palliative principles in disciplines such as cardiology, nephrology, and oncology, where patients often require this type of care.  Optimism for Continued Progress  Despite these limitations, Johan Menten expressed optimism about the future trajectory of palliative care in Belgium. Compared to three decades ago, significant advancements have been achieved in symptom control, communication, and structural integration. Continued advocacy and targeted reforms are seen as crucial to consolidating and expanding these gains. |

# **Supplementary Table S8** Summary of Interviews with Key Informants of Luxembourg and Italy.

| Luxembourg | | Italy |
| --- | --- | --- |
| Time | *July 30, 2025* | *August 25, 2025* |
| Interviewer | *LM* | *CC* |
| Interviewee | *HS* | *CP* |
| Executive Summary | | |
| The detailed one-by-one responds to the questions are provided below. | | The detailed one-by-one responds to the questions are provided below. |
| 1. What was the initial motivation for the law, who promoted it and what were its main objectives and implementation challenges? | | |
| The main motivations were as follows:  - To organize the right to palliative care for anyone with an incurable illness in the final stage of life, whether in a hospital or non-hospital setting.  - To provide for the coverage of expenses related to palliative care by health insurance and long-term care insurance.  - To avoid therapeutic obstinacy and prevent legal action against doctors if they consider certain examinations inappropriate in relation to the patient's condition. Also, to allow patients to express their wishes in advance via a written document called an “advance directive.” The advance directive was integrated into the legal text to avoid multiple conflicting documents. The Ministry of Health and the Ministry of Family Affairs published a unified document presenting the advance directive and end-of-life provisions (including a declaration of euthanasia):  <https://santesecu.public.lu/fr/publications/m/ma-volonte-en-fin-de-vie-2019-fr-de-en-pt.html>  References: <https://www.cc.lu/avis-legislation/detail/projet-de-loi-relatif-aux-soins-palliatifs-et-a-laccompagnement-en-fin-de-vie-2817bje#_ftn2>  The palliative care law in Luxembourg was promoted by the Luxembourg government, particularly the Ministries of Family and Integration, and of Health. The bill, which also included the advance directive and end-of-life accompaniment, was passed in a first reading by the Chamber of Deputies.  Ministry responsibilities:  - Ministry of Health: implements public health and social security policy ensuring quality, accessibility, equity, and financial sustainability of healthcare.  <https://m3s.gouvernement.lu/fr.html>  - Ministry of Family, Integration and the Greater Region (now Ministry of Family, Solidarity, Living Together, and Reception): responsible for family policy, elderly and disability support, intercultural integration, anti-poverty measures, and reception services.  <https://mfsva.gouvernement.lu/fr/le-ministere/attributions.html>  Additional References: <https://gouvernement.lu/fr/actualites/toutes_actualites/articles/2006/07/20bartolomeo_jacobs.html> <https://gouvernement.lu/fr/actualites/toutes_actualites/articles/2008/12-decembre/18-chd.html> | | According to the key informant, the motivation for Law 38/2010 came from a broad consensus among different actors: professionals in palliative care, NGOs (such as Fondazione Floriani and many others), patients’ and families’ associations, and the Catholic Church, which strongly advocated for attention to the needs of people at the end of life. Their joint effort, together with political actors, made it possible to draft and approve a very comprehensive law with good definitions and clear objectives.  The main objectives were to recognize the right of all citizens to access palliative care and pain therapy, to create three distinct national networks (for pain therapy, adult palliative care, and pediatric palliative care), and to guarantee quality, dignity, and support for both patients and families.  However, the major implementation challenge has been Italy’s decentralized health system. With 20 different regional health authorities, the application of the law has varied greatly, leading to significant inequalities. While some regions developed strong services, others lagged behind. This regional diversity remains the principal difficulty in translating the law’s principles into uniform practice across the country. |
| 2. How does the law promote palliative care in your country? | | |
| <https://legilux.public.lu/eli/etat/leg/loi/2009/03/16/n1/jo>  The law of March 16, 2009, regulates access to palliative care for any person with a serious and incurable illness in its advanced or terminal stage. It also governs advance directives and introduced accompaniment leave.  How the law promotes palliative care:   - Right to palliative care: Establishes the right to access palliative care for all who need it, regardless of the illness’s cause. - Financial coverage: The National Health Fund (CNS) covers palliative care costs upon recommendation by the Medical Control of Social Security. - Duration and coordination of care: Initially granted for 35 days, renewable in 35-day periods. A centralized care booklet tracks the patient through all care settings, promoting smooth coordination among providers. - Advance directive and trusted person: Patients can express their end-of-life treatment wishes and designate a trusted person with access to their medical file to represent them if they become incapacitated. - Support and accompaniment: Emphasis on supporting patients and their loved ones, including psychological and spiritual care, and bereavement support. There is also a 5-day accompaniment leave for family members. - Avoiding therapeutic obstinacy: Doctors may abstain from procedures deemed inappropriate, and patients may refuse aggressive treatments. - National Plan “End of Life and Palliative Care 2023–2026”: A national plan was created to inform citizens about their rights and tools such as advance directives and end-of-life provisions. - Training: A Grand-Ducal regulation defines training levels for palliative care professionals. 40% of nursing home and home-care network staff must complete a 40-hour awareness course. <https://legilux.public.lu/eli/etat/leg/rgd/2019/02/08/a109/jo> | | Law 38/2010 promotes palliative care by explicitly recognizing it, together with pain therapy, as part of the essential levels of care within the National Health Service. It created three national networks: the pain therapy network, the adult palliative care network, and the pediatric palliative care network, which were designed to ensure continuity of care across settings (hospital, home, and hospice) and to guarantee access throughout the country.  The law also introduced financial provisions, allocating government funds for the development of hospices and services. However, as the key informant underlined, these resources have often been difficult to access and use effectively at the regional and local level, limiting their real impact. The distribution and utilization of funds has been inconsistent and has not always translated into service expansion.  Another weakness was education: the law said very little about undergraduate training, which was a pity, as this would have been a powerful driver for long-term change. Only later, through separate regulations, was palliative medicine recognized as a medical specialty and undergraduate training slowly introduced, but unevenly across universities.  In addition, the law mandated institutional campaigns to inform citizens and reduce stigma around opioid use, required systematic documentation of pain in medical records, and supported the training of professionals and volunteers.  Overall, the law provides a strong framework to promote palliative care, but uneven regional implementation, difficulties in the effective use of financial resources, and the initial lack of commitment to undergraduate education have limited its full potential. |
| 3. Does the law—as it stands—secure the progress made toward ensuring patients’ right to palliative care, and if it does, could you explain how? | | |
| Yes, the law ensures progress by:  1. Recognizing the right to palliative care: available to all regardless of age or care setting, based on a declaration by a physician to the CMSS.  2. Full financial coverage by CNS.  3. Centralized care booklet for seamless care across settings.  4. Legal framework for refusing futile treatments.  5. Advance directive and trusted person provisions.  6. 5-day leave for family caregivers; training for healthcare professionals.  7. National Plan 2023–2026.  Challenges remain:   - Despite progress, care provision remains uneven across regions, even in a small country like Luxembourg. - Omega 90 has organized 40-hour awareness courses for general practitioners over the past 3 years, but hospital physicians show little interest in such training. It is difficult to assess specialists’ training levels. - Communication between actors is diffuclt or nonexistent, especially between hospitals and home-care settings. - There is still major work needed to raise public awareness and inform healthcare personnel about the law and the importance of naming a trusted person and completing an advance directive. <https://www.omega90.lu/p/54> | | According to the key informant, the law has clearly contributed to securing progress, especially by giving visibility to palliative care and placing it firmly in the public arena. Before 2010, many professionals and even local health authorities did not recognize palliative care; today, patients, families, and society at large are aware that palliative care is a right. This cultural shift is one of the most important achievements of the law.  At the political level, another strength is the broad consensus: palliative care is supported by both right- and left-wing parties, creating unity around its importance in Italy. This consensus provides a stable foundation for the future.  However, the law has not fully guaranteed uniform implementation. The decentralized health system has led to significant regional disparities, and governance structures are weak. A commission exists within the Ministry of Health, but more than ten years later, reliable national data are still lacking, and monitoring is poor.  In sum, the law secures patients’ rights at the level of principles and public recognition, but its practical guarantee depends on stronger governance, better data, and more consistent regional implementation. |
| Additional aspects and remarks | | |
| None. | | None. |

# **Supplementary Table S9** Summary of Interviews with Key Informants of Portugal and Germany.

| Portugal | | Germany |
| --- | --- | --- |
| Time | *July 30, 2025* | *September 2, 2025* |
| Interviewer | *CC* | *TC* |
| Interviewee | *MC* | *LR* |
| Executive Summary | | |
| 1. The Portuguese palliative care law was primarily driven by a single professional with experience in the field who held political office. While it was unanimously approved in Parliament, it was never fully embraced as a national cross-party commitment. Its main goal was to give legal recognition to palliative care as a patient right and part of the national health system. However, its implementation faced major challenges: lack of political ownership, absence of enforcement mechanisms, no dedicated funding, and rigid formulations that limit innovation. Although it enabled symbolic progress and some service development, the law has had limited practical impact and has not guaranteed widespread or equitable access to palliative care across the country. 2. The law promotes palliative care mainly by legally recognizing it as a patient right and integrating it into the national health system. It supports health policy development through national commissions and strategic plans, and it encourages the creation of services, especially in hospitals. It also fostered basic education and some postgraduate training, though Portugal still lacks an official medical specialty. However, key areas are not addressed: the law does not empower communities, does not promote or fund research, and makes no provision for access to essential medicines like opioids. Service development remains uneven and poorly integrated. Overall, the law laid a foundation but fails to comprehensively promote palliative care across all WHO framework pillars. 3. The law recognizes the right to palliative care but does not effectively secure it. While it legitimized palliative care within the health system and enabled some progress such as hospital teams and training, its provisions are not mandatory. There are no enforcement mechanisms, dedicated funding, or accountability structures to ensure nationwide access or continuity. Strategic planning is required by law but has not been consistently implemented. As a result, service development depends heavily on local initiative and professional commitment rather than systemic support. In its current form, the law provides a symbolic foundation but lacks the practical tools to guarantee or sustain patients’ rights in practice. 4. The overall assessment made by the key informant, a highly respected palliative care professional in Portugal, about having a national palliative care law is nuanced and realistic. He acknowledges the symbolic and political value of the law: it gave visibility, legitimacy, and institutional presence to palliative care within the health system. For a field that had long been peripheral, this was a critical achievement.   However, his evaluation is not romanticized. He emphasizes that while the law was necessary, it was not sufficient. Its origin meant it lacked broad ownership. Its rigid structure, absence of enforcement, funding, and evaluation mechanisms have limited its practical impact. It enabled some progress, but not in a secure or sustainable way.  Ultimately, the key informant seems to suggest that having a law is better than not having one, but that its true value depends on how it is politically supported, funded, implemented, and adapted over time. For him, the Portuguese law is a starting point, but not yet the tool it could be to truly guarantee equitable, integrated, and quality palliative care for all. | | Germany has a highly developed and integrated palliative care system, but it is primarily governed through broad legislation, negotiated agreements between sickness funds and physician associations, and professional standards**,** rather than detailed statutory law. The Hospice and Palliative Care Law of 2015 was enacted in the context of parliamentary and societal debates on assisted suicide. The motivation for the law was to provide a counterbalance to discussions on assisted suicide by improving access to palliative care and ensuring that patients’ suffering could be relieved within the healthcare system.  The law was promoted by professional associations**,** including the German Association for Palliative Care and Hospice Care, and supported by members of Parliament from multiple parties, including the Christian Democratic Party, Social Democrats, and Greens. These associations had been advocating for years to address existing gaps in palliative care and to make it an integrated part of regular healthcare.  The law’s main purpose was to establish a patients’ right to palliative care**,** to introduce reimbursement mechanisms for inpatient  consultation services and advanced care planning in nursing homes**,** and to promote the integration of palliative care across hospitals, nursing homes, and home care services**.**  Implementation of the law has been gradual. Some provisions, such as reimbursement codes for hospital consultation services and structured advanced care planning in nursing homes,  took more than five years to become operational. In practice, advanced care planning in nursing homes is not fully networked, meaning that emergency teams or other healthcare providers may not always recognize existing plans. Geographic disparities persist, with rural areas facing limited access due to distance, and non-cancer patients often experience difficulty accessing specialist services. The law also leaves open questions regarding funding and prioritization, particularly in the context of ongoing debates about assisted suicide.  Despite these challenges, the law has significantly strengthened the legal foundation for palliative care in Germany**.** Patients now have a formally recognized right to access palliative care, and hospitals and nursing homes have a structured framework for service provision.  Additional mechanisms, such as the certification of cancer centers, have reinforced access and quality standards, requiring institutions to demonstrate adequate palliative care staffing and services to maintain accreditation.  Looking to the future, concerns remain about maintaining funding and support for specialist palliative services as generalist palliative care expands. There are gaps in early access for non-cancer patients, cultural perceptions that continue to associate palliative care only with imminent death, and ongoing legislative debates about assisted suicide that may influence priorities. Nonetheless, Germany’s law provides a strong foundation**,** and continued implementation and monitoring are expected to further improve accessibility, quality, and  integration of palliative care across the country. |
| 1. What was the initial motivation for the law, who promoted it and what were its main objectives and implementation challenges? | | |
| The law on palliative care in Portugal was not the result of a broad national consensus or a structured governmental initiative. Instead, it was primarily driven by the individual initiative of a professional with experience in palliative care who held a seat in Parliament. This person belonged to a specific political party and used their position and knowledge to draft and push forward the legislation. While the law ultimately received unanimous approval in the Portuguese Parliament, it was never fully embraced as a cross-party or State priority. As the key informant explains in the interview, it remained identified, implicitly or explicitly as a law belonging to a certain political faction, rather than being seen as a national health policy shared and implemented across the political spectrum.  The motivation behind the law was clearly to give visibility, legitimacy, and legal foundation to palliative care within the national health system. It aimed to move the discipline from the margins to the core of healthcare provision, recognizing palliative care as a right and an essential part of patient support, particularly for those with life-limiting conditions.  In terms of objectives, the law intended to:   - Establish a legal framework for the integration of palliative care into the public health system. - Promote the development of palliative care services, including hospital-based teams and community care structures. - Support education and training in palliative care. - Encourage the establishment of a national commission and strategic plans to guide development.   However, the implementation challenges were and remain significant:   1. Lack of true political ownership: As mentioned, the law was not internalized as a national commitment by successive governments. It was passed, but not championed. This resulted in weak follow-up and poor enforcement. 2. Insufficient regulatory flexibility: Certain parts of the law were drafted in a way that now limits progress. For example, the law defines organizational models in rigid terms that do not easily allow for innovation or adaptation to evolving needs. What were seen at the time as “opportunities” (i.e., clear definitions and mandates) have become “obstacles” today, making it hard to reform or adapt without revising the legal text itself. 3. Absence of enforcement mechanisms: While the law includes provisions for national strategic plans (to be updated every two years), these have not been consistently developed or implemented. There are no accountability mechanisms to ensure that regional health services comply or that objectives are evaluated. 4. Weak funding provisions: The law did not come with secured or ring-fenced funding. Therefore, many of the services or initiatives it suggested were left to the discretion of local health administrations or dependent on temporary political goodwill. 5. Uneven service development: The law did encourage the creation of some national structures such as palliative care commissions, and did contribute to the development of certain services (especially hospital-based teams). However, much of the growth has been uneven across regions and not mandated. The only component of the system with close to full national coverage is hospital palliative care teams, and even those vary widely in quality and resources. 6. Training and specialization: The law did help in promoting basic education in palliative care, and some universities have introduced curricular content. However, Portugal still lacks an official medical specialization in palliative care, which severely hinders the development of expert teams and long-term leadership. 7.   In short, the law had strong symbolic and legitimizing value, but limited practical impact due to lack of political prioritization, legal rigidity, missing enforcement tools, and underfinancing. | | The law was motivated by societal and parliamentary debates on assisted suicide**,** with legislators seeking to counteract the need for assisted suicide by improving palliative care**.**  Professional associations and supportive Parliament members provided advocacy and input to shape legislation. The law aimed to promote access, identify gaps, and integrate palliative care into regular healthcare. Implementation challenges include gradual operationalization and incomplete integration across services and regions**.**  Main Objectives of the Law:  • To establish a patients’ legal right to palliative care and to improve access to palliative care  • Introduce funding mechanisms for inpatient consultation services and advanced care planning in nursing homes (increased reimbursement)  • Promote the integration of palliative care across hospitals, nursing homes, and home care.  Implementation Challenges:  • Reimbursement codes and advanced care planning provisions took over five years to operationalize.  • Advanced care planning is not fully networked; emergency services may not recognize plans.  • Access gaps remain for non-cancer patients and rural populations.  • Ongoing debates on assisted suicide may affect prioritization and funding. |
| 2. How does the law promote palliative care in your country? | | |
| The Portuguese law on palliative care does attempt to promote palliative care across several dimensions, in alignment with the WHO framework, but its impact varies greatly across components. Based on the key informant’s interview, each area can be assessed as follows:  1. Empowering Societies to Care  This aspect is weakly addressed by the law. There are no concrete provisions aimed at empowering communities or encouraging civic engagement in end-of-life care. The law does not incorporate mechanisms to raise public awareness, support families or informal caregivers, or engage civil society in a meaningful way. This limitation is one of the clearest gaps in the law's vision and implementation.  Summary: Not addressed in the law; no reference to community participation, caregiver empowerment, or public engagement.  2. Supportive Health Policies  This is where the law is strongest. Its adoption marked a significant policy milestone by legally recognizing palliative care as a patient right and part of the national health system. It introduced strategic planning obligations and promoted the creation of governance structures (like national commissions). These health policy instruments laid the foundation for service development and institutional legitimacy.  However, as the key informant emphasizes, the political will to implement the law has been inconsistent. Strategic plans were not produced every two years as mandated, and implementation has been highly dependent on local initiatives rather than national enforcement.  Summary: Strong in legal recognition and policy structure, but weak in execution and national leadership.  3. Education and Training  The law positively influenced education, particularly basic training. It helped expand curricular inclusion of palliative care in undergraduate health programs and supported the development of postgraduate offerings (diplomas and master’s). Some universities have responded with dedicated teaching units.  However, Portugal still lacks an official medical specialty in palliative care, which significantly hampers the formation of expert teams, academic leadership, and system-level development.  Summary: Moderate contribution to basic and postgraduate education; no formal specialization tracks yet.  4. Research in Palliative Care  According to the interview, research is not a focus of the law. The key informant does not mention any mechanisms, funding, or structures established to promote or support scientific research in palliative care. This absence reflects a broader systemic weakness in research infrastructure and strategy for palliative care in Portugal.  Summary: Not addressed in the law; no evident mechanisms to foster or finance research.  5. Access to Essential Medicines  This dimension is only tangentially addressed in the broader health system, but not concretely in the palliative care law. There is no specific reference in the law to ensuring access to essential palliative care medicines, such as opioids. While some general pain management policies exist in Portugal (particularly in oncology), the law does not introduce specific regulatory changes or access guarantees for opioids in palliative contexts.  The key informant does not mention this issue directly in the interview, which suggests that access to medications was not a central feature of the law's focus or its political implementation.  Summary: No explicit legal provisions regarding essential palliative medicines.  6. Development of Integrated Services  This is an area of partial advancement. The law encouraged the creation of palliative care services in hospitals and in the community. It laid out intentions for integrated provision at different levels of care. The key informant confirms that some progress was made, particularly in hospital-based teams, but emphasizes that the law only “enabled” rather than “mandated” development, leading to a fragmented and uneven implementation.  Currently, Portugal has full coverage only in hospital palliative care teams, but community-based and home teams are highly variable between regions. Integration with primary care remains limited.  Summary: Legal foundation exists, but implementation is partial and uneven. Integration remains a challenge.  Overall Evaluation: The Portuguese law on palliative care promotes development most clearly through legal recognition and supportive health policy, and to some extent, education and service creation. However, it fails to meaningfully address community empowerment, research, or access to essential medicines. Its contribution to integrated care is incomplete, limited by non-mandatory language and lack of national follow-up. | | The law promotes palliative care by establishing patients’ rights, structuring service provision, and enabling funding mechanisms. It provides legal leverage to ensure access and encourages integration of palliative care across hospitals, nursing homes, and home care.  Analyses by Aspect:  1. Empowering Societies to Care:  • Professional associations played a key role in lobbying for improvements and providing input into legislation.  • Patients’ rights to palliative care are formally recognized, strengthening advocacy opportunities.  • Outside of the law, Cancer centers need to fulfil criteria regarding the specified number of specialized palliative care professionals and beds in order to be accredited by the US and certified by the German Cancer Society. This is also one way to promote palliative care, though it’s kind of outside of the legislation.  2. Supportive Health Policies:  • The funding mechanism is addressed in the law. However, reimbursement initially underestimated workload, requiring lengthy negotiation over many years.  • Legal recognition of palliative care as a patient right supports system-wide integration.  3. Research:  • Not specifically addressed in the law.  4. Education and Training including specialization in palliative care:  • Not addressed in the law. Sub-specialty training is regulated by profesional associations.  5. Access to Essential Medicines:  • Not adressed in the law.  6. Integrated Services  • Hospital consultation teams, nursing home advanced care planning, and home care are structured and recognized by law.  • In practice, gaps remain for non-cancer patients and rural populations. |
| 3. Does the law—as it stands—secure the progress made toward ensuring patients’ right to palliative care, and if it does, could you explain how? | | |
| The Portuguese law on palliative care, although symbolically powerful and legally well-intentioned, does not effectively secure or consolidate the progress made in guaranteeing patients’ rights to palliative care. It lays a foundational legal and ethical recognition of that right but lacks the practical mechanisms necessary to ensure its realization. According to the key informant, the law’s primary contribution was to legitimize palliative care as a fundamental part of the health system. It declared that patients have the right to access palliative care, and in doing so, it offered moral and political visibility to the field. That symbolic recognition has been crucial for awareness, education, and some early service development. However, in terms of securing and sustaining progress, the law has three major limitations:  1. No Binding Enforcement Mechanisms  The law does not impose obligatory provisions for the implementation of services or care guarantees. It recommends the establishment of palliative care teams in hospitals and communities but does not require them nor penalize their absence. As a result, development has been uneven and dependent on local will, rather than guaranteed by law.  Example: While hospital-based teams are widely present, community and home-based teams are inconsistent across regions. There is no universal access in practice.  2. Lack of Sustainable Planning and Oversight  Although the law mandates that a national strategic plan be developed every two years to guide progress, this obligation has not been fulfilled. After the first plan was created, no consistent follow-up has occurred. Without planning, monitoring, or accountability, the law becomes a declaration without execution.  The key informant emphasizes that strategic plans are either not made, not implemented, or not evaluated—thus undermining any long-term progress.  3. No Dedicated Budget or Institutional Priority  The law did not establish a dedicated funding mechanism or a budget line within the national health system. Nor did it require the government to allocate specific resources. This means that progress has relied heavily on local initiatives and the dedication of individual professionals, not on systemic or State-level investment.  As the key informant notes, the law was passed without being “assumed” as a political priority by governments. It has symbolic status but not operational weight.  Does it protect what has already been achieved?  To some extent, yes, but very weakly. The law provides a legal reference point that professionals, patients, and advocates can invoke. It supports the existence of certain structures (like the national commission), and it has helped entrench palliative care within policy discourse and academic training. But in the absence of enforceable guarantees, political and institutional support for palliative care remains fragile. As the key informant puts it, “the results does not depend on our capacity alone”, they require structures, leadership, and consistent backing. The law does not offer those safeguards.  In Summary:   - The law formally recognizes the right to palliative care. - But it does not secure access or continuity of services in practice. - It lacks mandatory provisions, financing, follow-up, and policy integration. - It legitimized the field, and partially enabled development, but it fails to protect or scale up what has been achieved | | The law secures the legal right to palliative care and a framework for service delivery and funding mechanisms. Its practical impact depends on implementation, reimbursement negotiations, and integration across healthcare settings. The word “right” is not explicitly mentioned in the law text. However, in the law is stated that *“Health care includes palliative* *care of the health insured persons”* advocating the right to palliative care”. This supports the advocacy of palliative care in Germany by allowing the health care professionals and patients (along with their families) demand the access to and development of palliative care services.  Main Limitations of the Law:  • Funding allocation (reimbursement initially underestimated workload, requiring lengthy negotiation).  • Neither undergraduate nor specialized palliative care training is not recognized in the law.  • Volunteer services should be better acknowledged and reimbursed  • Access to medications (such as opioids) is not addressed in the law. |
| Additional aspects and remarks | | |
| In addition to the themes explicitly addressed in the following first three questions, motivation, promotion of palliative care, and the law’s capacity to secure rights, the interview reveals several important, often overlooked dimensions of the Portuguese palliative care landscape that deserve attention:  1. The Consequences of Over-Specification in the Law  One critical insight from the key informant is that the law’s rigidity has become counterproductive. Some of its initial formulations intended to clarify and strengthen implementation, have ended up limiting innovation.  For example, the law defines certain models of service organization in a fixed way, which today prevents the adoption of newer, more flexible, and potentially more effective configurations of care.  This highlights an important legal-design lesson: over-specification can hinder adaptability, especially in a field like palliative care that evolves quickly with demographic, clinical, and technological shifts.  2. The Political Life of the Law: From Parliament to Neglect  Another major theme is the political sociology of the law: The key informant explains that although the law was approved unanimously, it was never truly owned by all parties. Because it was associated with a single person and a specific political party, it was not embedded as a national cross-party health priority.  As a result, subsequent governments did not implement it seriously or consistently, and the law lacked long-term political sponsorship.  This offers a cautionary tale about how even well-drafted legislation can fail when it lacks sustained political commitment and institutional ownership.  3. The Structural Invisibility of Palliative Care in the Health System  The key informant points out that despite the law’s existence, palliative care still lacks systemic integration in Portugal. It is not treated as a mainstream or priority area within the public health system. This structural invisibility means:   - Lack of strategic leadership from the Ministry of Health - Absence of integration into general healthcare planning - Minimal leverage to influence resource distribution   This reflects a broader cultural and institutional neglect, where palliative care remains peripheral, despite legal recognition.  4. The Limitations of Volunteerism and Professional Commitment  The key informant underscores that much of the progress made in Portugal has come not because of the law, but in spite of it, driven by local actors, professionals, and volunteers. While commendable, this dynamic is inherently fragile.  The country cannot depend indefinitely on professional dedication and moral commitment. As the key informant warns, "we cannot rely on heroism.”  This underscores the need for systemic solutions, beyond the goodwill of individuals.  5. Missed Opportunity for Intersectoral Collaboration  Though not elaborated extensively, there’s an implied criticism that the law did not capitalize on opportunities to align with other sectors—such as social services, education, or community health.  For example, there is no mention of linkages between palliative care and long-term care, or of collaboration with civil society organizations.  This absence contributes to the fragmented development of palliative care, particularly in community and home settings.  6. Lack of Real-Time Evaluation and Course Correction  The law mandates the creation of strategic plans every two years, but, as the key informant notes, these have not been produced or updated. More critically, no formal evaluation mechanisms have been embedded into the law.  This reflects a broader failure in policy learning and accountability. Without evaluation, implementation is not only weak, it is directionless. | | The future of palliative care in Germany depends on sustained implementation, monitoring, and adaptation to societal and legislative changes (including regulations regarding assisted suicide). While integration is advanced, attention is needed to maintain support for specialist services, expand access to non-cancer patients, and address rural disparities. Public perception and ongoing debates about assisted suicide may influence service priorities, requiring continued advocacy and awareness campaigns.  Details:  • Ensuring sufficient funding and support for specialist palliative care units alongside generalist care.  • Improving access for non-cancer patients and underserved populations in rural areas.  • Expanding networking and recognition of advanced care plans across healthcare services.  • Continuing certification and quality standards in institutions like cancer centers to reinforce service quality.  • Monitoring and addressing ongoing debates on assisted suicide to safeguard resources and priorities for palliative care.  • Public perception often associates palliative care with imminent death as a stigma,limiting early access. Thus, the there is still a need to actively promote palliative care and improve public awareness.  • Cancer centers need to fulfil criteria regarding the specified number of specialized palliative care professionals and beds in order to be acrredited by the US and certified by the German Cencer Society. This is also one way to promote palliative care and improve access to palliative care. |

# **Supplementary Table S10** Summary of Interviews with Key Informants of Armenia and Albania.

| Armenia | | Albania |
| --- | --- | --- |
| Time | *August 20, 2025* | *July 14, 2025* |
| Interviewer | *TC* | *TC* |
| Interviewee | *AP, AT* | *IL* |
| Executive Summary | | |
| Armenia does not have a standalone palliative care law. Palliative care is regulated through amendments in the general medical law (“Medical Care and Services to the Population”), complemented by ministerial orders, standards, clinical guidelines, and licensing  procedures. The initial introduction of palliative care was prompted by a needs assessment conducted by Dr. Stephen Connor, which identified that approximately 5,500 cancer patients require palliative care and of them 70–80% require pain management. The assessment also highlighted major gaps in infrastructure, specialized staff, access to medicines, and professional training.  The Open Society Foundations (International Palliative Care Initiative) led advocacy efforts with The Ministry of Health to support the need for development of palliative care and  to support the inclusion of a legal definition of palliative care in the general health law, recognition of palliative care as a specialty/subspecialty, and development of standards,  licensing procedures, and opioid prescription guidelines.  The Armenian legal framework, enacted in 2017, provides comprehensive regulations for palliative care, ensuring that services can legally operate and integrate into primary healthcare. However, implementation challenges remain significant: there is a very limited number of trained professionals, particularly doctors, nurses, psychologists, and social workers; short-term training courses are insufficient to create specialists; oversight mechanisms are weak, focusing primarily on infrastructure rather than service quality; andcoordination between ministries is fragmented. Cultural and societal norms limit public awareness and acceptance of palliative care, and funding is heavily dependent on external donors, which reduces sustainability. In addition, public awareness about palliative care has remained very low and as well as the awareness of the need of palliative care among health care professionals.  Despite these challenges, the palliative care legislation/legislative amendment itself in the general health law establishes the legal right to palliative care, sets standards, and enables structured service delivery. Its effective implementation is essential to translate legal guarantees into practical access, quality care, and integration into the healthcare system. Civil society currently plays a critical role in mentoring, monitoring, and supporting service provision, but a systematic government-led approach is still lacking. The law lays a strong foundation, but practical barriers in staffing, training, funding, oversight, and public awareness, limit its full impact. | | The Albanian Palliative Care Law was introduced in 2014 with the aim of ensuring that all patients with incurable diseases, including those with cancer and non-cancer conditions, have access to palliative care services. The law was the result of the efforts of the Albanian Palliative Care Association, formed in 2002, and supported by international experts. The law outlines the provision of palliative care in public hospitals, particularly in regional hospitals, and mandates primary healthcare providers to offer basic palliative care services.  Despite the law’s strong principles, its implementation has faced significant challenges. The law is well-structured on paper but has faced gaps in practical application due to systemic issues within the healthcare system. The major obstacles to successful implementation include insufficient education and training for healthcare providers, especially in primary healthcare, resistance from healthcare workers who lack understanding of palliative care, and the underutilization of palliative care units established in regional hospitals. Furthermore, the availability of essential medications, particularly opioids, is restricted due to legal and procedural barriers, leaving many patients in pain and without adequate care.  While the law has successfully promoted palliative care for cancer patients in certain regions, its application to non-cancer patients remains limited, and the services available are not well integrated into the primary healthcare system. Additionally, the public and healthcare workers remain largely unaware of the law’s provisions, hindering its full potential. The law has, however, provided a solid foundation upon which further advocacy, education, and policy development can be built to ensure the rights of all patients to receive palliative care.  The interviewee stressed the importance of greater integration of palliative care into medicaland nursing curricula, as well as the need for continued collaboration with international experts and organizations. These efforts, coupled with stronger community awareness and education, are essential to advancing palliative care in Albania and ensuring that all patients, regardless of their condition or location, can access the care they need. |
| 1. What was the initial motivation for the law, who promoted it and what were its main objectives and implementation challenges? | | |
| The initial motivation for introducing palliative care in Armenia came from a needs assessment that identified significant gaps in services, infrastructure, and pain management for cancer patients. The initiative was led by the Open Society Foundations, with later advocacy and support from the Ministry of Health. The objective was to formalize palliative care in legislation, define standards, and create legal and administrative pathways for service provision. Implementation has been challenged by limited trained staff, insufficient oversight, fragmented coordination, and cultural and funding barriers.  Main Objectives of the Law:  • Introduce a legal definition of palliative care in the general health law.  • Recognize palliative care as a specialty/subspecialty.  • Establish standards, clinical guidelines, and licensing procedures.  • Enable opioid prescription and circulation for medical purposes.  • Integrate palliative care into primary healthcare, while also allowing outpatient, inpatient and private licensed services.  Implementation Challenges of the Law:  • Human resources: Few trained doctors, nurses, psychologists, and social workers; low motivation due to low salaries and limited training.  • Education: Short courses (two-week or limited-duration) insufficient to train specialists.  • Oversight: No dedicated mechanisms for monitoring service quality; licensing focuses on infrastructure.  • Coordination: Ministries (Health, Social Affairs) and agencies work separately, causing fragmented services.  • Public awareness: Cultural reluctance to discuss death and end-of-life care.  • Funding: Heavy reliance on external donors; government funding limited, particularly for outpatient services.  • Regulatory challenges: Police monitoring of opioids due to societal drug control concerns sometimes restricts prescription. | | The Albanian Palliative Care Law was initially motivated by the need to address the inadequate palliative care services for patients with incurable diseases in Albania. The promotion of the law stemmed from the efforts of palliative care providers who, in 2002, formed the Albanian Palliative Care Association in order to better promote and develop palliative care in Albania. International support from the US played a crucial role with assistance in drafting the law.  The primary objective of the law, enacted in 2014, was to ensure that palliative care services would be available for all patients with incurable diseases, regardless of whether they had cancer. However, significant challenges have emerged in the law’s implementation. While the law was passed in 2014, its actual promotion has been limited, and palliative care services are still primarily focused on patients with cancer. One of the law's objectives was to establish palliative care units in public regional hospitals, but these units are underutilized and often do not provide comprehensive palliative care due to a lack of trained healthcare providers and resources.  Main Objectives of the Law:  • Universal Access and Right to Receive Palliative Care: To protect the right to receive and to provide palliative care for all patients with incurable diseases, including both cancer and non-cancer patients.  • Integration into Public Healthcare System: To ensure that palliative care services are incorporated into public hospitals, especially regional hospitals, as well as primary  healthcare settings.  • Pain Management and Symptom Relief: To ensure that patients receive adequate pain management, including access to opioids and other necessary medications, for  symptom relief.  • Multidisciplinary Approach: To promote a holistic, multidisciplinary approach to care, involving physicians, nurses, social workers, and psychologists in the  management of patients.  • Education and Training: To mandate training for healthcare professionals to ensure they are equipped with the knowledge and skills to deliver palliative care effectively.  Implementation Challenges of the Law:  • Limited Healthcare Provider Education: Lack of sufficient education and training for healthcare workers, particularly in primary healthcare settings, regarding palliative care practices and opioid prescriptions.  • Focus on Cancer Patients: Despite the law's intention to serve all patients with incurable diseases, palliative care remains primarily focused on cancer patients, with limited services available for patients with other chronic, incurable conditions.  • Inadequate Integration into Primary Healthcare: The law’s provisions for integrating palliative care into primary healthcare have not been fully realized, leaving many patients without access to necessary services in rural or underserved areas.  • Underutilized Palliative Care Units: Palliative care units established in regional hospitals are often underutilized or not functioning properly due to a lack of trained staff and resources.  • Resistance from Healthcare Providers: Many healthcare workers, particularly in primary care, are unfamiliar with or resistant to palliative care principles, preventing  effective implementation.  • Access to Pain Management Medications: Restrictions on the prescription of opioids and other pain management medications, especially by primary healthcare providers, limit access for patients in need of adequate pain control. |
| 2. How does the law promote palliative care in your country? | | |
| The law provides a regulatory and legal framework, enabling palliative care services to exist, operate legally, and integrate into the healthcare system. It standardizes practices, provides licensing pathways, and allows for opioid use in pain management. However, promotion of services relies heavily on civil society, as implementation gaps and bureaucratic and cultural barriers limit widespread access.  Analyses by Aspect:  1. Empowering Societies to Care:  Civil society organizations mentor and monitor services.  • Public campaigns raise awareness, but societal norms limit open discussion about end-of-life care.  2. Supportive Health Policies:  • Law and ministerial orders provide regulations, standards, and guidelines.  • Licensing enables institutions to legally deliver palliative care services.  3. Research:  • Research is not explicitly mentioned in the law text, however, in the law text, one of the principles of palliative care is mentioned as follows: Provide palliative care from the early stages of the disease in conjunction with life-prolonging medical care (chemotherapy, radiotherapy, etc.), conducting the research necessary to understand and manage severe clinical complications.  • No systematic monitoring or evaluation of service quality currently exists in Armenia.  4. Education and Training:  • Short-term courses exist for doctors, nurses, psychologists, and social workers.  • Limited participation due to cost, insufficient depth, and lack of incentives.  • Plans exist to introduce elective medical student courses in palliative care.  • The law itself highlights the need for specialization and ongoing education in palliative care. Undergraduate training is not mentioned in the law text.  5. Access to Essential Medicines:  • Opioid prescription is legally permitted.  • Police monitoring, due to societal drug issues, occasionally limits access.  • Palliative sedation is not mentioned in the law text.  6. Integrated Services:  • Palliative care integrated into primary healthcare.  • Licensed inpatient facilities and private services exist, but quality and supervision vary.  • Coordination gaps between ministries and agencies create fragmented service delivery.  • Pediatric palliative care is also highlighted in the law text. | | The law was designed to promote palliative care by mandating its integration into the healthcare system, particularly within public hospitals and primary healthcare settings. It established the requirement for palliative care services to be available in regional hospitals and for primary healthcare providers to offer basic palliative care to patients with incurable diseases. Despite these provisions, the promotion and implementation of palliative care have been limited in practice.  Healthcare workers, particularly in primary care, often reject the concept of palliative care or do not have the necessary education and training to provide it. The lack of basic palliative care knowledge among primary healthcare workers is a huge barrier, preventing the law from reaching its full potential. Additionally, while palliative care units have been established in public hospitals, they often do not function effectively. In many cases, these units are just nominally labelled as “palliative care” but lack the resources, specialized staff, and training to provide high-quality palliative care services. In addition, palliative is mainly focused on patients with cancer, but not for patients with non-malignant incurable diseases often go without appropriate care. In addition, even though the law itself is  well-written and comprehensive, the population and health care providers in Albania are not well aware of its existence.  Empowering Societies to Care:  • The law encourages a more community-based approach to palliative care by recognizing that care should not only come from healthcare institutions but also from families and local communities.  • However, there is a lack of awareness and understanding among the general population about palliative care and their rights under the law, which undermines its potential. Empowering communities to advocate for palliative care is crucial but has not been fully addressed in the law’s implementation, particularly in rural areas.  Supportive Health Policies:  • The law includes provisions to integrate palliative care into the national healthcare system and regional hospitals. However, supportive health policies to promote the  law's provisions have been weak.  • The lack of proper infrastructure, such as trained healthcare workers and adequate resources in regional hospitals, limits the success of these health policies.• Furthermore, while the law provides the legal basis for palliative care, its actual enforcement and the establishment of relevant policies at local levels are lacking.  Research:  • The law does not specifically mandate or encourage research into palliative care, which is a critical aspect for understanding patient needs, improving practices, and evaluating outcomes.  • There is an absence of data-driven policies or support for research into palliative care, leaving gaps in evidence-based practices.  • Given the emerging nature of palliative care in Albania, establishing research frameworks would help refine the care provided and evaluate the law’s impact.  Education and Training:  • The law includes provisions for the training of healthcare providers in palliative care; however, the actual implementation of these training programs has been limited.  • Primary healthcare workers, who are the first point of contact for most patients, often lack the basic knowledge and skills required for providing palliative care.  • There is a pressing need to integrate palliative care education into medical and nursing school curricula to ensure that future healthcare workers are well-prepared.  Access to Essential Medicines:  • The law ensures that pain management, including the use of opioids, is available for all patients, but access to essential medicines remains a major challenge in practice.  • Legal and procedural barriers prevent primary healthcare providers from prescribing opioids, and in some regions, there is a shortage of oncologists who can prescribe them.  • This restriction affects the ability of non-cancer patients to receive proper pain management, leaving many without adequate care.  Integrated Services:  • The law envisions the integration of palliative care into both primary healthcare and specialized hospital services, ensuring that patients with incurable diseases receive  comprehensive care.  • However, integration has been slow and inconsistent. While some palliative care units have been established in hospitals, they are often underutilized or lack sufficient  staffing and resources.  • There is also a lack of collaboration between primary healthcare providers, hospitals, and other healthcare institutions, which hinders the development of a truly integrated system of care for patients in need of palliative services |
| 3. Does the law—as it stands—secure the progress made toward ensuring patients’ right to palliative care, and if it does, could you explain how? | | |
| Legally, the law/amendment in the general health law provides standards for service delivery. The law does not explicitly highlight the right to palliative care, however it is explicitly mentioned in the law text that *Palliative medical care and services (hereinafter referred to as* *Palliative Medical Care) are provided to any person with a life-threatening or ongoing and/or life-limiting illness.*  In practice, progress is limited by implementation challenges, such as lack of trained professionals, insufficient oversight, fragmented coordination, cultural barriers, and reliance on civil society and external funding. Without improved support, supervision, and funding, patients’ access and quality of palliative care remain inconsistent.  Main Limitations of the Law:  • Weak quality monitoring and oversight mechanisms.  • Insufficient trained professionals (physicians, nurses, psychologists, social workers).  • Fragmented coordination between ministries and agencies.  • Heavy reliance on external donors, limited internal government funding.  • Cultural and societal barriers limit public understanding and acceptance of palliative care.  • Licensing focuses on infrastructure, not quality or clinical outcomes. | | Although the law is well-written and guarantees patients’ rights to palliative care, its practical application and enforcement remain a major challenge. The law explicitly states thatpalliative care should be available to all patients with incurable diseases, including patients with non-malignant diseases, and that it is a human right. However, in reality, palliative care services are largely unavailable to these patients.  The primary issue lies in the gap between the law’s theoretical provisions and its practical implementation. Healthcare workers, particularly in primary care settings, lack awareness and education about palliative care, which limits their ability to provide appropriate care. Moreover, opioids, which are essential for pain management, are not easily accessible to many patients, especially those without cancer, due to restrictions on prescription practices. Despite the law's strong emphasis on the availability of palliative care services and pain management, the reality is that patients outside of major urban centers or those without cancer often receive inadequate or no care at all.  The law also emphasizes the importance of education for healthcare professionals, yet there is a lack of integration of palliative care into medical curricula. Palliative care is not a mandatory subject in either medical or nursing schools, which means that healthcare providers are not trained to recognize and address the needs of patients requiring palliative care. The law's promotion relies heavily on advocacy and education to ensure its success, but these efforts have not been fully realized.  Limitations of the Law  • Lack of Practical Implementation  While the law guarantees the right to palliative care, its widespread application is not achieved. The law does not explicitly address practical guidance to foster implementation across different regions in Albania.  • Inadequate Healthcare Worker Education  The law emphasizes the importance of education for healthcare professionals, yet it does not provide specific mandates or clear guidelines on how education should be structured or integrated into healthcare training. The law calls for healthcare providers to be educated about palliative care, but it does not specify the mechanisms for ensuring that this education is effectively implemented, especially in primary healthcare.  • Limited Access to Pain Management  The law addresses the importance of pain management, including the provision of opioids, and guarantees access to pain relief for all patients with incurable diseases.  • Geographical Disparities  The law mandates that palliative care be available across the country, but it does not explicitly address the geographical disparities in service provision.  • Absence of Mandatory Education  The law mentions the importance of healthcare worker education but does not require that palliative care be included as a mandatory subject in medical or nursing school curricula. This gap in mandatory education provisions can be one barrier in establishing comprehensive educational programs.  • Inconsistent Enforcement and Promotion  The law relies on advocacy and education for its promotion, but these efforts are not mandated or sufficiently emphasized in the law itself. |
| Additional aspects and remarks | | |
| The future of palliative care in Armenia depends on the successful implementation of planned reforms, particularly the integration into universal health coverage, expansion of education and training, and improved oversight. While civil society continues to play a key role, systemic government support, sustainable funding, and cultural adaptation are essential for long-term progress.  Details:  • Integration into Universal Health Coverage: Palliative care services are planned to be included, which could improve funding, access, and quality assurance mechanisms.  • Education and Training: Elective courses in palliative care will be offered to medical students, with potential for mandatory inclusion in the future. Social work students already receive more comprehensive training than medical students.  • Human Resources Development: Increasing the number of trained doctors, nurses, psychologists, and social workers is a priority to ensure service coverage.  • Monitoring and Quality Assurance: Inclusion in universal health coverage may establish formal oversight mechanisms to monitor service quality, which are currently lacking.  • Civil Society Support: Mentorship, guidance, and advocacy from civil society organizations remain critical due to limited government-led implementation and funding.  • Challenges Ahead: Cultural barriers, public awareness, fragmented ministry coordination, and reliance on external funding continue to pose challenges. Sustainability depends on systemic governmental support and structured implementation. | | While the law itself, enacted in 2014, is a significant step forward in recognizing palliative care as a human right, its effectiveness depends on education, advocacy, and practical integration into the healthcare system. There is a critical need to integrate palliative care education into medical and nursing faculties, as well as to improve public awareness about the law and its provisions. The public should be informed about their right to palliative care so that they can advocate for themselves and demand services from primary healthcare providers. Increased collaboration with international experts and advocacy organizations, such as the Albanian Palliative Care Association and the European Association for Palliative Medicine could help improve the situation by fostering stronger relationships with the Ministry of Health and the Ministry of Education. These collaborations could encourage the inclusion of palliative care training in university curricula, which would be an essential step toward better implementation.  In conclusion, while Albania's Palliative Care Law represents an important milestone in the recognition of patients’ rights to palliative care, its implementation remains a work in progress. The law has faced significant practical challenges, including limited healthcare provider training, inadequate integration into primary healthcare, and insufficient public awareness. To ensure that the law fully promotes palliative care for all patients in need, extensive efforts in education, advocacy, and system integration are necessary. The next steps should focus on improving healthcare professionals' understanding of palliative care, raising public awareness, and ensuring that palliative care services are accessible throughout the country, not just for cancer patients or in urban áreas. |

# **Supplementary Table S11** Summary of Interviews with Key Informants of Austria and Greece.

| Austria | | Greece |
| --- | --- | --- |
| Time | *July 16, 2025* | *July 14, 2025* |
| Interviewer | *LM* | *CC* |
| Interviewee | *RS* | *AT* |
| Executive Summary | | |
| Austria’s national hospice and palliative care law, enacted in 2022 after more than two decades of advocacy, marks a significant step forward in securing sustainable funding for specialized palliative and hospice services. Unlike other models, the Austrian law is not rights-based but funding-oriented, aiming to guarantee equitable access through financial support and quality standards. This strategic choice followed years of deliberation among stakeholders, who concluded that without dedicated financing, legal entitlements would be ineffective.  The law has increased visibility for palliative care in both public and professional spheres and strengthened the health system’s commitment to the field. It establishes clear quality criteria linked to funding, requiring minimum staffing levels and defined education standards for professionals. Importantly, the legislation also extends to paediatric palliative care and the volunteer hospice sector, recognizing the structural importance of volunteers.  Despite these achievements, the implementation phase has revealed significant challenges. The main difficulty lies in meeting the quality standards amid workforce shortages, especially for nurses and doctors. The COVID-19 pandemic exacerbated these problems, making it difficult for services to comply without compromising patient care.  Furthermore, the law excludes general hospitals from these funding streams. Palliative care units are financed through the performance-based hospital financing system commonly used in Austria. If financial resources are available, projects in nursing homes and home care projects in the field of hospice and palliative care can also be financed.  Overall, the Austrian law ensures financial stability for key palliative care structures and improves quality, but it does not enshrine palliative care as a patient right. Future progress will depend on maintaining political commitment, addressing workforce shortages, and possibly complementing this financial framework with rights-based guarantees.  The law’s primary motivation was to secure public funding for specialized palliative and hospice care services after decades of advocacy.  It was promoted by Austria’s two main hospice and palliative care organizations in partnership with the Austrian National Public Health Institute and the Ministry of Health.  Funding is conditional on meeting specific quality criteria related to staffing and professional training, which a fact that has created implementation challenges given due to workforce shortages.  The law covers paediatric palliative care and volunteer coordination, emphasizing a holistic approach to hospice services.  It does not guarantee a patient’s legal right to palliative care, focusing instead on financing and quality of care improvement. | | The detailed one-by-one responds to the questions are provided below. |
| 1. What was the initial motivation for the law, who promoted it and what were its main objectives and implementation challenges? | | |
| The Austrian palliative care law emerged from a long-standing need for sustainable funding. According to Rainer Simader, the process began in 2001 and culminated in the adoption of the law in 2022. Its initial motivation was to ensure that palliative care would be accessible and affordable to all citizens, with services available across all regions of Austria. This was not merely a financial issue but also a structural one: without guaranteed funding, service development was inconsistent and vulnerable to political change.  The law was promoted by Austria’s two umbrella organizations for hospice and palliative care, working closely with the Austrian National Public Health Institute, an entity under the Ministry of Health and Social Affairs. Early advocacy efforts included wide consultations with stakeholders, from religious organizations to private entities, culminating in Austria’s first parliamentary hearing on end-of-life care in 2001. This long process reflected both the complexity of building consensus and the sensitivity of the topic.  In terms of objectives, the law intended to:  Secure permanent public funding for specialized palliative care services across all Austrian regions.  Ensure quality of care by linking financial support to compliance with national quality criteria, including staffing ratios and training requirements.  Include paediatric palliative care in the national funding scheme, recognizing its specific needs.  Integrate volunteer support structures into the financing model, acknowledging their critical role in palliative and hospice care.  Implementation challenges have been considerable. The law links funding to compliance with strict quality criteria, which define minimum staffing ratios and educational requirements for professionals. While this approach aims to enhance service quality, it has placed considerable pressure on providers. Workforce shortages, especially among nurses and doctors, have made it difficult for services to meet these criteria. The COVID-19 pandemic worsened these constraints, creating uncertainty for organizations that risk losing funding if standards are not met. Although the law includes provisions for education financing, many services struggle to release staff for training without jeopardizing care capacity.  1.Compliance with quality standards: Services must meet strict staffing and training requirements to qualify for funding, which is difficult amid workforce shortages.  2.Staff shortages: A lack of nurses and doctors, aggravated by the COVID-19 pandemic, makes it hard to meet criteria without compromising patient care.  3.Operational constraints: While the law funds education, services cannot easily release staff for training without reducing care capacity. Particularly in the case of part-time employees, it is difficult to cover training periods.  4.Risk of financial insecurity: Organizations failing to meet standards face loss of funding, creating anxiety in an already strained sector.  5.Limited scope: The law applies only to specialized services (hospices, hospice day centers, volunteer teams, mobile palliative care teams and hospital-based palliative care consultation services), not to palliative care units in hospitals or to projects involving hospice and palliative care in primary care (nursing homes and mobile nursing and care). | | The key informant explained that the first law that mentioned palliative care dated back to 2003 and a relevant ministerial decree was issued in 2007 but unfortunately was never implemented due to very demanding prerequisites. Thereafter a small group of committed professionals, recognizing the increasing needs of patients with advanced illness, began advocating for a legal framework that would support the systematic development of palliative care.  Their efforts aimed to secure not only service provision but also to enable progress in education and research in this field. A draft of a comprehensive law for palliative care was prepared by the National Committee for Palliative Care, adopted by the Ministry of Health and forwarded to Parliament, where there were tensions since some members, particularly from the opposition, expressed concern that the law might favor the privatization of healthcare.  Despite these challenges, a version of the law was finally passed in 2022. However, several key elements of implementation still depend on additional decrees, both ministerial and presidential, which are currently in preparation. The key informant emphasized that the law was a necessary milestone to legitimize palliative care in Greece and to initiate a broader policy discussion. |
| 2. How does the law promote palliative care in your country? | | |
| The Austrian law primarily promotes palliative care through a robust funding mechanism. Resources are allocated exclusively to specialized services, including hospices, hospice day centers, volunteer teams, mobile palliative care teams (affiliated with both hospitals and nursing homes), and hospital-based palliative care consultation services. This financial commitment has increased the availability of specialized services and, by linking funding to quality standards, has contributed to improving the competence of professionals. Simader notes that public awareness of palliative care has also grown significantly, as the law’s adoption attracted widespread media attention and placed the issue firmly on the political agenda.  A distinctive feature of the Austrian approach is its inclusivity: the law explicitly includes covers paediatric palliative care and recognizes the crucial role of volunteers by funding their organizational support and the professional staff who coordinate volunteer teams. This reflects a holistic vision of palliative care that integrates clinical expertise with community involvement.  However, the law does not apply to palliative care units located in general hospitals or to nursing homes and mobile care providers offering hospice and palliative care as part of basic care, meaning that its scope is limited to specialized services. While these institutions have indirectly benefited through increased training and awareness, the absence of direct financial support for general settings leaves gaps in comprehensive coverage.  1. Empowering Societies to Care:  This aspect is not a core focus of the law. The legislation does not include explicit measures to empower families or communities to provide care at the end of life. However, a distinctive feature is the recognition and funding of volunteer hospice structures, which are formally supported under the law. Professional coordinators of volunteer teams, as well as basic education for volunteers, are financed, reinforcing the structural role of volunteers in Austria’s palliative care system.  2. Supportive Health Policies:  The law represents a major policy milestone by securing public funding for specialized palliative care services at the national level. It clearly establishes financial obligations for the State, federal states and national insurance and ties funding to compliance with quality criteria, including staff-to-patient ratios and minimum educational standards. This linkage aims to enhance both access and quality of care. By providing stable financing, the law also elevated palliative care within Austria’s health policy priorities and increased its visibility in the public discourse.  3. Education and training are explicitly addressed in the law’s quality criteria. To qualify for funding, services must demonstrate that nurses, physicians and social workers meet required levels of training in palliative care. For many other professionals the quality criteria recommend both FTEs and level of education. The law also allocates funds for education. However, practical challenges persist: workforce shortages make it difficult to release staff for training without reducing patient care capacity. The training of coordinators and basic education for volunteers is also supported.  4. Research in Palliative Care:  The law does not mention research promotion or funding.  5. Access to Essential Medicines:  Not specifically included in the law.  6. Development of Integrated Services:  The law significantly strengthens specialized palliative care by funding hospices, hospice day centers, volunteer services, mobile palliative care teams, and hospital-based palliative care consultation services. These specialized services benefit directly from the financing model, which also imposes minimum quality standards. However, the law does not extend funding to general hospitals, nursing homes, mobile care, and support or facilities for people with disabilities. While awareness and training in these settings have improved indirectly, integration across all levels of care remains incomplete. | | According to the key informant, the law plays a critical role in promoting the development of palliative care by formally recognizing it as a component of the national health system. It establishes the legitimacy of palliative care services and brings long-overdue visibility to a field that was previously fragmented and largely invisible in public health debates.  The law outlines a national framework for the organization of services, the establishment of interdisciplinary teams, and the promotion of home-based care alongside hospital services. It also incorporates provisions for professional education and training, as well as for research. A national strategic plan has already been drafted and approved by the High Medical Council, and a presidential decree is currently under review, marking a concrete step toward implementation.  The key informant stressed the importance of clearly distinguishing palliative care from existing pain clinics, noting that the latter are typically led by anesthesiologists and lack the holistic, interdisciplinary approach that characterizes true palliative care. The law also opens the door to academic recognition, by enabling palliative care to be developed within medical schools and university-based services. |
| 3. Does the law—as it stands—secure the progress made toward ensuring patients’ right to palliative care, and if it does, could you explain how? | | |
| The law does not secure a legal right to palliative care. As Simader emphasizes, the legislation was designed to address the most pressing obstacle, funding, rather than creating an enforceable entitlement. This was a deliberate strategic choice, probably based on the assumption that sustainable financing would lead to expanded services and better access.  While the law has succeeded in creating a strong financial framework and incentivizing quality improvements, it does not guarantee universal coverage. Patients cannot claim palliative care as a legal right, and access depends on the continued development of services and availability of resources. In Simader’s words, “It’s not the law which ensures patients’ rights because it’s a law which ensures the funding.”  For now, the law provides stability for existing services and a foundation for growth, but its impact on guaranteeing patients’ rights remains indirect and incomplete.  Major limitations of the law include:  1.No legal right for patients: The law does not establish palliative care as an enforceable entitlement; it is entirely funding-oriented.  2.Dependence on compliance with quality standards: Services that fail to meet staffing and training requirements risk losing funding, which may reduce availability.  3.Limited scope: The law only finances specialized services (hospices, day hospices, volunteer services, and mobile palliative care teams, as well as hospital-based palliative care consultation services) and excludes general hospitals and nursing homes.  4.Workforce shortages: Deficits in nurses and physicians hinder compliance with quality criteria, creating uncertainty for providers and patients. | | In the key informant’s view, the law is an essential step toward ensuring the right to palliative care, particularly because it provides a legal foundation for service development and governmental responsibility. However, she cautioned that real progress depends on follow-up actions, especially the implementation of the strategic plan and the formal recognition of palliative care as a medical subspecialty.  The key informant noted that while some palliative services are technically included under primary care laws, they are insufficient in scope and structure. Establishing dedicated palliative care teams with specialized training and interdisciplinary composition remains a major challenge. The key informant emphasized the importance of academic development: training future professionals, building clinical expertise through hospital-based services, and supporting research. A long-term vision would include integrating palliative care into university curricula, offering structured clinical rotations, and building pathways for specialization or competency-based credentials.  The key informant concluded by expressing optimism that the recent advances such as the national strategy and pending presidential decree, could be transformative if sustained political and professional support is maintained. |
| Additional aspects and remarks | | |
| Inclusion of Paediatric Palliative Care:  A distinctive feature of the Austrian law is that it explicitly includes paediatric palliative care services within its scope. This ensures that children with life-limiting conditions, as well as their families, are recognized in the funding framework, an area often neglected in other countries.  Recognition and Funding of Volunteer Structures:  The law acknowledges the crucial role of hospice volunteers by providing public funding for the professional coordinators who manage volunteer teams. Volunteers are regarded as a key structural pillar of Austria’s palliative care system, and this provision reinforces their long-term sustainability. The law also finances the formal training required for volunteers themselves (at least 80-hour basic hospice volunteer education plus 40-hour placement).  Quality Criteria as a Double-Edged Sword:  While quality standards linked to funding aim to enhance care, they have also become a major source of anxiety for providers. Organizations risk losing essential funds if they fail to meet staffing ratios or educational requirements, a challenge made more acute by nationwide shortages of nurses and doctors. This highlights the tension between quality assurance and feasibility in resource-constrained environments.  Historical Strategic Choice: Funding over Rights  A particularly relevant insight is the strategic decision made during the legislative process: stakeholders had to choose between a rights-based law and a funding-based law. They opted for the latter, prioritizing financial sustainability over legal entitlement. While this approach improved service provision, it left open the question of whether Austria should eventually complement this model with enforceable patient rights. A purely performance-based billing system was firmly rejected to avoid the disadvantages of this form of financing.  Impact of Workforce Shortages and Demographic Change:  Simader stressed that the system is under growing pressure due to demographic trends, with increasing numbers of older people requiring care. Combined with structural shortages of nurses and physicians, this trend poses a long-term risk to implementation, even with secured funding.  COVID-19 Timing and Implementation Stress:  The law was enacted and began implementation during the COVID-19 pandemic. Workforce disruptions and care delivery challenges during this period exacerbated difficulties in meeting the quality standards tied to funding, making rollout particularly complex. | | None. |

# **Supplementary Table S12** Summary of Interviews with Key Informants of Romania and Israel.

| Romania | | Israel |
| --- | --- | --- |
| Time | *July 23, 2025* | *October 1, 2025* |
| Interviewer | *CC* | *TC* |
| Interviewee | *DM* | *RS* |
| Executive Summary | | |
| In Romania, the legislative framework includes parliamentary laws, government decisions, and ministerial orders. While parliamentary laws set the general legal framework, in many technical and sector-specific áreas, particularly in health care, **ministerial orders** (ordine ministeriale) are the most relevant and widely used instruments. They allow for rapid regulatory adaptation without undergoing the lengthy parliamentary process.  As a result, unlike in systems where a parliamentary act is the primary reference, in fields such as health and palliative care in Romania, day-to-day practice is effectively regulated by ministerial orders. This explains why, in the case of palliative care, there is no dedicated “palliative care law” passed by Parliament; instead, the main regulatory instrument is a **Ministerial Order** issued by the Ministry of Health, which serves as the primary framework governing the organisation, standards. | | Israel’s palliative care framework is built on universal healthcare coverage, ensuring that every citizen is insured through one of four Health Maintenance Organizations (HMOs). The foundation for palliative care legislation began with the Dying Patient Act of 2005, initiated by the Israeli Medical Association, Members of Parliament, and the National Ethics Committee.  The motivation for the Act, enacted in 2005 was to define who qualifies as a dying patient and to formally recognize their rights, though the right to palliative care is not explicitly addressed in the 2005 law text. Yet, 20 years ago, the access to palliative care was poor nationwide in Israel.  Following the law enacted in 2005, the 2009 palliative care directive was enacted by the Ministry of Health. The directive guarantees that any patient officially recognized as end-of-life is entitled to 24/7 palliative care, either at home or in an institution, with a multidisciplinary team including a physician, nurse, and social worker. Care is fully covered by the national healthcare system, ensuring no out-of-pocket costs. Implementation in the community has been highly successful, particularly for home hospice,  due to the ability of private-public partnerships to deliver services efficiently. However, hospital-based has lagged, with only less than half of hospitals providing dedicated palliative teams, and some hospitals offering only part-time or improvised coverage. Specialized palliative care training exists as a two-year subspecialty, however, it’s available only in six hospitals, leaving a gap in workforce availability. Israel’s system is unique in its universal coverage, access to essential opioids, and supportive services, including the ability to provide home-based equipment and care staff.  Challenges remain primarily in enforcing the 2005 law and 2009 directive in hospitals, expanding early palliative care beyond end-of-life patients, improving training for physicians across specialties, and extending services for children. Despite these gaps, Israel’s legislative framework and healthcare structure provide comprehensive coverage and integration of palliative care, with the main limitations being operational enforcement and specialized  workforce availability. |
| 1. What was the initial motivation for the law, who promoted it and what were its main objectives and implementation challenges? | | |
| The 2018 Ministerial Order No. 253 of the Romanian Ministry of Health originated within a World Bank–funded health sector reform programme, which included specific lines for palliative care, rehabilitation, and long-term care. Under this framework, a national working group was established, composed of clinical palliative care specialists, National House of Insurance specialists and Ministry of Health officials specialised in legislation and policy drafting. The Ministry of Health formally promoted and coordinated the regulation.  The primary objectives were to define palliative care in the Romanian context, differentiate it from terminal care, define and describe palliative care services in various settings and on levels of complexity, set clear eligibility criteria, and establish national service standards and requirements for authorisation. The regulation sought to clarify when palliative care should begin, who should have access, the types and levels of care (basic and complex), and the criteria for service provision across settings. It also aimed to ensure that the term “palliative care” would only apply to services meeting agreed quality standards, thus improving consistency and quality nationwide.  The implementation process faced several challenges. First, bridging the cultural and professional gap between clinical and legislative perspectives proved difficult: at the outset, authorities equated palliative care with end-of-life care, and it required sustained negotiation to establish its role alongside active treatment. Second, drafting the regulation was lengthy and complex, taking over two years plus an additional six months for approval, partly due to the highly detailed nature of the standards. Third, interpersonal and leadership challenges arose within the working group, requiring time to establish mutual respect and equal partnership between technical and legal experts.  Ultimately, the regulation represented a milestone in structuring palliative care provision in Romania, though it was more operational than rights-based, focusing on service organisation rather than formally guaranteeing universal access. | | The initial motivation was to define who is a dying patient and to formalize patient rights at the end of life after decades of advocacy by non-governmental organizations, culminating in  the 2005 Dying Patient Act, which defined dying patients and their rights, though the right to palliative care is not explicitly addressed in the 2005 law text. This was followed by the 2009 palliative care directive, which guaranteed 24/7 multidisciplinary palliative care for end-of-life patients. The 2005 law was promoted by the Israeli Medical Association, National Ethics Committee and Members of Parliament. Implementation challenges include limited hospital-  based services, lack of enforcement by the Ministry of Health, and a small number of hospitals offering specialized training.  **Main Objectives of the Law:**  • Define end-of-life patients and their right, though the right to palliative care is not explicitly addressed in the 2005 law text.  • The 2009 directive aims to guarantee 24/7 multidisciplinary palliative care for end-of- life patients. This was not addressed in the 2005 law.  • Ensure full coverage under national healthcare insurance, at home or in institutions. (This was not addressed in the 2005 law but is highlighted in the 2009 directive.)  • Provide care through a team including physician, nurse, psychologist, and social worker. (This was not addressed in the 2005 law but is highlighted in the 2009 directive.)  **Implementation Challenges:**  • Less than half of hospitals currently provide dedicated palliative care teams.  • Some hospitals rely on part-time or improvised staff rather than full teams.  • Specialized palliative care training is limited to six hospitals, leaving workforce gaps.  • Enforcement of the directive in hospitals has been weak. |
| 2. How does the law promote palliative care in your country? | | |
| The 2018 Ministerial Order No. 253 promotes palliative care in Romania primarily through the establishment of a national framework of definitions, service levels, and quality standards that providers must meet to be authorised and financed as palliative care services. By explicitly distinguishing palliative care from terminal care and defining its provision alongside active treatment, the regulation broadens the scope of services and encourages earlier integration into the patient care pathway.  It operationalises palliative care through clear eligibility criteria, specifications for basic and complex care, and detailed requirements for staffing, equipment, and service delivery models. The regulation applies across care settings, inpatient, outpatient, home-based, and institutional care, and mandates interdisciplinary teams, thus promoting comprehensive, person-centred care.  The law indirectly drives quality improvement by ensuring that only services meeting the standards can be accredited as palliative care providers. This has led to the closure of substandard services and the upgrading of others to comply with the criteria. As a result, the overall quality and consistency of palliative care provision has improved, even if the total number of services initially decreased.  Although the Ministerial Order does not address education or research directly, as these are under the jurisdiction of the Ministry of Education and Research, it supports professional development indirectly by defining staffing requirements that include qualified personnel. Additionally, the regulation creates a governance structure within the Ministry of Health, requiring oversight and coordination at the national level, which has supported subsequent policy developments, including an updated National Strategy for Palliative Care and a financing programme linked to service types and standards.  Overall, its promotional role lies in structuring the sector, setting enforceable quality benchmarks, and embedding palliative care into the national health system framework, even without framing it as an explicit patient right. | | The 2005 law formally recognizes who qualifies as a dying patient, the law makes it clear that end-of-life patients have specific rights to care, decision-making, and symptom management. This legal recognition establishes the ethical and regulatory framework that allows healthcare providers to prioritize palliative interventions.The 2009 directive promotes palliative care by guaranteeing universal access for end-of-life patients, providing multidisciplinary teams, and ensuring that care is fully covered financially. Although initially restricted to end-of-life patients, the directive indirectly encouraged services for a broader population of patients with life-limiting illnesses. The law’s existence has facilitated nationwide organization of services and provided a foundation for expanding home hospice and community-based care.  Analyses by Aspect:  1. Empowering Societies to Care:  • The 2005 law defines a “close person,” as a person who provides for notification to the proxy/family, respects advance directives and representation.  • The 2009 directive has enhanced private-public partnerships to allow agile service implementation, particularly in the community.  2. Supportive Health Policies:  • The directive addresses that a 24/7 care is mandated and financed through national health insurance for all patients with an incurable disease.  3. Research:  • Not addressed in the 2005 law or in the 2009 directive either.  4. Education and Training:  • Education is not addressed in the 2005 law text, however, it’s highly recognized in the 2009 directive.  • In Israel, specialized palliative care is a two-year subspecialty, offered by six hospitals.  • Even though education and training in palliative care are well emphasized in the 2009 directive, still lack of palliative rotations in other residencies limits physician familiarity and referral for consultations. In addition, there is a lack of dedicated palliative care specialist in majority of the hospitals in Israel.  5. Access to Essential Medicines  • In the 2005 law analgesics are mentioned but not specifically opioids or access to opioids. The 2009 directive includes the access to opioids.  6. Integrated Services:  • In the 2005 law text, integration of services is not addressed.  • In the 2009 directive, 24/7 community/home-based services are well recognized and since the directive was enacted, the palliative care services has well developed, providing beds, equipment, and staff 24/7, especially in home-settings.  • Hospital services lag behind, with inconsistent coverage and lack of enforcement and training. |
| 3. Does the law—as it stands—secure the progress made toward ensuring patients’ right to palliative care, and if it does, could you explain how? | | |
| As it stands, the 2018 Ministerial Order No. 253 does not explicitly secure the universal right of patients to access palliative care. Its primary focus is operational: regulating how services should be organised, the standards they must meet, and the requirements for authorisation and functioning. It does not include a formal rights-based provision stating that every person in need of palliative care is entitled to receive it regardless of location or circumstance.  However, the regulation does secure certain aspects of progress indirectly. By establishing national standards, definitions, and service levels, it creates a consistent framework that prevents dilution of quality and ensures that services labelled as palliative care meet agreed requirements. This indirectly protects patients from receiving substandard care under the palliative care label. It also embeds palliative care into the Ministry of Health’s governance structures, ensuring ongoing oversight, accreditation, and alignment with national strategies.  The law’s emphasis on interdisciplinary teams, defined eligibility criteria, and integration of basic and complex care supports the sustainability of quality services once established. The recent development of a financing programme linked to these standards, as well as the updated National Strategy for Palliative Care, builds on this regulatory foundation, suggesting a pathway for gradual expansion and improvement.  Nonetheless, the regulation remains more an instrument for structuring and managing services than a guarantee of access as a patient right. According to expert opinion, a stronger rights-based guarantee would require inclusion in broader legislation, such as the Law on Patients’ Rights, alongside alignment of financing with the established standards to ensure that compliance is both feasible and sustainable. | | The 2005 law define end-of-life patients and their rights, though the right to palliative care is not explicitly addressed in the 2005 law text. The 2009 directive fully secures patients’ rights to palliative care, specifying multidisciplinary care and financial coverage for all insured patients. However, practical progress depends on enforcement in hospitals and specialized training. Community services are exemplary, but hospital services remain insufficient.  Main Limitations of the 2005 law and 2009 directive:  • The 2005 law focuses on defining a dying patient and his/her rights, it does not address undergraduate palliative care education, palliative care training, services integration or medications.  • Limited enforcement in hospitals, resulting in uneven service coverage.  • Specialized training available in only six hospitals, restricting workforce capacity.  • Early palliative care services for patients not yet classified as end-of-life are limited.  • Paediatric palliative care is significantly underdeveloped, with few hospitals providing dedicated teams. Paediatric patients are not addressed in the 2005 law text or in the 2009 directive either.  Additional Highlights:  • Community/home-based services have expanded widely due to efficient HMO structure and private-public partnerships.  • Patients can receive equipment and trained staff at home and the access to home-based palliative care is high nationwide in Israel.  • Universal coverage and funding ensure equity in access, regardless of age, disease, or location.  • Future needs include reinforcement of hospital-based enforcement, physician education, early integration, and expansion of pediatric services. |
| Additional aspects and remarks | | |
| None. | | Future efforts focus on enforcing hospital implementation, expanding early-integrated palliative care, and improving paediatric palliative care. The goal is to ensure full access across hospitals and community services, strengthen physician education, and develop outpatient clinics for patients with life-limiting illnesses even at the earlier phase of the disease trajectory.  Details:  • Reinforce hospital compliance with the 2009 directive to provide 24/7 multidisciplinary palliative care.  • Expand services for patients with life-limiting illnesses beyond end-of-life designation.  • Improve paediatric palliative care, increasing the number of hospitals with dedicated teams.  • Introduce obligatory rotations in palliative medicine for residents in oncology, neurology, nephrology, pulmonology, and other relevant specialties.• Develop community-based palliative care clinics for early integrated care.  • Continue leveraging HMO structure and private-public partnerships to maintain rapid and equitable service delivery. |

# **Supplementary Table S13** Summary of Interviews with Key Informants of Slovakia and Poland.

| Slovakia | | Poland |
| --- | --- | --- |
| Time | *October 10, 2025* | *March 17, 2026* |
| Interviewer | *LM* | *TC* |
| Interviewee | *AS* | *LP* |
| Executive Summary | | |
| Slovakia’s path to advancing palliative care was defined not by a standalone law, but by a strategic and extensive amendment to its existing 2004 Healthcare Provision Act (Act. No. 576). These amendments were driven primarily by Dr. Andrea Škripeková, PhD., a pioneering clinician in the field, who mobilized a small team of volunteer experts including lawyers, data analysts, and fellow physicians. In the absence of strong institutional support, they drafted and advocated for legislative changes that formally recognized and structured palliative care within the Slovak health system.  These legislative changes emerged in the wake of the COVID-19 pandemic and were strategically embedded into Slovakia’s EU-funded Recovery and Resilience Plan. This allowed palliative care to be framed as a national health priority and milestone, unlocking over €20 million in European Union financial support. The amended law now includes specific definitions of basic and specialized palliative care, establishes multidisciplinary care requirements, improves communication standards, and links funding to data and outcome reporting. However, implementation remains uneven, and funding mechanisms are still largely dependent on secondary legislation and negotiation with health insurance companies.  While the law does not explicitly declare palliative care a fundamental patient right, it has transformed the visibility and perceived legitimacy of palliative care. It has significantly advanced education and certification among healthcare professionals and led to the development of pilot hospital-based palliative care teams. Persistent challenges include resistance from long-standing NGO-led providers, insufficient integration between hospital and community services, and a lack of formal quality control and research mandates.  Key Highlights from the Interview   - Palliative care was integrated into the 2004 Healthcare Provision Act (Act No. 576/2004 Coll) through newly drafted sections, rather than as a new law. - The legislative reform was led by a small unpaid group of volunteers, including clinicians, lawyers, and analysts, coordinated by Dr. Škripeková. - The reform leveraged the EU Recovery and Resilience Plan, resulting in over €20 million in funding for palliative care infrastructure and services. - Definitions of basic and specialized palliative care, multidisciplinary teams, communication protocols, and reimbursement structures were introduced. - Medical education and certification in palliative care increased significantly following the law’s adoption. - The main implementation challenges include resistance from traditional service providers, lack of cross- sector integration, and uneven quality of care delivery. | | Palliative care in Poland has developed through a strong bottom-up hospice movement, beginning in the 1980s, initially led by volunteers, associations, and organizations often linked to the Catholic Church. These early initiatives highlighted the need for standardization, quality improvement, and integration into the healthcare system, which eventually led to legislative action. The first major regulatory step was taken in 2009, followed by an updated regulation in 2013, issued by the Minister of Health. These regulations established organizational standards, staff requirements, and clearer eligibility criteria, and formally incorporated palliative care as guaranteed healthcare service into the national healthcare system.  The law defines palliative care as a guaranteed healthcare service, financed through public funds, and aligned with the WHO definition of palliative care. This has significantly improved visibility, standardization, and public awareness, and contributed to the expansion of palliative care services across the country. The inclusion of perinatal palliative care in later amendments further broadened the scope of services.  Despite these advancements, the system faces several challenges. The right to palliative care is not explicitly stated in the law, although related patient rights (e.g., pain relief, dignity at the end of life) are recognized elsewhere. Access remains uneven, with patients sometimes dying while waiting for services, and outpatient care is not always available. The law focuses primarily on specialist palliative care, with limited regulation of the broader palliative care approach across the healthcare system. Additionally, hospital-based palliative care teams are not formally regulated or funded, and access to outpatient services is restricted by referral criteria.  Overall, the Polish framework has significantly advanced palliative care by ensuring financing, standardization, and system integration, but further development is needed to improve access, expand service models, and strengthen implementation across settings. |
| 1. What was the initial motivation for the law, who promoted it and what were its main objectives and implementation challenges? | | |
| The motivation for legislative change originated from Dr. Škripeková's concerns regarding the systemic neglect of palliative care, particularly in rural and underserved areas like Eastern Slovakia. After becoming Chief Medical Officer at the Ministry of Health in 2020, she identified the EU Recovery and Resilience Plan as an opportunity to promote palliative care as a national priority. With backing from a State Secretary Peter Stachura (also a palliative care physician with experience in Germany), and later the Prime Minister Eduard Heger, she submitted a proposal for legislative amendments.  Support from national organizations was initially minimal. The National Palliative Care Society existed but was inactive in legal advocacy. Instead, Dr. Škripeková assembled a dedicated team of volunteer collaborators—informally referred to as the "Avengers"—to help draft the proposal and steer it through the political process. The team conducted extensive negotiations with the Ministry of Finance, insurance companies, and various political parties to achieve cross-party support.  The law was passed after nine months of debate, during a time of political instability following the COVID-19 crisis. It was viewed not as a partisan issue, but as a socially necessary reform. Dr. Škripeková emphasized that every citizen, regardless of political affiliation, would eventually require palliative care.  In terms of objectives, the law aimed to:   - Recognize and define palliative care, both basic and specialized, within the national health system. - Integrate multidisciplinary teams (including social workers and psychologists) into palliative care. - Establish referral pathways and require improved communication with patients about care goals. - Reform reimbursement practices to reflect the real costs of palliative care services. - Secure structural funding via EU mechanisms and align national priorities with long-term health strategies.   In summary, the implementation challenges were:   1. Fragmentation and variability in service delivery, particularly among NGO-run mobile hospices. 2. Insufficient funding and delayed implementation of reimbursement reforms. 3. A lack of standardized quality controls and performance monitoring. 4. Resistance to change from long-standing providers who were reluctant to adopt medicalized models of care. 5. Professional burnout and a lack of institutional support for key reform leaders. | | Palliative care in Poland began to develop in the 1980s, largely as a result of grassroots initiatives, many of which were closely associated with the Catholic Church. Many services operated as voluntary associations. The activities of the emerging hospice movement led to the gradual incorporation of hospices into the national healthcare system and to the introduction of public funding for these services from the 1990s onwards.  Alongside these developments, organisations representing the hospice movement began to advocate for the establishment of organisational standards for palliative care services for both adults and children. Key organisations involved in these efforts included the National Hospice Movement Forum, the Polish Association for Palliative Care, the Polish Society of Palliative Medicine, and the Polish Society of Palliative Care Nursing, as well as the Warsaw Hospice for Children and the National Forum for Paediatric Palliative Care. Efforts to establish a legal framework for palliative care were also supported by the Commissioner for Human Rights (Ombudsman) and the Commissioner for Children’s Rights. As a result of advocacy by a wide range of organisations, the Ministry of Health initiated work on a draft regulation, which was ultimately adopted in 2009. The content of this regulation was criticised by organisations involved in palliative care, particularly those representing paediatric palliative care, for failing to incorporate a number of their proposals and comments submitted during the consultation process.  Despite these concerns, the adoption of the 2009 Regulation, on which the currently applicable Regulation of 2013 was subsequently based, represented a major milestone in the development of a legal framework for palliative care in Poland. The regulation aimed to define organisational standards for palliative care services and to establish the conditions governing access to such care, compliance with which is required in order to obtain public funding through the National Health Fund. It also introduced staffing requirements, which contributed to the further development of education and training programmes in palliative medicine and palliative care nursing,  Main Objectives of the Law:   - Integrate palliative care into the national healthcare system. - Establish organizational and staffing standards for palliative care services. - Ensure stable and continuous public financing. - Define clear eligibility and referral criteria for patients. - Promote quality and standardization of care.   Implementation Challenges:   - Some stakeholder proposals, especially from hospice organizations, were not fully incorporated into the law. - Access gaps remain, with patients sometimes dying while waiting for care. - Outpatient palliative care is not consistently available. - The system focuses mainly on specialist palliative care, with limited integration of a broader palliative approach. |
| 2. How does the law promote palliative care in your country? | | |
| The legislative reform in Slovakia introduced clear definitions for basic and specialized palliative care, along with long-term, follow-up, and acute care, by amending the 2004 Healthcare Provision Act. These definitions, including the addition of palliative care to section 9-2, aimed to formally recognize palliative care within the national health system and clarify who is responsible for its provision and under what circumstances. This legal clarity supports the development of clinical standards and ensures that patients are more reliably identified and referred to appropriate services. It also helps medical professionals better understand their responsibilities in delivering end-of-life care.  To improve care delivery, the law also included provisions on how patients should be referred to palliative care specialists. This was crucial because, as Dr. Škripeková explained, many patients were not aware they were eligible for palliative care, which complicated communication about care goals. A separate section was added to address the communication of information content between providers and patients.  These legislative inclusions were modelled after the legal frameworks of countries like Germany and Austria. The Slovak team studied these examples and adapted definitions and structures to fit the local context.  One of the law's innovative elements is its mandate for multidisciplinary teams, including not only healthcare providers but also social workers and psychologists. This holistic approach recognizes the complex needs of patients and families and aims to foster collaboration across different sectors. These provisions are particularly important in a system where mobile hospice care had previously been largely dominated by non-medical providers and where medical professionals were often absent from home-based services. This change followed significant discussion and was seen as important for ensuring professional quality in service delivery.  Crucially, the legislative process itself raised awareness and sparked national debate about the importance of palliative care. Media coverage, public discussions, and cross-party political support helped bring the issue into the spotlight. As a result, the law's impact extends beyond legal structure to cultural change, encouraging healthcare institutions, professionals, and the broader public to recognize the value of high-quality end-of-life care.  Based on the key informant’s interview, the contribution of the law in each dimension of palliative care development can be summarized as follows:  1. Empowering Societies to Care  The law increased visibility and public awareness of palliative care. With media coverage and political debate, the conversation around end-of-life care expanded. By defining palliative care legally and mandating communication protocols, the law indirectly empowered patients and families to understand and request these services earlier.  2. Supportive Health Policies  Structural policies were established, including a dedicated budget line for palliative care ("Excel window"). While specific funding percentages are not fixed in the law, secondary legislation on pricing measures updated biannually have improved service reimbursement. Funding from the EU has been used to develop infrastructure, support hospices, and create pilot hospital-based teams.  3. Education and Training  There has been a dramatic rise in interest in palliative care education. In 2020, only 2–3 physicians applied for postgraduate certification; by 2022, 23 had enrolled in. Attendance for both online and onsite courses have grown. The key informant now leads postgraduate education efforts in the field. Nonetheless, formal integration into undergraduate and specialist training remains underdeveloped.  4. Research in Palliative Care  The law does not address research. All research-related progress depends on voluntary academic and clinical initiatives.  5. Access to Essential Medicines  This was not explicitly addressed within the scope of this legislation.  6. Access to Specialized Palliative Care The key informant explained that certain medical procedures such as palliative sedation and abdominal fluid drainage at home with ultrasound are available in some care settings (specialized). She emphasized that the education level of the physician significantly affects the ability to perform such interventions, leading to variability in service delivery across providers. Specialized teams with proper training are more likely to offer comprehensive medical care, while others, particularly some mobile hospices, may lack this capacity.  7. Development of Integrated Services  However, integration remains inconsistent. Most mobile hospices are NGO-run and frequently lack medical staff. Hospital-linked mobile teams are being piloted, but collaboration between sectors is limited. The key informant has developed national clinical algorithms for identifying palliative patients and initiated hospital integration strategies. | | The establishment in Poland of a separate legal act devoted exclusively to palliative care constitutes an important form of institutional recognition and promotion of this field within the healthcare system. It distinguishes palliative care from other health services, as only selected areas of healthcare in Poland are regulated by dedicated legal acts, whereas most services are governed by broader regulations covering multiple medical fields, such as hospital treatment or specialist outpatient care.  The Regulation also serves as a source of information for healthcare professionals working in different healthcare settings, as well as for patients and their families, regarding the principles governing access to palliative care. In addition, the legal obligation placed on public authorities to publish generally binding legal acts ensures universal access to this information.  Furthermore, the concise definition of palliative care included in the regulation, which refers to the definition adopted by the World Health Organization (WHO), contributes to raising awareness and promoting a broader understanding of palliative care.  Analyses by Aspect:   1. Empowering Societies to Care:   • The hospice movement and associated organizations played a central role in shaping the law and continue to influence development.  • Public discussions, media engagement, and involvement of institutions like the Ombudsman increased awareness.   2. Supportive Health Policies:   • Palliative care is recognized as a separate and guaranteed service within the healthcare system.  • Financing is secured through public funds, supporting service expansion.   3. Research:   • Not included in the Regulation; research is addressed in separate legal frameworks.  • No dedicated national programs for palliative care research.   4. Education and Training:   • Training requirements for staff are included in organizational standards.  • However, palliative medicine is a separate and mandatory subject only at some medical universities.   5. Integrated Services:   • Palliative care is integrated as a distinct service within the healthcare system, rather than being part of other healthcare services.  • Various service types (including home and inpatient care) are included.  • However, hospital-based palliative care teams are not formally regulated or funded. |
| 3. Does the law—as it stands—secure the progress made toward ensuring patients’ right to palliative care, and if it does, could you explain how? | | |
| While palliative care is not explicitly defined as a fundamental legal right, the legislative framework has facilitated meaningful and sustained progress. It institutionalizes funding, improves service structures, and has mainstreamed palliative care within national healthcare discussions. By including palliative care in national and EU-level planning documents, the law helps secure continuity of support.  The introduction of hospital-based pilots and support for staffing from EU funds are critical components of ongoing implementation. Efforts to include social workers and community linkages in care teams show a holistic approach. The development of national patient identification protocols further supports systemic integration.  Major limitations of the law include:   - Lack of an enforceable right to palliative care - Inconsistent quality among providers, particularly in NGO-run settings. - Lack of standardized monitoring and quality assurance mechanisms. - No legal support for research or medication access provisions. - Continued dependence on external funding for staff and infrastructure. | | The adoption of a legal act regulating eligibility criteria and the organisation of palliative care services in Poland has undoubtedly represented an important step forward in improving access to palliative care. The Regulation adopted in 2013 replaced the earlier Regulation of 2009, which had recognised palliative and hospice care services as guaranteed services financed from public funds and had established organisational standards for their provision. The aim of these standards was to ensure the appropriate quality of specialist palliative care delivered in home-based, inpatient hospice and outpatient palliative care clinic settings.  Most of the provisions contained in the repealed 2009 Regulation were retained in the 2013 Regulation. In addition, new provisions were introduced to further improve access to palliative care. In particular, these changes included the expansion of services provided within home hospice care and the introduction of a more up-to-date definition of palliative care reflecting the contemporary definition adopted by the World Health Organization (WHO), thereby promoting a broader understanding of the scope of palliative care.  The diversity of palliative care settings—home-based, inpatient and outpatient—together with the subsequent introduction of perinatal palliative care through later amendments to the Regulation, has created greater opportunities to tailor support to the needs and preferences of patients. At the same time, it introduced a new form of support in the form of perinatal palliative care, which had previously not been provided on such a scale or publicly financed within the Polish healthcare system.  Main Limitations of the Law:   - The right to palliative care is not explicitly stated in the regulation. - Access remains limited, with waiting times and service shortages. - Lack of hospital-based palliative care teams in formal regulation and funding. - Limited access to outpatient palliative care due to restrictive referral criteria. - Focus on specialist services, with insufficient regulation of general palliative care approaches.   Additional Highlights:   - Palliative care is now clearly distinguished from long-term care, improving visibility. - Inclusion of services in official lists enables institutional responsibility and coordination. - National Health Fund provides information services to guide patient access. - Pediatric palliative care is partially included but still does not meet all expectations. - Development of education and research is limited by lack of dedicated regulation. |
| Additional aspects and remarks | | |
| The conversation with the key informant revealed several important aspects of Slovakia’s palliative care law and its implementation that were not explicitly addressed in the previous questions:  Volunteer-Driven Legislative Process  The key informant emphasized that the legislative process was driven by a voluntary group of professionals, including lawyers, economists, data analysts, and clinicians. They worked pro bono and met in informal settings, such as private homes, to draft the legislation.  Personal Sacrifice  She also shared that despite her efforts, she experienced personal and professional frustration and burnout. She described the process as exhausting and stated that she had to make sacrifices, including stepping away from academic pursuits to focus on legislative work.  Growth in Education and Training  Following the adoption of the law, there was a noticeable increase in interest among physicians in palliative care education. The key informant, who leads postgraduate training, reported a significant rise in enrolment for certification programs in palliative medicine.  Independent Leadership and Implementation Work  They key informant also noted that she continues her work independently of the national medical society’s board. She currently leads efforts to develop a hospital-based palliative care centre in Bratislava and collaborates with private hospitals to implement of palliative care programs. This includes developing tools like an algorithm for identifying patients in need of palliative care based on the SPICT (Supportive and Palliative Care Indicators Tool) protocol and promoting connections between hospitals and community-based services, including mobile hospices.  Ongoing Development of Integrated Services  She mentioned that the development of integrated services is ongoing, with the goal of linking acute hospital settings to mobile hospices and improving identification and communication practices for palliative care patients. These initiatives reflect a continued commitment to expanding palliative care access and improving service delivery across all healthcare sectors. | | At present, patient eligibility for palliative care is determined on the basis of a list of diseases included as an annex to the 2013 Regulation. It has therefore been proposed that this list should be expanded to include additional advanced conditions that are not currently specified, in order to enable a greater number of individuals with palliative care needs to access specialist palliative care.  A potentially beneficial amendment to the regulations governing eligibility for specialist palliative care would involve placing greater emphasis on the assessment of symptoms rather than on the stage of a strictly defined disease.  Other proposed legal changes include the introduction, within the provisions of the 2013 Regulation, of hospital palliative care support teams that would provide specialist palliative care consultations for patients admitted to hospitals.  Another proposal currently under discussion concerns improving access to outpatient palliative medicine clinics by revising the eligibility criteria. At present, these criteria are the same as those applied to home-based and inpatient hospice care, which may limit access to specialist palliative care for patients at earlier stages of chronic illness who are already experiencing pain and other distressing symptoms.  A further reform that could improve access to palliative care would be the explicit recognition in legal provisions of additional levels of palliative care beyond specialist palliative care, particularly the palliative care approach applied across the healthcare system as a whole. Another important step, emphasising the significance of palliative care, would be the introduction of a statutory provision explicitly recognising the right to palliative care. |
